# Determination of non-volatile metabolic profiles and their sensory relevance in different grades of brandy through widely targeted metabolomics

**DOI:** 10.1016/j.fochx.2026.103889

**Published:** 2026-04-21

**Authors:** Yue Ma, Yuhao Liu, Baochun Zhang, Chunhua Shen, Lina Yu, Yan Xu, Ke Tang

**Affiliations:** a Lab of Brewing Microbiology and Applied Enzymology, School of Biotechnology and Key Laboratory of Industrial Biotechnology of Ministry of Education, Jiangnan University, Wuxi 214122, PR China; b China Key Laboratory of Microbiomics and Eco-brewing Technology for Light Industry, Key Laboratory of Industrial Biotechnology of Ministry of Education, State Key Laboratory of Food Science and Technology, Jiangnan University, 1800 Lihu Avenue, Wuxi, Jiangsu 214122, PR China; c ChangYu Group Company Ltd., Yantai, Shandong 264000, PR China

**Keywords:** Brandy, Grade, Oral sensation, Metabolomic, Non-volatile compounds

## Abstract

This study investigated the sensory characteristics and non-volatile chemical composition of different grades of brandy using sensory descriptive analysis and widely targeted metabolomics. Significant improvements in sensory attributes, particularly oral sensation-related qualities such as smoothness, sweetness, burn, and astringency, were observed with increasing grade. A total of 682 non-volatile metabolites, dominated by phenolic compounds (44.58%) and lipids (11.88%), were identified. Grade-dependent variations in metabolite composition were revealed, with 164 compounds identified as grade-specific biomarkers. Of these, 112 metabolites were linked to taste perception, including some phenols contributing to bitterness, some saccharides and phenolic esters contributing to sweetness. This study provides a comprehensive chemical profile of brandy's sensory quality and highlights the grade difference in shaping its flavor.

## Highlights

• Sensory-metabolomic integration identifies chemicals determining brandy grades.
• Non-volatiles differentiate brandy grades and influence sensory quality attributes.
• Oak-derived phenolic esters/coumarins enhance sweet/bitter profiles in brandies.
• Phenolics, saccharides, and organic acids were correlated with oral sensations.
• Metabolomic markers offer a solution for brandy authentication and grading.

## 1 Introduction

Brandy, one of the six distilled spirits, is favored by consumers for its rich aroma, unique taste, and complex mouthfeel, with its production involving grape fermentation, distillation, oak barrel maturation, and blending (Tsakiris, Kallithraka, & Kourkoutas, 2014). Originating in Cognac, France, brandy has become popular globally, with market grades such as Very Special (VS), Very Superior Old Pale (VSOP), and Extra Old (XO) defined by maturation time in oak barrels, with higher grades indicative of longer ageing and more complex sensory profiles (Y. Ma et al., 2024).

The sensory quality of brandy, particularly its oral sensory characteristics, is of paramount importance in determining its overall quality and appeal. These sensory attributes are significantly influenced by the non-volatile compounds present in the brandy, which are closely related to its interaction with oak barrels during maturation. The extraction of wood-derived compounds, such as phenols, sugars, and terpenoids, plays a crucial role in shaping the chemical composition and sensory profile of brandy (Flamini, Panighel, & De Marchi, 2023). Several non-volatile compounds have been identified in brandy through previous research, including low molecular weight phenols and ellagitannins, which are influenced by factors such as oak species, barrel-making techniques, and the maturation process (Canas, 2017). Additionally, monosaccharides, including glucose, arabinose, and fructose, have been detected in brandy, with their concentrations significantly elevated due to prolonged maturation (Montero, Dodero, Sánchez, & Barroso, 2005). Furthermore, certain non-volatile compounds, particularly those recognized as taste-active, have been associated with the oral sensory perception of brandy. For example, the discovery of dihydrodehydrodiconiferyl alcohol and brandy tannin A, both phenolic compounds, highlights their role in enhancing the sweetness of aged brandies (Winstel, Bahammou, Albertin, Waffo-Téguo, & Marchal, 2021; Winstel, Capello, Quideau, & Marchal, 2022). Similarly, the presence of lignans, such as lyoniresinol, and bitter bartogenic acid analogues derived from triterpenoids might contribute to the bitterness of oak-aged brandy (Gammacurta, Waffo-Teguo, Winstel, Dubourdieu, & Marchal, 2020; Winstel & Marchal, 2019). While these studies provide valuable insights into the composition of brandy and the chemical basis of its sensory experiences, they predominantly focus on specific non-volatile compounds, leaving a gap in comprehensively understanding the overall chemical composition and its contribution to the complex sensory perceptions of brandy. A more holistic approach is warranted to fully elucidate the intricate relationships between brandy's chemical profile and its sensory attributes.

Metabolomics, a high-throughput analytical tool with high sensitivity and resolution, has shown great potential in addressing these gaps. It has been effectively applied to study alcoholic beverages, such as wine and whisky, to elucidate their chemical profiles (Arbulu, Sampedro, Gomez-Caballero, Goicolea, & Barrio, 2015) and oxidation mechanisms (Ontanon et al., 2020; Roullier-Gall et al., 2020). In brandy, metabolomics has enabled researchers to isolate bitterness or sweetness-enhancing compounds, such as oak coumarins (Winstel, Gautier, & Marchal, 2020), dihydrodehydrodiconiferyl alcohol (Winstel et al., 2021) and brandy tannin A (Winstel et al., 2022), providing new insights into the chemical basis of sensory perception. However, no studies have systematically integrated metabolomics with sensory analysis to comprehensively characterize the chemical composition and sensory attributes of different brandy grades.

To address these gaps, this study presents a systematic integration of descriptive sensory analysis with widely targeted metabolomics to decode the chemical basis of sensory quality across distinct brandy grades (VS, VSOP, and XO). Our approach was designed to achieve three interconnected objectives: (1) to establish the sensory profile that differentiate brandy grades; (2) to characterize the non-volatile metabolic landscape and identify grade-dependent chemical markers; and (3) to search taste-active compounds that underpin specific oral sensory attributes, leveraging a dedicated taste-active compound database. By bridging the gap between chemical complexity and sensory perception, this work should provide a comprehensive understanding of the chemical mechanisms underlying the sensory appeal of brandy and its quality differentiation, and offer an analytical framework applicable to the broader field of flavor chemistry, with potential implications for quality authentication and maturation monitoring in spirit production.

## 2 Materials and methods

### 2.1 Samples and chemicals

Four types of commercially available brandies were studied, with three independently sourced bottles obtained for each type to account for batch-to-batch variability. All brandies were made from *Vitis vinifera* cv. Ugni Blanc and produced by Changyu Winery (Yantai, China) and had a uniform alcohol content of 40% (*v*/v). The samples comprised distinct ageing grades: CVS (VS grade, Very Special), CVSOP (VSOP grade, Very Superior Old Pale), CXO10 (XO grade, Extra Old) with 10 years of maturation, and CXO15 (XO grade, Extra Old) with 15 years of maturation. All brandies underwent ageing in Limousin oak barrels (France). All samples were stored upright in their original sealed bottles at 20 ± 2 °C in the dark until analysis to minimize light-induced degradation and evaporation.

Methanol and acetonitrile were purchased from CNW Technologies GmbH (Shanghai, China). Formic acid was purchased from Sigma-Aldrich (Shanghai, China). The water used in the study was purified using a Merck Millipore water purification system (Merck Millipore, USA).

### 2.2 Sensory evaluation of different brandy samples

#### 2.2.1 Subjects

The sensory panel was selected based on the criteria outlined by published method (Y. Ma et al., 2024), focusing on participants' ability to identify and quantify brandy flavor characteristics, including aroma, taste, and mouthfeel. Twelve assessors (5 males and 7 females, aged from 18 to 25 years old) with superior sensory acuity and prior experience in previous alcoholic beverage sensory evaluation were recruited from Jiangnan University. All participants provided written informed consent, approved by the Jiangnan University Institutional Review Board (JNU20230601IRB24), and were compensated for their participation according to the experimental duration.

#### 2.2.2 Panel training

The whole training protocol was divided into three stages: conceptual familiarization, reference standardization, and blind validation, following established guidelines for descriptive sensory analysis (Lawless & Heymann, 2010). During the first phase, participants were introduced to the production and sensory lexicon definition of brandy, with a focus on taste (sweetness, sourness, bitterness) and mouthfeel attributes (astringency, alcohol burn, smoothness, viscosity), as adapted based on a combination of pre-tasting experience and literature (Louw et al., 2013). In the second phase, which lasted a period of 2 months (twice a week), participants were trained to recognize and rate the intensity of each sensory attribute using standardized reference solutions. The reference solutions were prepared in water-ethanol (40% *v*/v) matrices to mimic brandy base conditions. The following concentration ranges were used: sucrose (0, 5, 10, 20, 30 g/L) for sweetness; citric acid (0, 0.5, 1.0, 1.5, 2.0 g/L) for sourness; caffeine (0, 0.3, 0.6, 0.9, 1.2, 1.5 g/L) for bitterness; alum (0, 0.5, 1.0, 2.0, 3.0 g/L) for astringency; ethanol gradients (0%, 10%, 20%, 30%, 40%, v/v) for alcohol burn; and glycerol (0, 0.5, 1.0, 2.0, 3.0 g/L) in 40% ethanol for smoothness. Participants practiced rating these references on the same 6-point scale used in the formal evaluation. The 6-point scale was chosen based on preliminary trials showing that panelists could reliably differentiate intensities within this range. The scale was anchored with reference standards during training, and performance was monitored using PanelCheck software. During the final validation stage, participants underwent attribute identification tests with nasal clips to eliminate olfactory interference, and their performance was assessed for stability and reproducibility using PanelCheck software (Tomic et al., 2010). Assessors were required to achieve a repeatability standard deviation<0.8 before being permitted to participate in the formal evaluation.

#### 2.2.3 Formal evaluation

The trained panel conducted the formal experiment to evaluate the intra-oral perception of different brandy samples of different grades following our established sensory evaluation protocols (Yue Ma et al., 2023). Brandies were presented in 2 mL aliquots in ISO-standard glasses labeled with random 3-digit codes, ensuring a double-blind presentation. Participants were required to wear nasal clips to eliminate olfactory interference, taste the samples by coating them across the tongue for 10 s, and then spit out or swallow the sample consistently. The use of nasal clips was intentional to isolate intra-oral sensory attributes (taste and mouthfeel) from olfactory influences, as the primary goal of this study was to link non-volatile metabolites to oral perception. They rated the intensity of predefined sensory attributes, namely sweetness, sourness, bitterness, astringency, alcohol burn, smoothness and viscosity, on a 6-point scale (0 = absent; 6 = extreme). Following each tasting, participants rinsed their mouths with water and unsalted crackers, followed by a 5-min rest period. All experiments were conducted in a sensory chamber at 23 °C, with separate booths to minimize external distractions.

### 2.3 Use of widely targeted metabolomics for detecting the metabolites in brandies of different grades

#### 2.3.1 Sample preparation and metabolite extraction

Each brandy sample (6 mL) was processed through a standardized extraction protocol to ensure consistency and reproducibility. Prior to freeze-drying, samples were stored at −80 °C. The freeze dryer (Labconco, USA) was pre-cooled for 2 h until the shelf temperature reached −30 °C, after which samples were placed horizontally in the drying chamber. Under vacuum (15 Pa), samples underwent lyophilization for 36 h with a gradual temperature ramp to ambient conditions, while the cold trap maintained a constant −80 °C to efficiently sublime water. This approach ensured consistent and stable sample drying across all grades. Then the samples were reconstituted in 1 mL of 70% methanol (*v*/v) containing an internal standard (2-chloro-*L*-phenylalanine, 1 mg/L). The mixture solution was subjected to vortex mixing for 15 min to ensure homogeneity, followed by sonication in an ice-water bath for 10 min to enhance metabolite extraction. The mixture solution was then centrifuged at 12,000 rpm for 10 min at 4 °C using an Eppendorf 5425 centrifuge. The supernatant was carefully collected and filtered through a 0.22 μm membrane to ensure compatibility with liquid chromatography–tandem mass spectrometry (LC–MS/MS) analysis.

#### 2.3.2 LC–MS/MS analysis

The metabolites in the brandy samples were analyzed following Chen's method (W. Chen et al., 2013) using an LC–MS/MS system (HPLC: SCIEX ExionLC™ AD; MS: Applied Biosystems 4000 Q TRAP) equipped with an SB-C18 analytical column (Agilent, 1.8 μm, 2.1 mm × 100 mm). To ensure analytical reproducibility and data quality, a pooled quality control (QC) sample was prepared by mixing equal volumes of all brandy samples (CVS, CVSOP, CXO10, CXO15). The QC sample underwent the same extraction protocol as the test samples and was injected periodically throughout the analytical sequence (every 10 injections) to monitor instrument stability and signal drift. QC samples were also used for method conditioning prior to the analytical run. The mobile phase consisted of two components: aqueous solution A (0.1% formic acid, *v*/v) and organic solution B (acetonitrile containing 0.1% formic acid, v/v). A gradient elution program was applied as follows: 0–9 min (5%–95% B), 9–10 min (95% B), 10–11 min (5% B), and 11–14 min (5% B). The system was operated at a flow rate of 0.35 mL/min with an injection volume of 2 μL, and the column and autosampler temperatures were maintained at 40 °C and 4 °C, respectively.

The mass spectrometer (Agilent, USA) was configured in positive and negative ion modes to maximize ionization efficiency. The ion source was equipped with an atomizer to facilitate droplet formation, and the following parameters were used: IonSpray Voltage of +5500 V in positive mode and − 4500 V in negative mode, Curtain Gas at 25 psi, Temperature at 500 °C, Ion Source Gas 1 at 50 psi, and Ion Source Gas 2 at 60 psi. Metabolite identification was achieved using a triple quadrupole (QQQ)–linear ion trap (LIT) hybrid system, where multiple reaction monitoring (MRM) was optimized for each metabolite to determine declustering potential (DP) and collision energy (CE). Qualitative analysis relied on retention time, parent ion (Q1), daughter ion (Q3), DP, and CE values, which were cross-referenced with a self-built database (MWDB, metware database) and public repositories (MassBank, KNApSAck, HMDB, MoTo DB, and METLIN). To ensure transparency regarding identification confidence, we classified the identified metabolites into three levels based on the accuracy of matching, as detailed in Supplementary Table S1. As this study focused on comparative metabolomic profiling rather than absolute quantification, semi-quantitative analysis was performed by normalizing the peak areas of the compounds to the internal standard, so calibration curves, LOD, LOQ, and recovery rates were not determined for individual metabolites. The entire workflow, including data acquisition and processing, was executed using SCIEX Analyst Work Station Software (version 1.6.1).

### 2.4 Statistics

Statistical analysis was conducted using a comprehensive suite of software tools to ensure thorough evaluation of sensory and metabolomics data. A radar plot was constructed to visualize sensory analysis results using *OriginPro2023* (version 10.0.0.154, OriginLab Corporation, Northampton, MA, USA). To assess significant differences across groups, a one-way analysis of variance (ANOVA) was performed using *IBM SPSS Statistics 22.0* (IBM Corporation, Armonk, NY, USA), incorporating significance testing with *p*-value thresholds and appropriate corrections for multiple comparisons. Additionally, data were pre-processed through normalization and scaling steps prior to analysis to ensure robust results.

The online bioinformatics platform (https://www.bioinformatics.com.cn) was utilized for generating correlation coefficients, circle packing visualizations, fuzzy c-means clustering (Mfuzz), and a heatmap. These tools enabled the exploration of relationships among metabolites and sensory attributes, with specific focus on Pearson or Spearman correlation coefficients for correlation analysis.

Multivariate statistical analyses, including principal component analysis (PCA) for dimensionality reduction and identification of major variance sources, hierarchical cluster analysis (HCA) for sample clustering, and partial least squares-discriminant analysis (PLS-DA) for classification and variable importance assessment, were conducted using SIMCA software (version 14.1, MSK Data Analytics Solutions, Umeå, Sweden). Prior to PLS-DA, data filtering was applied to reduce the variable-to-sample ratio: compounds with >30% missing values across samples were removed, and only metabolites with relative standard deviation (RSD) < 30% in QC samples were retained. We acknowledge that external test set validation was not performed, which is a limitation of the current study.

## 3 Results and discussion

### 3.1 Sensorial profiles across different brandy grades

**Fig. 1:**
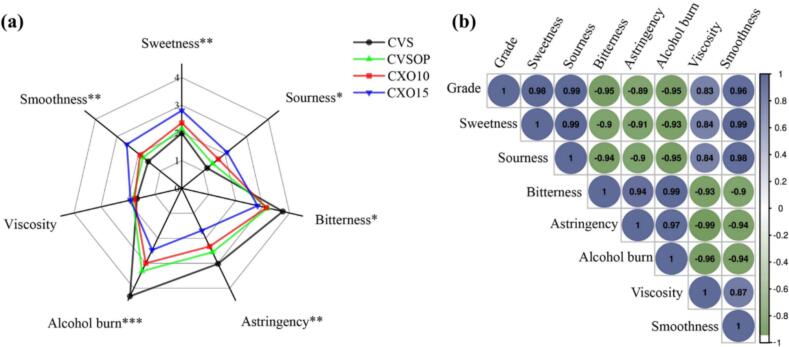
Sensory profiles of brandy across different grades. (a) Radar plot showing the mean intensity scores of seven oral sensory attributes for four brandy grades: CVS (VS grade), CVSOP (VSOP grade), CXO10 (XO grade, 10-year maturation), and CXO15 (XO grade, 15-year maturation). Sensory intensities were rated on a 6-point scale (0 = absent, 6 = extreme) by a trained panel (*n* = 12). Data points represent mean values, and the area enclosed by each polygon reflects the overall sensory profile of each grade. (b) Correlation matrix heatmap showing Pearson correlation coefficients (r) between the seven sensory attributes and brandy grade. The colour scale ranges from dark blue (strong positive correlation, r = +1.0) to dark green (strong negative correlation, *r* = −1.0), with white indicating no correlation (*r* = 0). Cell values represent the correlation coefficients. (For interpretation of the references to colour in this figure legend, the reader is referred to the web version of this article.)

The sensory evaluation of different grades of brandy (CVS, CVSOP, CXO10, CXO15) revealed significant variations in their taste and mouthfeel profiles, as illustrated in Fig. 1a. The lowest grade (CVS) exhibited marked alcohol burn (4.32), astringency (3.02), and bitterness (3.75), while the highest grade (CXO15) displayed significantly enhanced smoothness (2.55), sweetness (2.81), and sourness (2.09). Intermediate grades (CVSOP and CXO10) showed sensory attributes that progressively transitioned between these extremes. Notably, alcohol burn (*p* < 0.001), astringency (*p* < 0.01), smoothness (*p* < 0.01) and sweetness (*p* < 0.01) were the most distinguishing attributes, underscoring the profound impact of grade on mouthfeel perception.

Further statistical analysis via correlation coefficients (Fig. 1b) demonstrated strong associations between sensory attributes and brandy grade (|*r*| > 0.8). As grade increased, scores for sourness, viscosity, and smoothness increased, whereas bitterness, astringency, and alcohol burn decreased. These findings confirm that higher grades, indicative of extended ageing, enhance sensory appeal, aligning with prior research that highlights the role of maturation in developing superior sensory attributes (Caldeira, Mateus, & Belchior, 2006). However, further investigations incorporating chemical profiling could provide deeper insights into the specific compounds driving these perceptual changes. It is important to note that the sensory panel consisted of trained assessors aged 18–25 years who, while experienced in sensory analysis of alcoholic beverages through formal training, may not represent experienced brandy consumers with established ethanol tolerance. Age and prior exposure to spirits could influence sensitivity to attributes such as alcohol burn and astringency. Therefore, the absolute intensity values should be interpreted with this context in mind, although the relative differences across grades remain valid. Besides, the sensory evaluation was conducted with nasal clips to isolate oral perception, which does not reflect real-world brandy consumption where retronasal olfaction significantly modulates attributes such as alcohol burn and smoothness. Future studies should integrate ortho- and retronasal olfaction for a more holistic sensory model.

### 3.2 Non-volatile metabolite profiles of brandies of various grades

#### 3.2.1 Metabolomic profiling and class-specific variations across brandy grades

**Fig. 2:**
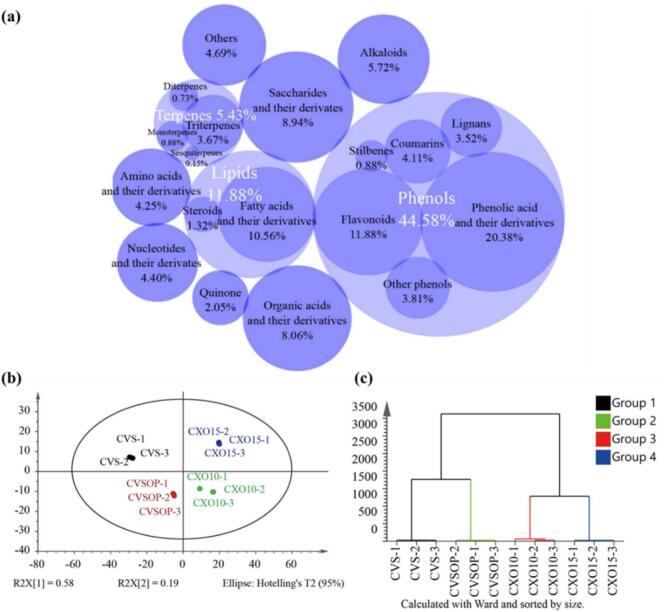
Overview of non-volatile metabolite profiles across brandy grades. (a) Circular packing chart illustrating the composition and relative abundance of 682 identified non-volatile metabolites classified into 10 structural categories. The area of each sector is proportional to the number of compounds in that category. (b) Principal component analysis (PCA) score plot of metabolite profiles across four brandy grades (*n* = 3 biological replicates per grade). Each point represents an individual sample. (c) Hierarchical cluster analysis (HCA) heatmap showing sample clustering based on metabolite abundance.

A comprehensive non-targeted metabolomic analysis using LC–MS/MS identified 682 non-volatile metabolites in brandies of varying grades (Supplementary Table S1), categorized into 10 structural classes (Fig. 2a). Representative chromatograms of quality control (QC) samples and mass spectra for key compounds are provided in Supplementary Fig. S1 and Fig. S2. The consistent overlay of QC chromatograms (Fig. S1c) confirms the high reproducibility of the analytical method. Non-volatile metabolite profiling revealed distinct compositional patterns across brandy grades (Fig. 2a). Phenolic compounds dominated (44.58%, *n* = 304), with phenolic acids (*n* = 139) and flavonoids (*n* = 81) constituting the majority, followed by lipids (11.88%, n = 81; including fatty acid derivatives), saccharides (8.94%, *n* = 61), organic acids (8.06%, *n* = 55), alkaloids (5.72%, *n* = 39), terpenoids (5.43%, *n* = 37; e.g., mono-, di-, and sesquiterpenes), nucleotides (4.40%, n = 30), amino acids (4.25%, *n* = 29), quinones (2.05%, *n* = 14), and other minor constituents (4.69%, *n* = 32). These components collectively represent the non-volatile chemical repertoire of brandy, highlighting its compositional complexity driven by phenolic diversity, lipidic components, and terpenoid subclasses. It should be noted that while the 70% methanol extraction successfully captured a broad range of metabolites, this method was not systematically optimized for all compound classes. Less polar compounds such as lignans (*n* = 24 detected) and triterpenes (n = 37 detected) may have lower recovery compared to more polar ones, potentially leading to underestimation of their absolute abundances. However, because all samples were processed under identical extraction conditions, the comparative analyses and grade-dependent trends for these compound classes remain valid.

Prior to multivariate analysis, log transformation and unit variance scaling were applied to normalize metabolite abundance. Unsupervised PCA and HCA revealed distinct clustering patterns (Fig. 2b, c). XO-grade samples segregated along the first principal component (PC1), while CVS/CVSOP and CXO10/CXO15 formed cohesive subgroups in HCA. These patterns aligned with sensory attribute differentiation observed in radar charts (Section 3.1), where higher-grade brandies exhibited distinguished sensory descriptors. The metabolic heterogeneity paralleled sensorial profiles, suggesting that non-volatile metabolites underpin sensory quality gradations.

#### 3.2.2 Cluster analysis of metabolic profiling revealing grade-dependent changes in brandy

**Fig. 3:**
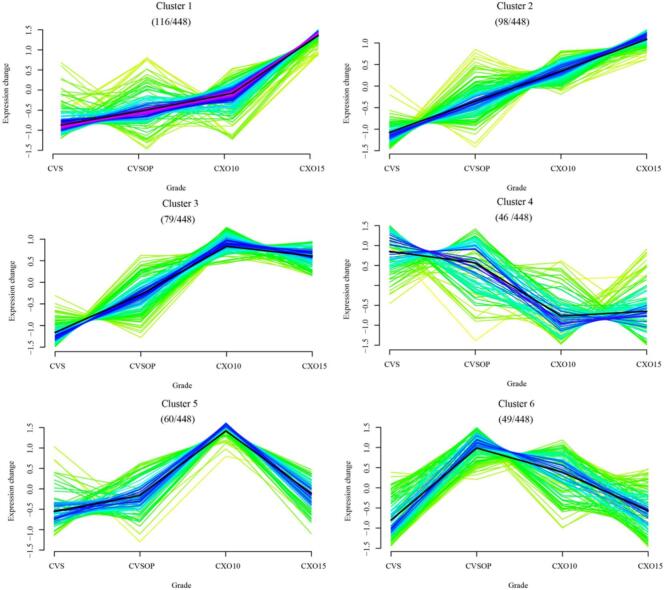
Overview of metabolite accumulation patterns across four brandy grades (CVS, CVSOP, CXO10, CXO15) by Mfuzz clustering analysis. The 448 significant metabolites (*p* < 0.05) were assigned to six clusters according to Mfuzz clustering analysis. Each line indicates the relative abundance (*Z*-score normalized) of each metabolite and the bold line represents the mean expression pattern of each cluster.

To investigate the metabolic changes associated with the brandy grading, a comprehensive metabolomic analysis was performed, identifying 448 significant metabolites (*p* < 0.05) that were subsequently grouped into six clusters using Mfuzz analysis (Supplementary Table S1). These clusters revealed distinct patterns of metabolite accumulation across different brandy grades (Fig. 3). Clusters 1, 2, and 3 demonstrated progressive enrichment with increasing grade, albeit with varying trajectories: Cluster 1 exhibited gradual accumulation in lower grades and a sharp increase in higher grades, Cluster 2 showed a uniform rise across all grades, and Cluster 3 increased steadily in mid-range grades before plateauing in premium grades. Clusters 4, 5, and 6, on the other hand, displayed contrasting trends. Cluster 4, containing metabolites such as ethyl caffeate (Supplementary Table S1), declined with increasing grade. Clusters 5 and 6 exhibited a “bell” trend, with their metabolite levels peaking in the mid-range grades, indicating that the changes in the compounds in brandy could be closely related to its physical and chemical reactions. These findings suggest that grade-dependent changes in metabolites are associated with brandy quality.

Furthermore, integrating these metabolic profiles with sensory analysis revealed significant correlations between chemical composition and perceived sensory attributes. The increased composition included some fatty acids, phenolic acids and monosaccharides, which benefitted from the transfer of components from the oak barrels or the production of new substances during the ageing process to facilitate an increase in the concentration of non-volatile compounds in brandy (Roullier-Gall et al., 2020; Winstel et al., 2021), and the enrichment of these compounds in higher grades should be partly align with the sensory findings of enhanced smoothness sourness and sweetness, as observed in Section 3.1. Conversely, the decline in ethyl caffeate in premium grades was observed alongside reduced astringency and bitterness, which may suggest that grade-dependent changes in phenolic metabolites, including ethyl caffeate, are associated with the sensorial quality of brandies, although further studies are needed to establish causal links. This highlights the potential role of phenolic metabolites derived from both grapes and oak barrels in shaping the chemical and sensory profiles of brandies.

### 3.3 Metabolomic discriminators of brandy grades

**Fig. 4:**
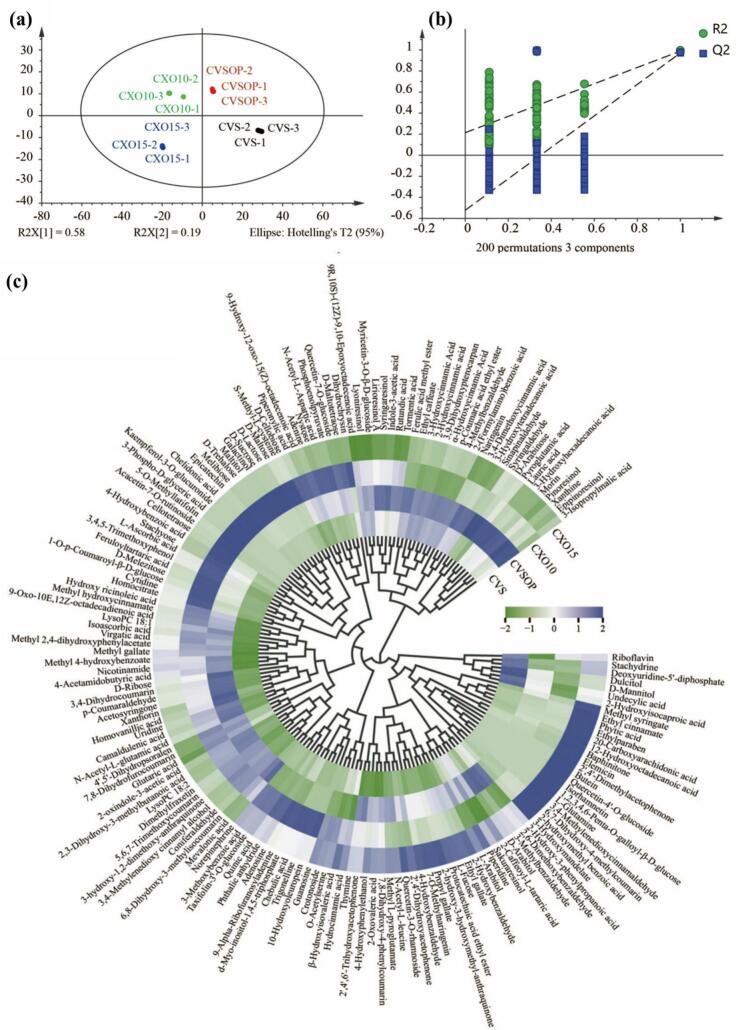
Identification of grade-specific metabolic markers using multivariate analysis. (a) Partial least squares-discriminant analysis (PLS-DA) score plot of metabolite profiles across four brandy grades (n = 3 per grade). The model parameters are R^2^X = 89.8%, R^2^Y = 99.6%, and Q^2^ = 98.5%. Each point represents an individual sample. (b) Validation of PLS-DA: a permutation test was conducted with 200 randomly initiated permutations in a PLS-DA model showing R^2^ (green rounds) and Q^2^ (blue rounds) values from the permuted analysis. (c) Circular heatmap visualizing the Z-score-normalized abundance of 164 grade-differential metabolites (VIP > 1, *p* < 0.05). The colour scale ranges from green (low abundance) to blue (high abundance). (For interpretation of the references to colour in this figure legend, the reader is referred to the web version of this article.)

To identify the key metabolomic markers distinguishing different brandy grades, a PLS-DA model was constructed using the metabolic dataset (Fig. 4a). The robustness of the model was validated by parameters including *R*^*2*^*X* = 89.8%, *R*^*2*^*Y* = 99.6%, and *Q*^*2*^ = 98.5, all exceeding the threshold of 0.5, confirming the model's capability to explain and predict grade-specific compositions. Permutation testing (*n* = 200) further validated the model's reliability, as *R*^*2*^ remained consistently above *Q*^*2*^, and the *Q*^*2*^ curve exhibited a negative start (Fig. 4b), indicative of its predictive power.

From the PLS-DA analysis, 164 metabolites with VIP > 1 and *p* < 0.05 were identified as grade-specific differentiators. These were predominantly phenolic compounds (75), followed by saccharides and their derivatives (22), and organic acids and their derivatives (13). A circular heatmap (Fig. 4c) provided a visual representation of the distribution and relative abundances of these metabolites across brandy grades, underscoring their grade-dependent variations. Regarding multivariate analysis, while permutation testing (n = 200) confirmed the validity of the PLS-DA model, external test set validation was not performed due to the limited sample size. Future studies with larger sample cohorts are needed to validate the identified grade-specific metabolic markers.

The metabolic profiles revealed distinct compositional signatures for each grade. In CVS, the most abundant compounds were dulcitol, d-mannitol, undecylic acid, and 2-hydroxyisocaproic acid, reflecting early-stage metabolic characteristics. In CVSOP, high concentrations of syringaldehyde, *p*-coumaraldehyde, sinapinaldehyde, and coniferaldehyde were observed, consistent with findings on other phenolic oxidation products (Cernîsev, 2017). CXO10 exhibited elevated levels of oligosaccharides (e.g., sucrose, maltose, and nystose), likely attributed to caramel addition. Notably, CXO15 contained large amounts of phenolic esters, including ethyl derivatives of gallic, cinnamic, and protocatechuic acids, indicative of advanced ageing and oak barrel interactions.

Many of these differentiators were sensory-related compounds, such as phenolic aldehydes (impacting astringency and bitterness) and oligosaccharides (contributing sweetness and viscosity), which align with radar chart findings (Section 3.1) and metabolite enrichment patterns (Section 3.2.2). This comprehensive metabolomic approach provides a robust framework for defining grade-specific quality attributes, offering insights into the chemical basis of sensory differences and potential biomarkers for brandy authentication and grading.

### 3.4 Relationship between the chemical composition of brandy and its oral sensory properties

**Fig. 5:**
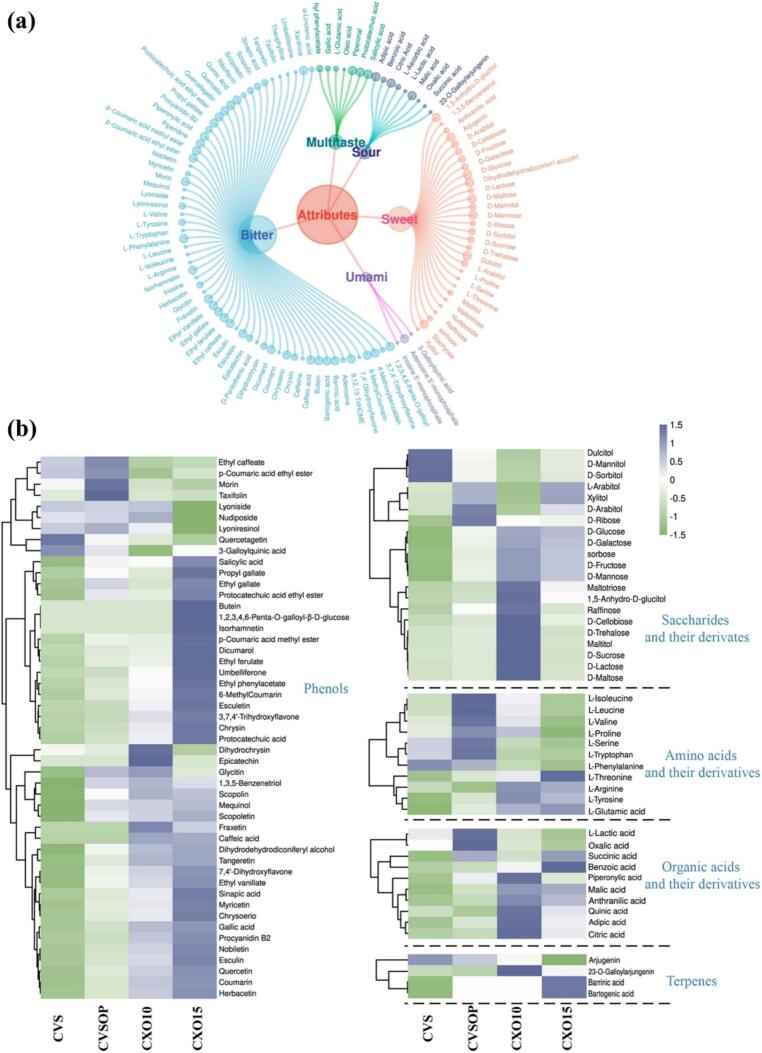
Relationship between the chemical composition and oral sensory properties of brandy. (a) Dendrogram and chord diagram illustrating the classification of 112 taste-active compounds identified in brandy based on their reported sensory properties according to the ChemTastesDB database (Rojas et al., 2022). (b) Clustered heatmap showing the Z-score-normalized abundance of the 112 taste-active compounds across four brandy grades. Hierarchical clustering (Euclidean distance method) was applied to both rows (compounds) and columns (samples). Columns represent individual samples (n = 3 per grade), and rows represent individual compounds. The colour scale ranges from green (low abundance) to blue (high abundance). (For interpretation of the references to colour in this figure legend, the reader is referred to the web version of this article.)

Brandy, characterized by its complex bittersweet profile, derives its sensorial properties from a myriad of chemical compounds. This study identified and emphasized 112 metabolites associated with flavor, utilizing the ChemTastesDB database (Rojas et al., 2022). These compounds—including key taste activators such as phenolic compounds, amino acids and their derivatives—were further linked to in-mouth sensory characteristics (e.g. astringency, bitterness) through multivariate analysis, as visualized in Fig. 5. Specifically, Fig. 5a illustrates the chemical compounds linked to oral sensory attributes, while Fig. 5b reveals the clustered relationships of standardized potential taste activators across four brandy grades. Several methodological considerations should be noted when interpreting these associations. First, the metabolomics data reported are semi-quantitative (relative peak area ratios normalized to internal standard) rather than absolute concentrations, as calibration curves, LOD, LOQ, and recovery rates were not determined for individual metabolites. Second, regarding compound identification, only 137 metabolites (20%) were identified at Level 1, and the remaining metabolites were putatively identified at Level 2 (20%) or Level 3 (60%). In particular, coumarins, lignans, and triterpenes—key compound classes discussed below—were predominantly identified at Level 2 or Level 3. Consequently, the associations discussed represent potential contributions based on relative abundance patterns and literature-reported taste properties, rather than established causal relationships. The result (Fig. 5a) showed that over half of these compounds contribute to bitterness, while the remainder impart sweetness, sourness, or a combination of these tastes. The following discussion focuses on five key compound categories: phenolic compounds, terpenes, amino acids and their derivatives, derivatives of saccharides, and organic acids, elucidating their roles in shaping the sensory experience of brandy.

#### 3.4.1 Phenolic compounds

Phenolic compounds are a diverse group of aromatic compounds that play a central role in shaping the sensory profile of grape-derived and oak-aged alcoholic beverages, including brandy. In this study, a total of 49 phenolic taste molecules were identified in brandy (Fig. 5b), belonging to four main categories: phenolic acids and their derivatives, coumarins, flavonoids, and lignans. These compounds contribute significantly to the complex flavor profile of brandy, particularly its bitterness, astringency and sourness (Huang & Xu, 2021; Soares et al., 2013).

Among the phenolic compounds detected, phenolic acids and their derivatives were the most abundant contributors to taste. Eighteen compounds, including phenolic acids and phenolic esters (e.g., protocatechuic acid and gallic acid), were identified as bitter or sour compounds. Their concentrations increased with brandy grade, consistent with previous findings (Canas, Casanova, & Belchior, 2008; Schwarz, Rodríguez, Guillén, & Barroso, 2011). Notably, phenolic esters such as protocatechuic acid ethyl ester and ethyl ferulate were found in higher concentrations in premium grades, particularly CXO15. These esters have been previously shown to contribute bitterness to wines (Hufnagel & Hofmann, 2008), suggesting a similar role in brandy.

Coumarins, a class of lactones derived from ortho-hydroxycinnamic acid, were also significant contributors to brandy's bitterness. A total of 28 coumarins were detected, with 9 being bitter compounds. Among these, esculetin, umbelliferone, coumarin, fraxetin and scopoletin exhibited concentrations ranging from a few tens to 400 μg/L, with their bitterness potentially interacting perceptually to enhance overall sensory impact (Winstel et al., 2020). Four additional bitter coumarins—dicumarol, 6-methylcoumarin, esculin, and scopoline—were identified for the first time in brandy, with their concentrations increasing as grade quality improved. This finding aligns with the role of coumarins in contributing bitterness to other alcoholic beverages (Oliveira-Alves, Lourenço, Fernandes, & Canas, 2024).

Flavonoids, derived directly from oak heartwood, were identified as minor contributors to brandy's gustatory properties. While 18 bitter compounds such as isorhamnetin, quercetagetin, morin, taxifolin and epicatechin were detected, their absolute concentrations have been reported to be low (Canas, 2017). This suggests that flavonoids primarily contribute to the overall complexity of brandy rather than dominating its sensory attributes.

Lignans, a class of natural polymers formed from phenylpropanoid derivatives, exhibited distinct sensory contributions. Among the 24 lignans detected, lyoniresinol and lyoniside were the most abundant bitter compounds, particularly in lower grades of brandy. Studies have shown that Lyoniresinol concentrations often exceed sensory thresholds, making it a key driver of bitterness in brandies (Sindt, Gammacurta, Waffo-Teguo, Dubourdieu, & Marchal, 2016; Winstel et al., 2019). In contrast, dihydrodehydrodiconiferyl alcohol, a sweet lignan, was most abundant in premium brands and contributed sweetness while reducing ethanol burn at concentrations around 2 mg/L (Winstel et al., 2021).

Collectively, these findings highlight the sensory contributions of phenolic subcategories, with phenolic acids, coumarins, and lignans playing dominant roles in shaping brandy's bitterness and sweetness. The interplay between these compounds likely enhances sensory perception, as observed in other beverages like red wine and tea (Y. H. Chen et al., 2022; Ferrer-Gallego, Hernández-Hierro, Rivas-Gonzalo, & Escribano-Bailón, 2014). However, further research is needed to explore potential synergistic effects among phenolic subcategories and their impact on brandy's sensory profile.

#### 3.4.2 Saccharides and their derivatives

Saccharides and their derivatives are formed through the degradation of oak hemicellulose and the addition of caramel coloring. These compounds are pivotal in imparting the sweetness of brandy and have been observed to accumulate progressively over time, reaching concentrations as high as 2 g/L after 40 years of ageing (Montero et al., 2005; Viriot, Scalbert, Lapierre, & Moutounet, 1993). In this study, 22 sweet compounds were identified among saccharides (Fig. 5a), comprising nine oligosaccharides, six monosaccharides, and seven monosaccharide alcohols. Notably, seven of the sweet oligosaccharides exhibited grade-dependent variations, with their concentrations peaking in the CXO10 grade (Fig. 5b). Among these, maltose and sucrose showed particularly pronounced trends. The contents of sweet monosaccharides, including glucose, fructose, galactose, sorbose, and mannose, increased consistently with brandy grade, reaching their highest levels in the CXO15 grade. These findings are consistent with previous studies (Gomis, Tamayo, & Alonso, 2003; Montero et al., 2005), suggesting that these sweet sugars play a significant role in promoting the perception of sweetness in brandy.

#### 3.4.3 Amino acids and their derivatives

The composition and abundance of amino acids and their derivatives play a crucial role in determining the quality of brandy. We acknowledge that brandy is a distilled spirit, and amino acids are non-volatile components that are not expected to carry over during distillation. However, trace amounts may be introduced through post-distillation additions such as commercial oak extract and caramel colour, which can contribute amino acids or melanoidin degradation products; and entrainment during pot still distillation, which can carry over trace amounts of non-volatile components. While previous studies have predominantly focused on the aroma effects of amino acids, particularly through processes like deamination and decarboxylation leading to the formation of aromatic aldehydes, alcohols, and acetals (Tsakiris et al., 2014; Uselmann & Schieberle, 2015), the impact of these compounds on the gustatory characteristics of brandy remains underexplored. In this study, we identified 29 compounds (Fig. 5a), including free amino acids that contribute to various taste sensations. Aromatic amino acids such as L-proline, L-threonine, and l-serine impart sweetness, while L-glutamic acid and L-aspartic acid are responsible for umami and acidity. Conversely, L-arginine, *L*-phenylalanine, and L-tyrosine are associated with bitterness, a critical attribute in stronger spirits like brandy.

The behavior of these amino acids varies across different grades of brandy (Fig. 5b). Notably, certain amino acids, including L-arginine, L-tyrosine, L-threonine, and l-glutamine, exhibit an increase in concentration with higher grades. Meanwhile, others, such as *L*-phenylalanine, L-tryptophan, and l-serine, show a decrease. Additionally, there are amino acids like L-isoleucine, L-leucine, L-valine, and L-proline, whose concentrations peak at specific grades, particularly at the CVSOP level. However, the taste thresholds for these amino acids, typically exceeding several hundred mg/L (Schiffman, Sennewald, & Gagnon, 1981), suggest that their perceived impact on taste may be limited at the concentrations found in brandy. Interestingly, studies indicate that at concentrations as high as 3 g/L, L-proline can enhance sweetness and reduce bitterness and astringency in wine (Nandorfy et al., 2022). In conclusion, while amino acids significantly contribute to the aroma of brandy, their impact on taste appears minimal at the concentrations observed in this study. This suggests that further research is necessary to fully understand the role of these compounds in shaping the sensory profile of brandy, particularly at lower concentrations.

#### 3.4.4 Organic acids and their derivatives

Organic acids and their derivatives are present in brandy and play a significant role in its sensory profile. Specifically, ten organic acids have been associated with taste characteristics (Fig. 5a, b): succinic, benzoic, malic, lactic, citric, adipic, and oxalic acids contribute to sourness, while quinic and piperonylic acids are linked to bitterness. A notable bell-shaped pattern was observed in the variation of lactic and oxalic acids—the major organic acids in brandy—as the quality grade of the spirit increased, with maximum levels achieved in CVSOP grade brandy (Park, Kim, & Kim, 1999). Furthermore, the remaining organic acids that contribute to the flavor of brandy are more prominent in XO grade, which has undergone prolonged ageing. This is attributed to the extraction of these acids from barrel wood during the ageing process and the evaporation of ethanol and water (Conner, Paterson, Birkmyre, & Piggott, 1999). Consistent with previous studies (Schwarz et al., 2011), the results indicate that prolonged ageing increases the concentration of organic acids in brandy. These findings reinforce the critical role of organic acids in the sourness of alcoholic beverages (Hufnagel et al., 2008), suggesting that achieving the characteristic sourness of brandy likely depends on the presence and concentration of organic acids.

#### 3.4.5 Terpenes

Terpenes play a significant role in both the aroma and taste of brandy. This study identified four key compounds: 23-*O*-galloylarjungenin, arjugenin, barrinic acid, and bartogenic acid. These compounds exhibit distinct trends depending on the grade of the spirit. Specifically, 23-*O*-galloylarjungenin and arjugenin, which contribute sweetness as oak-derived quercotriterpenoside analogues, showed decreasing concentrations with increasing brandy grade. In addition, nine other quercotriterpenoside derivatives with sweetness perceived at 5 mg/L have been identified in brandy (Gammacurta et al., 2019). On the other hand, the levels of barrinic and bartogenic acids, which induce bitterness, increased with higher brandy grade. Previous research has shown that the bitterness of these isomers becomes noticeable at the same concentration of 5 mg/L (Gammacurta et al., 2020). Despite these findings, the absolute quantities and taste thresholds of these terpenes in brandy remain undetermined. Further investigation is necessary to fully understand their true contribution to the sensory profile of brandy.

#### 3.4.6 Perception of brandy

The study investigates the intricate flavor profiles of brandy, focusing on the compounds responsible for sweetness, sourness, and bitterness. Premium brandies, characterized by extended ageing, exhibit distinct sweet and sour attributes. The sweetness in these premium spirits can be attributed to phenolic compounds and saccharides, while sourness is primarily caused by phenolic acids and organic acids. In contrast, lower-grade brandies display heightened bitterness, likely influenced by phenolic acids, coumarins, and lignans. Despite the low levels of certain bitter compounds in the CVS grade, the highest bitterness scores were observed in this grade. One possible explanation, which remains to be tested, involves perceptual interactions between sweet and bitter compounds. Previous research has demonstrated that sweet compounds can suppress bitter perception (Keast & Breslin, 2003). In lower-grade brandies, the relative deficiency of sweet compounds (saccharides and sweet phenolics) may result in reduced suppression of bitter signals, allowing even modest concentrations of bitter compounds to be perceived more intensely. Additionally, additive or synergistic interactions among multiple bitter compounds—even at sub-threshold concentrations—could collectively exceed the bitterness detection threshold (Keast et al., 2003). Notably, bitter ellagitannins have been found to be prevalent in brandies aged for a short period (Gadrat, Lavergne, Emo, Teissedre, & Chira, 2021) and may contribute to the perception of bitterness in lower-grade spirits. However, bitter ellagitannins were not detected in our study, possibly due to their structural instability and similarity, which necessitate specialized analytical techniques for accurate identification. This investigation underscores the complexity of flavor profiles in brandy and highlights the need for advanced analytical methods to fully understand the role of each compound, thereby enhancing sensory quality. In this context, it is important to recognize the limitations of the current data. As the compound-sensory relationships in this study are based on semi-quantitative relative abundance data, future studies employing absolute quantification methods (e.g., calibration curves with authentic standards, stable isotope-labeled internal standards) will be essential to establish precise concentration-sensory response relationships and definitively determine which compounds contribute to sensory perception at their actual concentrations in brandy. Beyond absolute quantification, the underlying perceptual mechanisms also require validation. It is important to note that the proposed perceptual interactions are hypothesis-generating based on our correlative data; confirmation requires future sensory studies using omission tests and recombination models (e.g., systematic addition or removal of candidate compounds in model brandy solutions) to directly validate the synergistic and masking effects suggested here.

## 4 Conclusion

This study systematically investigated the sensory characteristics, non-volatile metabolite profiles, and interrelationships across different grades of brandy using descriptive sensory analysis and widely targeted metabolomics. Higher grades exhibited enhanced sweetness, sourness, and smoothness, while lower grades were characterized by higher alcohol burn, astringency, and bitterness, with strong correlations between sensory attributes and grade (|r| > 0.8). Non-targeted metabolomics identified 682 non-volatile compounds, with phenolic compounds (e.g., phenolic acids, coumarins, and lignans) and saccharides dominating the profiles. Multivariate analyses revealed distinct metabolic signatures across grades, and PLS-DA identified 164 grade-specific metabolites. A total of 112 metabolites associated with intra-oral perception were identified, with phenolic compounds linked to bitterness, saccharides and certain phenols contributing to sweetness, and organic acids associated with sourness. This study suggests that grade-dependent changes in metabolites are associated with brandy quality. The integration of sensory analysis with metabolomics offers a systematic understanding of how maturation influences brandy quality, providing potential biomarkers for quality control and advancing the field of flavor chemistry. Future studies employing optimized extraction protocols and absolute quantification methods will be essential to further strengthen the characterization of brandy's chemical-sensory relationships by calculating dose-over-threshold (DoT) factors and performing sensory reconstitution experiments to validate their actual sensory contributions.

## CRediT authorship contribution statement

**Yue Ma:** Writing – original draft, Visualization, Validation, Methodology, Formal analysis, Data curation. **Yuhao Liu:** Writing – review & editing, Validation, Investigation, Formal analysis, Data curation, Conceptualization. **Baochun Zhang:** Supervision, Resources, Project administration, Conceptualization. **Chunhua Shen:** Resources, Methodology. **Lina Yu:** Resources, Methodology. **Yan Xu:** Writing – review & editing, Supervision, Project administration. **Ke Tang:** Writing – review & editing, Supervision, Resources, Project administration, Methodology, Funding acquisition, Conceptualization.

## Declaration of competing interest

The authors declare that they have no known competing financial interests or personal relationships that could have appeared to influence the work reported in this paper.

## Data Availability

Data will be made available on request.
