# Supplementary material for: Determination of non-volatile metabolic profiles and their sensory relevance in different grades of brandy through widely targeted metabolomics
Source: Food Chem X. 2026 Apr 21;36:103889. doi: 10.1016/j.fochx.2026.103889 (PMC13129455; doi:10.1016/j.fochx.2026.103889)
Supplement: Supplementary material [file mmc1.docx]

Table S1 Non-volatile compounds of different grades of brandy

| Number | Formula | Compounds | Class Ⅰ | Class Ⅱ | Level | CVS | CVSOP | CXO10 | CXO15 | Tasteness | Mfuzz |
| --- | --- | --- | --- | --- | --- | --- | --- | --- | --- | --- | --- |
| 1 | C4H9NO3 | L-Threonine | Amino acids and their derivatives | | 3 | 12.18±2.91 | 20.1±10.55 | 27.05±1.85 | 51.24±9.89 | Sweetness |  |
| 2 | C11H12N2O2 | L-Tryptophan | Amino acids and their derivatives | | 3 | 50.65±16.5 | 55.86±4.27 | 41.99±7.05 | 51.3±1.41 | Bitterness |  |
| 3 | C4H8N2O3 | L-Asparagine | Amino acids and their derivatives | | 3 | 4.6±3.44 | 0±0 | 0±0 | 6.85±1.11 |  |  |
| 4 | C9H11NO3 | L-Tyrosine | Amino acids and their derivatives | | 3 | 201.21±18.35 | 362.11±48.17 | 665.74±57.7 | 715.24±37.98 | Bitterness | 3 |
| 5 | C6H13NO2 | L-Leucine | Amino acids and their derivatives | | 2 | 757.83±28.64 | 1352.68±187.93 | 971.14±163.91 | 790.67±22.06 | Bitterness |  |
| 6 | C6H13NO2 | L-Isoleucine | Amino acids and their derivatives | | 2 | 764.53±59.61 | 1349.76±166.68 | 957.16±184.37 | 793.38±18.15 | Bitterness |  |
| 7 | C9H11NO2 | L-Phenylalanine | Amino acids and their derivatives | | 3 | 2530.78±220.74 | 2268.48±115.51 | 1202.67±106.5 | 1128.71±45.7 | Bitterness | 4 |
| 8 | C5H9NO4 | L-Glutamic acid | Amino acids and their derivatives | | 3 | 38.82±16.07 | 70.11±10.99 | 103.4±7.25 | 139.59±9.77 | Multitaste | 3 |
| 9 | C5H9NO2 | L-Proline | Amino acids and their derivatives | | 3 | 3090.09±1376.41 | 5726.49±1045.54 | 4727.55±499.81 | 1436.01±480.63 | Sweetness |  |
| 10 | C5H11NO2 | L-Valine | Amino acids and their derivatives | | 3 | 186.06±18.37 | 271.89±19.72 | 213.99±41.8 | 183.2±9.03 | Bitterness |  |
| 11 | C6H14N4O2 | L-Arginine | Amino acids and their derivatives | | 3 | 149.43±12.45 | 143.75±4.27 | 165.22±22.85 | 203.14±16.6 | Bitterness |  |
| 12 | C5H10N2O3 | L-Glutamine | Amino acids and their derivatives | | 3 | 0±0 | 0±0 | 0±0 | 21.45±4.92 |  | 1 |
| 13 | C7H15N3O3 | L-Homocitrulline | Amino acids and their derivatives | | 3 | 87.56±9.05 | 160.1±2.13 | 204.88±25.87 | 243.92±20.73 |  | 3 |
| 14 | C3H7NO3 | L-Serine | Amino acids and their derivatives | | 3 | 56.3±23.19 | 69.09±26.33 | 37.54±5.39 | 38.83±6.95 | Sweetness |  |
| 15 | C6H9NO5 | N-Acetyl-L-Aspartic Acid | Amino acids and their derivatives | | 3 | 0±0 | 8±1.49 | 16.42±2.52 | 0±0 |  | 5 |
| 16 | C4H9NO3 | L-Homoserine | Amino acids and their derivatives | | 3 | 10.15±6.7 | 22.5±4.33 | 26.05±2.78 | 46.41±6.85 |  |  |
| 17 | C11H13NO3 | N-Acetyl-DL-phenylalanine | Amino acids and their derivatives | | 3 | 214.41±18.63 | 363.7±31.32 | 492.45±93.75 | 580.96±14.81 |  |  |
| 18 | C6H11NO3 | N-Methyl-Trans-4-Hydroxy-L-Proline | Amino acids and their derivatives | | 3 | 180.09±79.84 | 160.22±18.11 | 378.55±142.02 | 600.6±273.52 |  |  |
| 19 | C7H11NO5 | N-Acetyl-L-glutamic acid | Amino acids and their derivatives | | 3 | 0±0 | 16.86±2.02 | 10.77±0.26 | 7.22±0.39 |  | 6 |
| 20 | C8H15NO3 | N-Acetyl-L-leucine | Amino acids and their derivatives | | 3 | 197.17±18.02 | 682.74±37.19 | 284.19±31.38 | 862.08±15.97 |  | 1 |
| 21 | C6H11NO2 | N-Methyl-L-proline | Amino acids and their derivatives | | 3 | 2802.39±135.63 | 7036.47±361.5 | 8907.89±300.95 | 12898.52±637.39 |  | 2 |
| 22 | C5H9NO4 | O-Acetylserine | Amino acids and their derivatives | | 3 | 51.75±6.56 | 0±0 | 111.67±24.22 | 130.19±15.96 |  | 3 |
| 23 | C5H7NO3 | Pyroglutamic acid* | Amino acids and their derivatives | | 1 | 74.52±2.57 | 200.64±5.16 | 112.85±14.63 | 133.12±5.55 |  | 6 |
| 24 | C10H13NO2 | N-methylphenylalanine | Amino acids and their derivatives | | 3 | 30.54±2.27 | 42.95±8.07 | 65.57±10.76 | 180.96±18.33 |  | 1 |
| 25 | C10H11NO3 | N-Phenylacetylglycine | Amino acids and their derivatives | | 3 | 125.9±8.21 | 257.07±7.53 | 290.12±52.6 | 216.44±11.19 |  |  |
| 26 | C7H11N3O2 | 3-Methyl-L-Histidine | Amino acids and their derivatives | | 3 | 55.69±0.53 | 73.66±4.06 | 141.24±9.68 | 233.28±15.45 |  | 2 |
| 27 | C6H10O5 | 3-Hydroxy-3-methylpentane-1,5-dioic acid* | Amino acids and their derivatives | | 1 | 598±5.22 | 1098.7±44.5 | 1868.07±378.39 | 2348.64±104.26 |  | 3 |
| 28 | C11H22N2O3 | L-Valyl-L-Leucine | Amino acids and their derivatives | | 2 | 150.07±36.74 | 141.02±5.47 | 247.59±25.14 | 273.94±16.77 |  |  |
| 29 | C4H9NO2S | S-Methyl-L-cysteine | Amino acids and their derivatives | | 2 | 184.01±13.74 | 252.71±27.35 | 843.49±94.88 | 370.68±19.15 |  | 5 |
| 30 | C20H22O9 | Piceatannol-3'-O-glucoside | Phenols | Stilbenes | 3 | 0±0 | 0±0 | 376.4±67.94 | 808.91±66.19 |  | 2 |
| 31 | C42H32O9 | ampelopsin C | Phenols | Stilbenes | 3 | 2462.32±315.54 | 3005.63±169.24 | 3865.23±429.68 | 8259.29±611.18 |  | 1 |
| 32 | C14H12O4 | Piceatannol | Phenols | Stilbenes | 3 | 77.34±10.56 | 86.2±18.64 | 86.96±12.75 | 121.16±18.25 |  |  |
| 33 | C28H22O6 | epsilon-Viniferin | Phenols | Stilbenes | 3 | 56.04±14 | 88.31±4.94 | 105.56±10.37 | 153.08±29.07 |  |  |
| 34 | C15H14O4 | Rhapontigenin | Phenols | Stilbenes | 3 | 18.9±1.89 | 29.18±3.36 | 29.9±2.77 | 36.66±4.44 |  |  |
| 35 | C15H14O4 | Isorhapontigenin | Phenols | Stilbenes | 3 | 18.17±2.92 | 26.36±2.58 | 28.4±0.82 | 38.53±3.45 |  |  |
| 36 | C16H18O9 | Cryptochlorogenic acid (4-O-Caffeoylquinic acid) | Phenols | Phenolic acid and their derivatives | 2 | 668.7±39.22 | 727.49±41.02 | 611±228.03 | 0±0 |  |  |
| 37 | C7H6O3 | 4-Hydroxybenzoic acid | Phenols | Phenolic acid and their derivatives | 2 | 0±0 | 229.99±33.56 | 1231.41±326.05 | 0±0 |  | 5 |
| 38 | C12H14O4 | Ethyl ferulate* | Phenols | Phenolic acid and their derivatives | 1 | 710.73±18.51 | 1346.4±40.78 | 1492.86±23.76 | 4392.4±83.69 | Bitterness | 1 |
| 39 | C20H20O14 | 1,6-bis-O-galloyl-beta-D-glucose | Phenols | Phenolic acid and their derivatives | 1 | 1540.44±116.16 | 2289.49±19.79 | 2242.59±166.64 | 4500.83±142.6 |  | 1 |
| 40 | C9H8O4 | Caffeic acid | Phenols | Phenolic acid and their derivatives | 3 | 0±0 | 0±0 | 3839.52±138.66 | 4612.19±197.11 | Bitterness | 3 |
| 41 | C19H14O12 | Ellagic acid-4-O-Xyloside | Phenols | Phenolic acid and their derivatives | 2 | 851.81±23.41 | 1368.31±40.12 | 1775.1±447.79 | 4645.82±263.12 |  | 1 |
| 42 | C7H6O3 | 2,5-Dihydroxybenzaldehyde | Phenols | Phenolic acid and their derivatives | 2 | 1275.16±39.05 | 2184.32±18.56 | 2346.56±207.22 | 4734.36±166.05 |  | 1 |
| 43 | C8H8O3 | 2-Hydroxy-4-Methoxybenzaldehyde | Phenols | Phenolic acid and their derivatives | 3 | 1156.01±403.17 | 2011.55±737.68 | 2307.98±47.42 | 4857.13±605.53 |  |  |
| 44 | C15H18O8 | 1-O-p-Coumaroyl-β-D-glucose | Phenols | Phenolic acid and their derivatives | 3 | 159.2±6.11 | 408.16±10.22 | 990.26±43.45 | 485.81±28.76 |  | 5 |
| 45 | C11H14O4 | Sinapyl alcohol | Phenols | Phenolic acid and their derivatives | 3 | 398.05±34.01 | 544.64±60.62 | 258.2±42.87 | 492.13±147.42 |  |  |
| 46 | C8H8O4 | Protocatechuic Acid Methyl Ester | Phenols | Phenolic acid and their derivatives | 2 | 133.07±16.12 | 255.24±35.4 | 408.8±40.16 | 496.73±14.83 |  | 3 |
| 47 | C14H6O8 | Ellagic acid* | Phenols | Phenolic acid and their derivatives | 1 | 87.59±1.96 | 216.57±15.92 | 308.41±70.85 | 513.02±29.53 |  | 2 |
| 48 | C13H14O4 | Aloesol | Phenols | Phenolic acid and their derivatives | 2 | 268.29±7.73 | 500.27±14.28 | 516.53±90.4 | 522.25±14.06 |  |  |
| 49 | C7H6O3 | Protocatechualdehyde | Phenols | Phenolic acid and their derivatives | 2 | 1043.16±15.57 | 1972.59±23.91 | 2630.04±374.62 | 5301.03±236.59 |  | 1 |
| 50 | C13H18O8 | Isotachioside | Phenols | Phenolic acid and their derivatives | 3 | 0±0 | 0±0 | 917.87±188.65 | 538.96±69.22 |  | 5 |
| 51 | C14H20N2O3 | N-Feruloylputrescine | Phenols | Phenolic acid and their derivatives | 3 | 203.24±115.44 | 328.72±8.3 | 489.91±46.76 | 547.76±20.39 |  |  |
| 52 | C7H6O3 | Salicylic acid | Phenols | Phenolic acid and their derivatives | 2 | 1315.91±23.04 | 3092.1±87.98 | 2972.57±272.76 | 5485.82±116.46 | Multitaste | 2 |
| 53 | C9H10O3 | Ethylparaben | Phenols | Phenolic acid and their derivatives | 1 | 610.66±52.08 | 974.59±23.7 | 987.75±213.27 | 5526.48±5.45 |  | 1 |
| 54 | C10H10O2 | Methyl Cinnamate | Phenols | Phenolic acid and their derivatives | 3 | 1435.36±74.26 | 2405.7±26.74 | 2905.88±241.61 | 5545.54±458.26 |  | 1 |
| 55 | C9H10O4 | Protocatechuic acid ethyl ester* | Phenols | Phenolic acid and their derivatives | 1 | 2086.41±65.03 | 3503.18±68.73 | 2903.25±125.44 | 5615.63±220.38 | Bitterness | 1 |
| 56 | C9H10O2 | Hydrocinnamic acid | Phenols | Phenolic acid and their derivatives | 3 | 30.22±3.39 | 0±0 | 38.13±2.9 | 56.56±0.33 |  | 1 |
| 57 | C7H8O2 | Mequinol | Phenols | Phenolic acid and their derivatives | 2 | 251.55±12.65 | 436.71±76.43 | 439.31±37.36 | 569.78±74.03 | Bitterness |  |
| 58 | C16H14O9 | 3-O-(3-O-Methylgalloyl) Methylgallate | Phenols | Phenolic acid and their derivatives | 2 | 37.64±1.42 | 63.72±9.09 | 51.08±2.76 | 60.77±4.95 |  |  |
| 59 | C16H20O9 | 1-O-Feruloyl-β-D-glucose | Phenols | Phenolic acid and their derivatives | 3 | 0±0 | 0±0 | 327.06±45.96 | 616.64±17.06 |  | 2 |
| 60 | C7H6O4 | 2,3-Dihydroxybenzoic Acid* | Phenols | Phenolic acid and their derivatives | 1 | 207.58±14.68 | 256.8±9.35 | 323.1±21 | 621.61±21.49 |  | 1 |
| 61 | C11H14O4 | Methyl 3-(3-hydroxy-4-methoxyphenyl)propanoate | Phenols | Phenolic acid and their derivatives | 3 | 412.92±44.05 | 574.74±64.86 | 234.81±38.07 | 659.81±390.49 |  |  |
| 62 | C8H10O3 | Hydroxytyrosol | Phenols | Phenolic acid and their derivatives | 2 | 1392.49±132.11 | 1848.61±46.23 | 2818.56±191.22 | 6638.53±165.6 |  | 1 |
| 63 | C16H22O7 | Eugenol Glucoside | Phenols | Phenolic acid and their derivatives | 3 | 0±0 | 29.09±11.05 | 54±19.08 | 67.94±3.78 |  |  |
| 64 | C27H24O18 | 1,4,6-Tri-O-galloyl-β-D-glucose | Phenols | Phenolic acid and their derivatives | 1 | 222.3±6.6 | 245.57±8.38 | 307.14±30.14 | 703.25±58.56 |  | 1 |
| 65 | C8H8O4 | Isovanillic Acid* | Phenols | Phenolic acid and their derivatives | 1 | 1620.89±118.31 | 2438.27±180.45 | 3904.85±840.55 | 7035.1±1380.25 |  |  |
| 66 | C14H10O8 | Methyl Brevifolincarboxylate | Phenols | Phenolic acid and their derivatives | 3 | 577.62±3.12 | 583.81±40.21 | 528.03±93.73 | 712.04±22.43 |  |  |
| 67 | C8H8O3 | 3-Methoxybenzoic acid | Phenols | Phenolic acid and their derivatives | 3 | 13.26±2.1 | 0±0 | 79.47±8.67 | 71.94±0.18 |  | 3 |
| 68 | C10H12O4 | Ethyl vanillate* | Phenols | Phenolic acid and their derivatives | 1 | 1464.68±68.08 | 3473.44±92.96 | 4495.83±326.78 | 7357.65±227.52 | Bitterness | 2 |
| 69 | C27H24O18 | 2,4,6-Tri-O-galloyl-D-glucose* | Phenols | Phenolic acid and their derivatives | 1 | 321.35±92.34 | 343.47±136.01 | 314.39±61.07 | 739.44±22.24 |  |  |
| 70 | C15H18O9 | 1-O-Caffeoyl-β-D-glucose | Phenols | Phenolic acid and their derivatives | 3 | 0±0 | 0±0 | 553.78±49.31 | 742.9±174.64 |  | 3 |
| 71 | C11H12O2 | Ethyl cinnamate | Phenols | Phenolic acid and their derivatives | 2 | 141.05±6.02 | 165.73±19.9 | 191.77±48.88 | 761.41±66.99 |  | 1 |
| 72 | C9H10O5 | Ethyl gallate | Phenols | Phenolic acid and their derivatives | 1 | 342.56±15.59 | 530.44±15.33 | 408.47±11.23 | 773.01±13.9 | Bitterness | 1 |
| 73 | C15H20O8 | Androsin | Phenols | Phenolic acid and their derivatives | 3 | 18.73±5.79 | 0±0 | 0±0 | 79.56±27.73 |  |  |
| 74 | C9H10O5 | 4-Hydroxy-3-methoxymandelate | Phenols | Phenolic acid and their derivatives | 3 | 35.41±7.13 | 78.94±1.45 | 106.28±17.1 | 80.09±2.98 |  | 5 |
| 75 | C10H12O3 | Methyl-3-(3-hydroxyphenyl)Propionate | Phenols | Phenolic acid and their derivatives | 2 | 98.85±15.04 | 350.91±9.25 | 622.49±8.12 | 813.45±4.64 |  | 3 |
| 76 | C9H10O4 | Dihydrocaffeic acid | Phenols | Phenolic acid and their derivatives | 3 | 201.65±13.84 | 323.2±30.33 | 565.2±77.04 | 861.04±105.23 |  | 2 |
| 77 | C8H8O3 | Methyl 4-hydroxybenzoate | Phenols | Phenolic acid and their derivatives | 2 | 4226.25±281.72 | 8338.02±706 | 6919.19±668.58 | 8648.17±194.02 |  | 6 |
| 78 | C13H18O2 | 4-Hydroxy-3,5-diisopropylbenzaldehyde | Phenols | Phenolic acid and their derivatives | 3 | 83.38±4.82 | 71.93±2.43 | 53.23±11.8 | 89.84±8.85 |  |  |
| 79 | C8H8O3 | Isovanillin | Phenols | Phenolic acid and their derivatives | 1 | 341.79±13.99 | 633.15±43.37 | 682.84±24.51 | 907.52±2.2 |  | 3 |
| 80 | C8H8O3 | Vanillin | Phenols | Phenolic acid and their derivatives | 1 | 339.85±7.2 | 633.53±50.44 | 662.7±21.48 | 917.58±40.35 |  | 2 |
| 81 | C41H32O26 | 1,2,3,4,6-Penta-O-galloyl-β-D-glucose | Phenols | Phenolic acid and their derivatives | 3 | 0±0 | 0±0 | 0±0 | 9.24±0.57 | Bitterness | 1 |
| 82 | C14H20O9 | Leonuriside A | Phenols | Phenolic acid and their derivatives | 2 | 452.48±23.51 | 802.67±55.07 | 641.48±199.28 | 940.59±36.87 |  |  |
| 83 | C16H18O8 | 4-O-p-Coumaroylquinic acid | Phenols | Phenolic acid and their derivatives | 3 | 39.05±1.45 | 47.5±3.15 | 84.58±12.46 | 94.94±12.35 |  |  |
| 84 | C11H12O4 | Ethyl caffeate | Phenols | Phenolic acid and their derivatives | 1 | 1775.31±74.98 | 2302.65±5.19 | 621.12±72.12 | 960.78±2.96 | Bitterness | 4 |
| 85 | C10H12O2 | Ethyl phenylacetate | Phenols | Phenolic acid and their derivatives | 3 | 253.78±21.87 | 357.48±4.68 | 445.55±49.22 | 987.74±114.1 | Multitaste | 1 |
| 86 | C14H10O9 | Digallic Acid | Phenols | Phenolic acid and their derivatives | 1 | 127.17±8.06 | 374.61±8.1 | 524.89±36.21 | 1024.77±19.94 |  | 2 |
| 87 | C8H8O4 | 3-Hydroxymandelate | Phenols | Phenolic acid and their derivatives | 2 | 2088.83±106.35 | 967.98±33.82 | 1988.59±237.26 | 10257.81±309.37 |  | 1 |
| 88 | C8H8O3 | 4-Hydroxyphenylacetic acid | Phenols | Phenolic acid and their derivatives | 3 | 0±0 | 51.82±16.99 | 87.08±17.56 | 102.96±8.39 |  | 3 |
| 89 | C7H6O4 | Gentisic Acid | Phenols | Phenolic acid and their derivatives | 1 | 2728.15±123.43 | 3757.59±117.57 | 5470.3±820.86 | 10494.43±639.97 |  | 1 |
| 90 | C15H18O7 | 1-O-trans-Cinnamoyl-beta-D-glucopyranose | Phenols | Phenolic acid and their derivatives | 2 | 430.74±22.96 | 234.09±152.57 | 1511.64±445.49 | 1057.01±343.67 |  |  |
| 91 | C10H14O5 | Erythro-Guaiacylglycerol | Phenols | Phenolic acid and their derivatives | 2 | 24.13±0.75 | 45.72±2.22 | 71.82±10.88 | 106.08±6.47 |  | 2 |
| 92 | C9H8O | Cinnamaldehyde | Phenols | Phenolic acid and their derivatives | 3 | 664.03±82.21 | 695.91±39.46 | 788.55±130.45 | 1069.2±81.34 |  |  |
| 93 | C14H18O8 | 2-Methoxycarbonylphenyl b-D-glucopyranoside | Phenols | Phenolic acid and their derivatives | 3 | 404.18±5.62 | 585.48±9.51 | 806.39±53.51 | 1074.24±497.12 |  |  |
| 94 | C10H10O3 | Coniferaldehyde* | Phenols | Phenolic acid and their derivatives | 1 | 7041.92±315.54 | 13202.25±271.85 | 13106.54±1409.54 | 10773.23±76.55 |  | 6 |
| 95 | C7H6O4 | Methyl cumalate | Phenols | Phenolic acid and their derivatives | 1 | 2930.73±95.78 | 3900.68±95.09 | 5471.94±736.68 | 10837.63±357.66 |  | 1 |
| 96 | C9H8O2 | Cinnamic acid | Phenols | Phenolic acid and their derivatives | 3 | 471.92±44.6 | 610.15±90.82 | 808.03±61.9 | 1096.2±117.35 |  |  |
| 97 | C8H8O4 | Vanillic acid* | Phenols | Phenolic acid and their derivatives | 1 | 3203.29±100.42 | 5382.53±71.25 | 6788.42±176.07 | 11073.75±431.98 |  | 2 |
| 98 | C7H6O5 | Gallic acid* | Phenols | Phenolic acid and their derivatives | 1 | 3554.39±89.7 | 4830.91±339.83 | 7946.89±1236.7 | 11149.92±712.51 | Multitaste | 2 |
| 99 | C8H8O3 | 2-hydroxymethyl benzoic acid | Phenols | Phenolic acid and their derivatives | 3 | 33.88±4.67 | 35.11±1.95 | 41.13±2 | 112.08±4.09 |  | 1 |
| 100 | C13H16O9 | 1-O-Gentisoyl-β-D-glucoside | Phenols | Phenolic acid and their derivatives | 3 | 279.11±43.15 | 488.16±101.64 | 600.53±87.23 | 1158.76±253.09 |  |  |
| 101 | C13H16O10 | 3-O-Galloyl-D-glucose | Phenols | Phenolic acid and their derivatives | 1 | 1558.75±34.48 | 3989.24±222.52 | 6081.76±507.08 | 11948.5±741.65 |  | 2 |
| 102 | C9H10O3 | 2-Hydroxy-3-phenylpropanoic acid | Phenols | Phenolic acid and their derivatives | 2 | 86.94±7.12 | 124.52±7.18 | 51.62±1.47 | 120.5±5.88 |  | 4 |
| 103 | C9H8O3 | 2-Hydroxycinnamic acid | Phenols | Phenolic acid and their derivatives | 3 | 2239.22±47.48 | 2498.24±71.44 | 645.97±15.81 | 1216.54±37.94 |  | 4 |
| 104 | C10H12O3 | Propyl 4-hydroxybenzoate | Phenols | Phenolic acid and their derivatives | 3 | 24.7±2.39 | 17.01±2.83 | 9.54±1.39 | 12.4±1.96 |  | 4 |
| 105 | C12H14O5 | Methyl sinapate | Phenols | Phenolic acid and their derivatives | 3 | 5.28±1 | 11.61±0.57 | 9.16±1.92 | 12.74±1.3 |  |  |
| 106 | C8H8O4 | 3,4-Dihydroxybenzeneacetic acid | Phenols | Phenolic acid and their derivatives | 3 | 1001.22±103.15 | 1229.95±83.35 | 1320.96±56.56 | 1283.45±40.2 |  |  |
| 107 | C9H10O3 | Veratraldehyde | Phenols | Phenolic acid and their derivatives | 2 | 3239.31±132.49 | 5400.76±146.12 | 8863.72±1031.38 | 12962.58±135.16 |  | 2 |
| 108 | C9H8O3 | 3-Hydroxycinnamic acid | Phenols | Phenolic acid and their derivatives | 3 | 2379.81±235.74 | 2506.66±287.34 | 568.53±100.72 | 1302.42±100.7 |  | 4 |
| 109 | C11H12O4 | 3,4-Dimethoxycinnamic acid | Phenols | Phenolic acid and their derivatives | 1 | 4278.92±38.01 | 6579.09±209.72 | 2624.07±92 | 1312.16±84.55 |  | 4 |
| 110 | C7H6O2 | 2-Hydroxybenzaldehyde (Salicylaldehyde) | Phenols | Phenolic acid and their derivatives | 3 | 41.38±0.83 | 80.65±3.65 | 51.57±6.78 | 131.84±10.28 |  | 1 |
| 111 | C17H12O8 | 3,3',4-O-Trimethylellagic acid | Phenols | Phenolic acid and their derivatives | 3 | 0±0 | 663.91±87.41 | 1857.79±767.91 | 1342.13±197.65 |  |  |
| 112 | C9H10O4 | Homovanillic acid | Phenols | Phenolic acid and their derivatives | 3 | 89.64±5.25 | 216.8±6.74 | 153.42±4.48 | 141.2±7.02 |  | 6 |
| 113 | C9H8O2 | p-Coumaraldehyde | Phenols | Phenolic acid and their derivatives | 2 | 84.57±1.66 | 163.27±3.49 | 125.88±12.07 | 143±6.44 |  | 6 |
| 114 | C14H12O11 | Chebulic acid | Phenols | Phenolic acid and their derivatives | 1 | 165.83±21.64 | 270.62±46.09 | 2901.69±364.72 | 1432.94±52.3 |  | 5 |
| 115 | C7H6O2 | 4-Hydroxybenzaldehyde | Phenols | Phenolic acid and their derivatives | 1 | 3302.07±203.88 | 7241.87±493.19 | 6121.89±382.8 | 14484.29±291.38 |  | 1 |
| 116 | C9H8O3 | p-Coumaric acid | Phenols | Phenolic acid and their derivatives | 2 | 212±38.36 | 252.41±16.95 | 85.63±32.59 | 145.2±17.68 |  |  |
| 117 | C16H16O8 | 5-O-Caffeoylshikimic acid | Phenols | Phenolic acid and their derivatives | 3 | 0±0 | 0±0 | 98.99±10.11 | 155.98±8.29 |  | 2 |
| 118 | C9H8O3 | α-Hydroxycinnamic acid | Phenols | Phenolic acid and their derivatives | 3 | 2311.03±165.32 | 2646.47±27.72 | 677.49±89.27 | 1610.28±270.47 |  | 4 |
| 119 | C8H8O3 | 3-Methylsalicylic acid | Phenols | Phenolic acid and their derivatives | 3 | 110.44±3.3 | 98.27±12.46 | 132.02±18.42 | 161.17±11.44 |  |  |
| 120 | C10H8O3 | 3,4-Methylenedioxycinnamaldehyde* | Phenols | Phenolic acid and their derivatives | 1 | 505.46±18.36 | 656.8±27.11 | 678.11±92.93 | 16929.49±527.98 |  | 1 |
| 121 | C15H20O10 | Glucosyringic acid | Phenols | Phenolic acid and their derivatives | 2 | 32.99±10.77 | 97.95±2.54 | 131.56±13.95 | 169.52±2.25 |  | 3 |
| 122 | C6H4O4 | Coumalic acid | Phenols | Phenolic acid and their derivatives | 3 | 2412.76±39.59 | 2311.58±98.4 | 2269.32±100.31 | 1718.57±1130.77 |  |  |
| 123 | C8H9NO2 | (R)-Mandelamide | Phenols | Phenolic acid and their derivatives | 2 | 187.89±12.5 | 348.87±57.78 | 558.68±183.75 | 1776.51±59.45 |  | 1 |
| 124 | C15H8O8 | 3-O-Methylellagic acid | Phenols | Phenolic acid and their derivatives | 1 | 35±5.1 | 65.64±7.96 | 112.61±25.1 | 188.34±7.23 |  | 2 |
| 125 | C7H6O4 | Protocatechuic acid | Phenols | Phenolic acid and their derivatives | 1 | 5064.3±112.73 | 6790.78±17.1 | 10068.14±1359.35 | 19228.19±637.19 | Multitaste | 1 |
| 126 | C6H6O3 | 1,3,5-Benzenetriol | Phenols | Phenolic acid and their derivatives | 1 | 1245.89±70.14 | 1633.04±98.5 | 1670.81±298.57 | 1989.35±34.46 | Sweetness |  |
| 127 | C8H7NO3 | 2-(Formylamino)benzoic acid | Phenols | Phenolic acid and their derivatives | 3 | 321.3±19.2 | 368.66±15.63 | 93.09±10.49 | 200.24±1.58 |  | 4 |
| 128 | C8H8O5 | Methyl gallate | Phenols | Phenolic acid and their derivatives | 1 | 790.7±44.93 | 1818.27±29.47 | 1777.04±75.94 | 2007.01±51.26 |  | 3 |
| 129 | C11H12O3 | p-Coumaric acid ethyl ester | Phenols | Phenolic acid and their derivatives | 3 | 3064.04±110.24 | 3702.17±52.84 | 864.38±96.86 | 2015.81±17.02 | Bitterness | 4 |
| 130 | C8H8O5 | 4-O-Methylgallic acid | Phenols | Phenolic acid and their derivatives | 1 | 783.99±23.67 | 1794.45±51.73 | 1900.16±82.22 | 2064.89±66.76 |  | 3 |
| 131 | C10H10O3 | p-Coumaric acid methyl ester | Phenols | Phenolic acid and their derivatives | 3 | 2362.41±42.87 | 6493.3±95.41 | 5955.33±882.41 | 20805.02±89.85 | Bitterness | 1 |
| 132 | C16H18O8 | 5-O-p-Coumaroylquinic acid | Phenols | Phenolic acid and their derivatives | 2 | 92.96±42.21 | 120.4±58.85 | 118.79±6.81 | 212.83±16.95 |  |  |
| 133 | C13H12O7 | p-Coumaroylmalic acid | Phenols | Phenolic acid and their derivatives | 2 | 0±0 | 1171.19±336.66 | 1084.04±120.39 | 2196.64±27.79 |  | 2 |
| 134 | C16H10O8 | 3,3'-O-Dimethylellagic acid | Phenols | Phenolic acid and their derivatives | 3 | 69.68±14.98 | 119.95±1.94 | 172.55±66.49 | 220.79±13.82 |  |  |
| 135 | C12H16O3 | Elemicin | Phenols | Phenolic acid and their derivatives | 2 | 465.42±22.45 | 423.95±20.52 | 322.01±53.19 | 2217.22±34.2 |  | 1 |
| 136 | C13H16O10 | 6-O-Galloyl-β-D-glucose | Phenols | Phenolic acid and their derivatives | 1 | 7488.61±178.05 | 11378.67±698.35 | 13713.45±1272.99 | 22205.36±1152.5 |  | 2 |
| 137 | C16H18O9 | Chlorogenic acid (3-O-Caffeoylquinic acid) | Phenols | Phenolic acid and their derivatives | 2 | 542.35±31.69 | 169.35±42.15 | 112.58±34.75 | 238.15±32.53 |  | 4 |
| 138 | C7H6O4 | 2,4-Dihydroxybenzoic acid | Phenols | Phenolic acid and their derivatives | 3 | 6228.89±354.55 | 8634.01±161.58 | 12171.47±1736.52 | 24468.1±1065.73 |  | 1 |
| 139 | C11H12O4 | Sinapinaldehyde | Phenols | Phenolic acid and their derivatives | 1 | 893.17±25.72 | 1310.44±23.62 | 429.81±2.08 | 246.98±2.44 |  | 4 |
| 140 | C9H8O3 | Caffeic aldehyde | Phenols | Phenolic acid and their derivatives | 2 | 1002.2±40.13 | 1424.07±42.1 | 1632.33±251.21 | 2507.35±152.84 |  | 2 |
| 141 | C10H10O3 | Methyl Hydroxycinnamate | Phenols | Phenolic acid and their derivatives | 1 | 147.41±2.4 | 251.29±5.19 | 271.4±31.19 | 253.29±12.2 |  | 6 |
| 142 | C9H10O3 | 3-(3-Hydroxyphenyl)-propionic acid | Phenols | Phenolic acid and their derivatives | 2 | 14.03±3.53 | 17.96±2.46 | 22.04±7.92 | 25.69±1.84 |  |  |
| 143 | C10H10O4 | Methyl caffeate | Phenols | Phenolic acid and their derivatives | 2 | 90.9±5.1 | 177.98±12.5 | 227.41±24.47 | 261.79±6.23 |  | 3 |
| 144 | C9H10O4 | Syringaldehyde* | Phenols | Phenolic acid and their derivatives | 1 | 212.34±5.11 | 369.13±0.32 | 217.95±16.2 | 293.79±8.39 |  | 6 |
| 145 | C14H16O10 | 4-O-Galloylquinic Acid | Phenols | Phenolic acid and their derivatives | 2 | 3197.26±531.22 | 2633.37±968.68 | 1397.16±415.27 | 2944.56±687.44 |  |  |
| 146 | C27H24O18 | 1,2,6-Tri-O-galloyl-β-D-glucose | Phenols | Phenolic acid and their derivatives | 1 | 124.55±19.59 | 156.45±11.8 | 155.8±12.55 | 299.42±40.88 |  |  |
| 147 | C14H16O10 | 3-Galloylquinic acid | Phenols | Phenolic acid and their derivatives | 3 | 3406.45±647.08 | 2710.06±942.42 | 1413.85±507.26 | 3017.03±707.32 | Umaminess |  |
| 148 | C9H10O5 | Methyl 3-O-Methyl Gallate | Phenols | Phenolic acid and their derivatives | 3 | 53.1±5.92 | 80±7.91 | 127.26±12.31 | 303.16±14.75 |  | 1 |
| 149 | C13H12O9 | 2-Caffeoyl-L-tartaric acid | Phenols | Phenolic acid and their derivatives | 3 | 179.94±11.6 | 206.64±30.79 | 0±0 | 309.73±49.84 |  | 4 |
| 150 | C8H8O3 | Mandelic acid | Phenols | Phenolic acid and their derivatives | 3 | 76.26±2.07 | 215.21±173.82 | 266.14±21.1 | 309.82±14.98 |  |  |
| 151 | C27H24O18 | 1,3,6-Tri-O-galloyl-β-D-glucose | Phenols | Phenolic acid and their derivatives | 1 | 110.48±15.71 | 195.02±13.23 | 156.78±26.93 | 310.72±40.4 |  |  |
| 152 | C14H14O9 | 5-Galloylshikimic acid | Phenols | Phenolic acid and their derivatives | 3 | 126.16±0.66 | 151.56±1.24 | 169.06±30.95 | 311.21±1.1 |  | 1 |
| 153 | C11H12O4 | Ferulic acid methyl ester | Phenols | Phenolic acid and their derivatives | 2 | 702.94±38.34 | 910.66±17.36 | 227.39±14.81 | 311.39±16.43 |  | 4 |
| 154 | C10H12O5 | Methyl Syringate | Phenols | Phenolic acid and their derivatives | 3 | 56.99±13.6 | 116.94±8.92 | 78.26±9.96 | 315.67±15.58 |  | 1 |
| 155 | C10H10O4 | Ferulic acid* | Phenols | Phenolic acid and their derivatives | 1 | 794.69±12.68 | 1169.98±4.69 | 1602.71±80.34 | 3180.32±33.28 |  | 1 |
| 156 | C10H10O4 | Isoferulic Acid | Phenols | Phenolic acid and their derivatives | 1 | 825.38±36.08 | 1387.84±105.22 | 1753.55±66.04 | 3281.4±187.44 |  | 2 |
| 157 | C14H14O9 | Feruloyltartaric acid (Fertaric acid) | Phenols | Phenolic acid and their derivatives | 3 | 1305.76±54.09 | 3233.75±145.54 | 7448.35±302.91 | 3380.36±217.22 |  | 5 |
| 158 | C7H8O2 | 3,5-Dihydroxytoluene | Phenols | Phenolic acid and their derivatives | 2 | 0±0 | 0±0 | 22.88±1.86 | 34.26±3.61 |  | 2 |
| 159 | C10H10O3 | 4-Methoxycinnamic acid | Phenols | Phenolic acid and their derivatives | 3 | 180.97±65.58 | 47.98±4.66 | 0±0 | 34.61±1.69 |  |  |
| 160 | C8H8O5 | 3-O-Methylgallic acid | Phenols | Phenolic acid and their derivatives | 1 | 933.19±8.76 | 1595.75±90.04 | 2120.62±129.23 | 3518.57±177.06 |  | 2 |
| 161 | C34H28O22 | 1,2,3,4-Tetragalloyl-alpha-D-glucose | Phenols | Phenolic acid and their derivatives | 1 | 35.26±8.94 | 67.24±6.35 | 132.86±58.87 | 361.87±43.74 |  | 1 |
| 162 | C9H10O4 | Vanillic acid methyl ester | Phenols | Phenolic acid and their derivatives | 2 | 94.97±5.43 | 151.61±7.41 | 167.99±33.8 | 373.07±6.01 |  | 1 |
| 163 | C7H6O5 | 2,3,4-Trihydroxybenzoic acid | Phenols | Phenolic acid and their derivatives | 3 | 30.84±3.23 | 127.8±8.96 | 400.45±57.65 | 379.95±19.67 |  | 3 |
| 164 | C34H28O22 | 1,2,3,6-Tetra-O-galloyl-β-D-glucose | Phenols | Phenolic acid and their derivatives | 2 | 36.64±8.96 | 68.47±14.31 | 131.1±44.47 | 383.36±30.18 |  | 1 |
| 165 | C10H12O5 | Propyl gallate | Phenols | Phenolic acid and their derivatives | 3 | 111.56±6.07 | 199.79±10.25 | 155.46±10.8 | 383.59±15.89 | Bitterness | 1 |
| 166 | C28H22O17 | 3,4,5-Tri-O-Galloylshikimic acid | Phenols | Phenolic acid and their derivatives | 3 | 229.27±18.78 | 301.39±15.43 | 320.5±29.77 | 385.17±18.13 |  |  |
| 167 | C9H10O4 | Methyl 2,4-dihydroxyphenylacetate | Phenols | Phenolic acid and their derivatives | 2 | 231.73±14.33 | 365.02±14.33 | 357.19±23.18 | 387.02±8.61 |  | 6 |
| 168 | C10H12O4 | 3,4-Dimethoxyphenyl acetic acid | Phenols | Phenolic acid and their derivatives | 3 | 108.87±7.48 | 123.27±34.76 | 224.61±64.25 | 389.47±26.98 |  |  |
| 169 | C9H10O4 | 2,6-Dimethoxybenzoic acid | Phenols | Phenolic acid and their derivatives | 3 | 102.57±2.61 | 158.35±6.37 | 167.99±36.83 | 390.85±12.38 |  | 1 |
| 170 | C9H10O4 | (S)-2-Hydroxy-3-(4-Hydroxyphenyl)Propanoic Acid | Phenols | Phenolic acid and their derivatives | 2 | 222.21±8.51 | 320.67±19.33 | 352.77±28.16 | 393.91±14.74 |  | 3 |
| 171 | C9H10O4 | 4-Hydroxyphenyllactic Acid | Phenols | Phenolic acid and their derivatives | 3 | 224.11±6.69 | 349.75±17.81 | 354.33±34.53 | 398.08±11.64 |  | 3 |
| 172 | C8H10O2 | 4-Ethoxyphenol | Phenols | Phenolic acid and their derivatives | 3 | 174.7±12.79 | 212.43±4.23 | 241.62±24.45 | 398.3±3.1 |  | 1 |
| 173 | C11H12O5 | Sinapic acid | Phenols | Phenolic acid and their derivatives | 3 | 80.47±3.21 | 149.14±8.35 | 215.32±32.55 | 421.43±40.26 | Bitterness | 2 |
| 174 | C9H10O5 | Syringic acid* | Phenols | Phenolic acid and their derivatives | 1 | 1438.47±22.57 | 2456.93±86.82 | 2656.88±295.1 | 4258.35±124.89 |  | 2 |
| 175 | C27H30O15 | Quercetin-3,7-Di-O-rhamnoside | Phenols | Flavonoids | 3 | 0±0 | 22.03±9 | 38.55±17.56 | 0±0 |  |  |
| 176 | C21H20O12 | Quercetin-7-O-glucoside | Phenols | Flavonoids | 2 | 56.6±13.73 | 284.66±85.39 | 422.35±91.61 | 0±0 |  | 6 |
| 177 | C21H18O12 | Kaempferol-3-O-glucuronide | Phenols | Flavonoids | 3 | 0±0 | 0±0 | 426.42±28.17 | 0±0 |  | 5 |
| 178 | C21H22O11 | Taxifolin-3-O-rhamnoside (Astilbin) | Phenols | Flavonoids | 3 | 45.54±10.13 | 69.91±30.14 | 55.5±26.81 | 0±0 |  |  |
| 179 | C15H14O6 | Epicatechin | Phenols | Flavonoids | 3 | 0±0 | 0±0 | 64.36±12.74 | 0±0 | Bitterness | 5 |
| 180 | C21H20O13 | Myricetin-3-O-β-D-glucoside | Phenols | Flavonoids | 3 | 175.31±15.86 | 180.2±5.4 | 134.56±21.17 | 0±0 |  | 4 |
| 181 | C20H18O12 | Myricetin-3-O-xyloside | Phenols | Flavonoids | 3 | 0±0 | 0±0 | 26.43±20.69 | 0±0 |  |  |
| 182 | C23H24O12 | Tricin-7-O-Glucoside | Phenols | Flavonoids | 3 | 288.37±9.6 | 0±0 | 277.01±117.94 | 0±0 |  |  |
| 183 | C15H12O4 | Dihydrochrysin | Phenols | Flavonoids | 3 | 135.12±12.23 | 109.44±4.28 | 321.91±52.72 | 44.09±4.53 | Bitterness | 5 |
| 184 | C20H22O7 | 6,7,8,3',4'-Pentamethoxyflavanone | Phenols | Flavonoids | 2 | 62.31±24 | 117.09±13.89 | 184.34±27.11 | 448.06±47.04 |  | 1 |
| 185 | C16H14O4 | 3-Deoxysappanchalcone | Phenols | Flavonoids | 2 | 385.07±133.47 | 403.07±11.36 | 640.98±395.35 | 452.19±44.22 |  |  |
| 186 | C20H18O10 | Kaempferol-3-O-arabinoside (Juglanin) | Phenols | Flavonoids | 3 | 0±0 | 0±0 | 257.35±47.33 | 493.61±117.59 |  | 2 |
| 187 | C15H10O7 | Herbacetin | Phenols | Flavonoids | 3 | 148.01±11.31 | 220.17±0.6 | 335.65±49.83 | 495.19±21.91 | Bitterness | 2 |
| 188 | C21H22O8 | Nobiletin | Phenols | Flavonoids | 3 | 104.97±18.46 | 172.03±78.72 | 300.94±126.89 | 496.37±265.04 | Bitterness |  |
| 189 | C17H16O4 | Baphinitone | Phenols | Flavonoids | 3 | 554.59±38.44 | 498.67±2.79 | 234.22±41.25 | 5074.65±107.16 |  | 1 |
| 190 | C15H12O4 | 3,9-Dihydroxypterocarpan | Phenols | Flavonoids | 3 | 85.86±9.01 | 89.7±4 | 32.24±7.06 | 52.79±1.21 |  | 4 |
| 191 | C22H22O12 | Isorhamnetin-7-O-glucoside (Brassicin) | Phenols | Flavonoids | 3 | 514.42±148.05 | 396.07±40.02 | 673.88±231.86 | 571.37±163.23 |  |  |
| 192 | C16H12O4 | 5-Hydroxy-7-methoxyflavone | Phenols | Flavonoids | 3 | 0±0 | 0±0 | 422.24±31.29 | 584.5±109.02 |  | 2 |
| 193 | C21H22O12 | Taxifolin-3'-O-glucoside | Phenols | Flavonoids | 3 | 41.14±0.74 | 0±0 | 652.27±40.33 | 591.32±32.78 |  | 3 |
| 194 | C18H16O7 | Cirsilineol | Phenols | Flavonoids | 3 | 23.91±4.29 | 33.05±3.01 | 30.48±1.76 | 60.57±1.85 |  | 1 |
| 195 | C15H10O4 | 7,4'-Dihydroxyflavone | Phenols | Flavonoids | 3 | 0±0 | 25.09±3.29 | 40.2±15.99 | 68.89±3.75 | Bitterness |  |
| 196 | C15H10O5 | Baicalein | Phenols | Flavonoids | 3 | 159.05±19.8 | 312.54±15.46 | 355.29±54.34 | 698.53±10.87 |  | 2 |
| 197 | C18H16O8 | 5,7,3'-trihydroxy-6,8,4'-trimethoxyflavone | Phenols | Flavonoids | 3 | 32.21±0.99 | 57.8±6.76 | 46.37±3.87 | 70.17±6.3 |  |  |
| 198 | C17H14O8 | Syringetin | Phenols | Flavonoids | 3 | 43.73±3.76 | 64.71±9.38 | 47.41±4.91 | 71.34±2 |  |  |
| 199 | C21H20O12 | Quercetin-4'-O-glucoside (Spiraeoside) | Phenols | Flavonoids | 3 | 0±0 | 0±0 | 0±0 | 714.32±137.19 |  | 1 |
| 200 | C16H14O5 | 7-O-Methylnaringenin | Phenols | Flavonoids | 3 | 141.97±18.87 | 334.66±11.63 | 235.01±47.61 | 725.18±16.72 |  | 1 |
| 201 | C21H20O12 | Quercetin-3-O-glucoside (Isoquercitrin) | Phenols | Flavonoids | 2 | 351.25±26.61 | 439.15±20.47 | 900.67±153.02 | 730.34±96.13 |  |  |
| 202 | C28H32O14 | Acacetin-7-O-rutinoside (Linarin) | Phenols | Flavonoids | 3 | 54.85±2.35 | 53.12±10.71 | 179.08±23.43 | 73.43±12.82 |  | 5 |
| 203 | C15H12O6 | Carthamidin | Phenols | Flavonoids | 2 | 0±0 | 0±0 | 54.9±6.37 | 74.34±9.22 |  | 2 |
| 204 | C15H10O8 | Myricetin | Phenols | Flavonoids | 3 | 174.98±20.17 | 316.23±31.76 | 419.28±64.19 | 755.55±97.93 | Bitterness | 2 |
| 205 | C18H16O8 | Arcapillin | Phenols | Flavonoids | 2 | 108.21±3.7 | 211.94±34.04 | 287.54±28.07 | 755.69±39.36 |  | 1 |
| 206 | C22H22O10 | Swertisin | Phenols | Flavonoids | 3 | 396.94±62.36 | 500.46±148.24 | 502.45±38.28 | 769.25±27.75 |  |  |
| 207 | C16H12O5 | Acacetin | Phenols | Flavonoids | 3 | 0±0 | 79.2±52.12 | 202.52±71.25 | 77.28±38.86 |  |  |
| 208 | C17H18O5 | 2,4'-Dihydroxy-4,6-dimethoxydihydrochalcone | Phenols | Flavonoids | 3 | 2074.71±28.51 | 3287.8±32.37 | 5473.45±548.82 | 8103.39±88.83 |  | 2 |
| 209 | C15H10O4 | Chrysin | Phenols | Flavonoids | 2 | 137.44±11.36 | 203.4±24.01 | 403.2±98.7 | 859.74±30.45 | Bitterness | 1 |
| 210 | C17H14O8 | 3,7-Dimethylquercetagetin | Phenols | Flavonoids | 3 | 895.22±79.69 | 991.88±57.97 | 886.35±157.84 | 916.99±45.24 |  |  |
| 211 | C30H26O12 | Procyanidin B2 | Phenols | Flavonoids | 3 | 23.73±4.57 | 33.5±5.65 | 61.54±6.95 | 93.07±3.78 | Bitterness | 2 |
| 212 | C20H20O7 | Tangeretin | Phenols | Flavonoids | 3 | 32.05±1.49 | 49.09±5.36 | 71.41±22.45 | 93.41±4.54 | Bitterness |  |
| 213 | C26H28O14 | Isoschaftoside | Phenols | Flavonoids | 3 | 99.48±16.41 | 47.92±15.93 | 109.67±19.01 | 93.84±24.68 |  |  |
| 214 | C21H20O12 | Quercetin-3-O-galactoside (Hyperin) | Phenols | Flavonoids | 2 | 247.54±23.84 | 418.24±21.9 | 599.98±186.36 | 946.74±162.23 |  |  |
| 215 | C27H30O15 | Apigenin-6,8-di-C-glucoside (Vicenin-2) | Phenols | Flavonoids | 3 | 74.6±13 | 39.38±1.63 | 56.25±3.97 | 98.86±5.16 |  |  |
| 216 | C16H16O3 | 4'-Hydroxy-7-methoxyflavan | Phenols | Flavonoids | 3 | 0±0 | 152.88±20.8 | 271.95±54.14 | 1019.32±60.76 |  | 1 |
| 217 | C15H10O8 | Quercetagetin | Phenols | Flavonoids | 3 | 6719.39±321.23 | 3530.47±131.47 | 2142.02±217.56 | 1020.82±129.9 | Bitterness | 4 |
| 218 | C17H14O7 | Tricin (5,7,4'-Trihydroxy-3',5'-dimethoxyflavone) | Phenols | Flavonoids | 3 | 170.35±15.28 | 147.49±8.15 | 217.15±67.97 | 104.9±9.71 |  |  |
| 219 | C15H10O5 | 3,7,4'-Trihydroxyflavone | Phenols | Flavonoids | 3 | 355.64±43.86 | 389.83±9.02 | 626.82±104.71 | 1062.04±101.29 | Bitterness | 2 |
| 220 | C16H12O6 | 8-Methoxyapigenin | Phenols | Flavonoids | 3 | 375.87±14.67 | 610.82±174.27 | 931.99±186.13 | 1101.18±219.77 |  |  |
| 221 | C16H12O2 | 6-Methylflavone | Phenols | Flavonoids | 3 | 495.5±29.42 | 744.24±15.31 | 807.54±111.54 | 1169.72±18.24 |  | 2 |
| 222 | C15H12O4 | Liquiritigenin | Phenols | Flavonoids | 3 | 462.12±167.78 | 512.75±16.58 | 682.13±58.06 | 1248.09±64.54 |  |  |
| 223 | C15H10O6 | Norartocarpetin | Phenols | Flavonoids | 3 | 411.82±11.39 | 560.19±26.13 | 733±168.25 | 1315.09±28.3 |  | 1 |
| 224 | C15H10O7 | Morin | Phenols | Flavonoids | 3 | 61.44±4.89 | 127.26±2.1 | 28.32±7.47 | 13.24±8.68 | Bitterness | 4 |
| 225 | C16H14O2 | 4'-Methoxychalcone | Phenols | Flavonoids | 2 | 25.27±10.82 | 86.15±2.22 | 86.77±40.43 | 138.04±41.95 |  |  |
| 226 | C21H20O11 | Luteolin-6-C-glucoside (Isoorientin) | Phenols | Flavonoids | 3 | 131.05±26.81 | 83.82±14.45 | 130.48±33.92 | 150.71±36.34 |  |  |
| 227 | C21H20O11 | Luteolin-8-C-glucoside (Orientin) | Phenols | Flavonoids | 3 | 154.96±24.87 | 90.78±16.03 | 205.1±27.61 | 154.46±3.96 |  |  |
| 228 | C21H21O12+ | Delphinidin-3-O-glucoside (Mirtillin) | Phenols | Flavonoids | 3 | 334.37±6.26 | 410.85±17.59 | 566.14±85.33 | 1564.47±49.3 |  | 1 |
| 229 | C22H22O10 | Glycitin | Phenols | Flavonoids | 3 | 11576.1±368.05 | 17106.33±521.82 | 17718.03±2390.51 | 17008.09±117.4 | Bitterness |  |
| 230 | C15H12O7 | Taxifolin | Phenols | Flavonoids | 2 | 145.36±8.77 | 173.45±12 | 140.42±12.82 | 173.03±10.33 | Bitterness |  |
| 231 | C15H12O5 | Butin | Phenols | Flavonoids | 3 | 524.74±9.86 | 695.88±10.56 | 814.54±160.8 | 1741.48±138.49 |  | 1 |
| 232 | C16H12O6 | Diosmetin | Phenols | Flavonoids | 3 | 499.96±224.93 | 657.35±89.29 | 787.34±92.92 | 1741.83±74.78 |  | 1 |
| 233 | C16H12O6 | Rhamnocitrin | Phenols | Flavonoids | 3 | 372.19±217.95 | 560.95±70.11 | 884.4±208.42 | 1763.87±324.25 |  |  |
| 234 | C16H12O6 | Chrysoerio | Phenols | Flavonoids | 3 | 358.41±9.38 | 681.54±12.99 | 939.24±138.95 | 1787.95±37.39 | Bitterness | 2 |
| 235 | C18H20O4 | 5-O-Methyllatifolin | Phenols | Flavonoids | 1 | 110.1±10.75 | 112.79±9.97 | 678.35±88.54 | 190.41±4.92 |  | 5 |
| 236 | C15H10O7 | Quercetin | Phenols | Flavonoids | 3 | 4066.36±433.61 | 8229.66±1016.08 | 13058.75±1500.85 | 20609±1194.91 | Bitterness | 2 |
| 237 | C16H12O5 | 7,8-Dihydroxy-4'-methoxyisoflavone | Phenols | Flavonoids | 2 | 454.56±6.51 | 936.84±50.31 | 1567.45±257.92 | 2085.93±40.13 |  | 3 |
| 238 | C16H12O5 | Calycosin | Phenols | Flavonoids | 3 | 674.59±14.59 | 1146.57±6.59 | 1810.41±202.38 | 2107.51±33.33 |  | 3 |
| 239 | C15H12O5 | Pinobanksin | Phenols | Flavonoids | 3 | 113.42±2.94 | 129.88±0.6 | 51.95±5.93 | 21.73±4.39 |  | 4 |
| 240 | C16H14O6 | Dihydrokaempferide | Phenols | Flavonoids | 3 | 302.37±34.88 | 506.37±99.42 | 1145.87±294 | 2180.55±227.52 |  | 2 |
| 241 | C15H12O5 | Dihydrobaicalein | Phenols | Flavonoids | 3 | 0±0 | 0±0 | 86.67±28.02 | 226.89±15.63 |  | 1 |
| 242 | C15H12O5 | Naringenin | Phenols | Flavonoids | 3 | 114.97±3.42 | 129.97±4.43 | 55.48±3.74 | 23.12±3.62 |  | 4 |
| 243 | C15H12O5 | Naringenin chalcone | Phenols | Flavonoids | 3 | 572.24±293.32 | 1019.44±459.63 | 1861.13±834.06 | 2516.2±1488.38 |  |  |
| 244 | C21H20O10 | Apigenin-8-C-Glucoside (Vitexin) | Phenols | Flavonoids | 3 | 173.96±26.99 | 104.66±15.31 | 228.91±53.88 | 284.06±112.82 |  |  |
| 245 | C21H20O11 | Quercetin-3-O-rhamnoside(Quercitrin) | Phenols | Flavonoids | 3 | 0±0 | 111.74±3.46 | 0±0 | 300.18±21.89 |  | 1 |
| 246 | C21H20O10 | Apigenin-6-C-glucoside (Isovitexin) | Phenols | Flavonoids | 3 | 189.68±17.71 | 108.83±31.43 | 222.69±25.91 | 302.4±78.37 |  |  |
| 247 | C29H34O16 | Tricin-7-O-neohesperidoside | Phenols | Flavonoids | 3 | 818.54±91.87 | 635.25±49.95 | 168.61±46.66 | 311.1±18.38 |  | 4 |
| 248 | C15H12O5 | Butein | Phenols | Flavonoids | 3 | 0±0 | 0±0 | 0±0 | 32.26±4.93 | Bitterness | 1 |
| 249 | C16H12O7 | Isorhamnetin | Phenols | Flavonoids | 3 | 0±0 | 0±0 | 0±0 | 338.25±24.41 | Bitterness | 1 |
| 250 | C22H22O11 | Diosmetin-7-O-glucoside | Phenols | Flavonoids | 3 | 261.52±28.45 | 300.4±7.74 | 330.27±23.09 | 344.74±103.25 |  |  |
| 251 | C17H14O6 | pilloin | Phenols | Flavonoids | 3 | 23.31±5.44 | 33.8±4.66 | 24.56±4.51 | 34.61±11.48 |  |  |
| 252 | C16H12O7 | Tamarixetin (3,3',5,7-Tetrahydroxy-4'-Methoxyflavone) | Phenols | Flavonoids | 3 | 182.71±25.55 | 270.18±22.49 | 311.65±28.13 | 373.83±50.52 |  |  |
| 253 | C22H22O11 | Hispidulin-7-O-glucoside | Phenols | Flavonoids | 3 | 201.43±38.48 | 212.41±27.3 | 273.02±50.77 | 381.52±23.89 |  |  |
| 254 | C24H20O9 | Cinchonain Ia | Phenols | Flavonoids | 3 | 3952.38±347.84 | 3586.99±161.16 | 3081.13±386.79 | 4032.87±355.37 |  |  |
| 255 | C16H12O8 | Patuletin (Quercetagetin-6-methyl ether) | Phenols | Flavonoids | 3 | 290.55±12.15 | 429.04±86.36 | 405.04±107.63 | 413.2±56.98 |  |  |
| 256 | C22H26O8 | Syringaresinol* | Phenols | Lignans | 1 | 876.61±33.73 | 1340.89±26.67 | 909.82±21.73 | 485.93±14.33 |  | 6 |
| 257 | C22H26O9 | Ciwujiatone | Phenols | Lignans | 1 | 1914.74±139.59 | 4728.8±107.96 | 5748.05±657.26 | 5607.34±401.6 |  | 3 |
| 258 | C20H24O7 | Olivil | Phenols | Lignans | 3 | 40.48±4.72 | 55.88±3.21 | 51.04±1.11 | 67.97±5.86 |  |  |
| 259 | C20H24O6 | Dihydrodehydrodiconiferyl alcohol* | Phenols | Lignans | 1 | 2922.35±106.71 | 4548.46±93.36 | 5532.35±442.19 | 7259.49±51.28 | Sweetness | 3 |
| 260 | C20H24O5 | Anhydrosecoisolariciresinol (AHS) | Phenols | Lignans | 1 | 950.56±84.15 | 1677.84±75.98 | 2745.01±431.27 | 8209.44±82.52 |  | 1 |
| 261 | C22H28O8 | Lyoniresinol | Phenols | Lignans | 2 | 10534.49±193.47 | 11082.24±629.79 | 9714.53±551.89 | 8240.59±111.42 | Bitterness | 4 |
| 262 | C20H22O7 | 8-Hydroxypinoresinol | Phenols | Lignans | 3 | 46.52±8.69 | 93.97±20.13 | 107.17±46.15 | 84.88±34.76 |  |  |
| 263 | C21H24O8 | Fraxiresinol | Phenols | Lignans | 3 | 373.44±41.61 | 674.52±59.29 | 934.9±107.85 | 1145.19±191.93 |  |  |
| 264 | C24H32O9 | Sakuraresinol | Phenols | Lignans | 1 | 81.44±6.21 | 116.09±6.5 | 0±0 | 118.5±9.5 |  | 4 |
| 265 | C23H28O7 | Magnolin | Phenols | Lignans | 2 | 191.48±106.13 | 594.2±20.53 | 888.3±237.69 | 1250.71±158.93 |  |  |
| 266 | C20H22O6 | Pinoresinol | Phenols | Lignans | 3 | 135±3.3 | 172.75±10.32 | 127.18±1.17 | 128.29±7.39 |  | 6 |
| 267 | C20H22O6 | Epipinoresinol | Phenols | Lignans | 2 | 142.99±9.72 | 184.72±1.67 | 138.02±8.68 | 154.62±12.08 |  | 6 |
| 268 | C20H18O7 | Sesaminol | Phenols | Lignans | 3 | 99.85±16.4 | 88.55±7.03 | 123.15±16.23 | 157.11±8.3 |  |  |
| 269 | C20H24O6 | Isolariciresinol | Phenols | Lignans | 3 | 101.35±17.67 | 128.61±14.24 | 110.59±14.86 | 168.67±9.46 |  |  |
| 270 | C20H20O6 | (-)-dihydrosesamin | Phenols | Lignans | 3 | 1091.91±110.36 | 1654.48±68.19 | 1766.5±178.11 | 1942.22±64.93 |  |  |
| 271 | C20H20O6 | Piperitol | Phenols | Lignans | 3 | 116.77±15.24 | 190.15±13.77 | 156.27±11.47 | 209.63±18.04 |  |  |
| 272 | C20H22O6 | Dehydrodiconiferyl alcohol | Phenols | Lignans | 2 | 940.56±42.09 | 1641.4±1107.14 | 1410.1±39.63 | 2267.36±176.65 |  |  |
| 273 | C27H36O12 | Lyoniside | Phenols | Lignans | 2 | 2849.31±36.41 | 2814.25±175.57 | 2815.63±231.8 | 2686.67±97.96 | Bitterness |  |
| 274 | C27H36O12 | Nudiposide | Phenols | Lignans | 1 | 2811.2±57.15 | 2840.02±107.99 | 2913.97±105.13 | 2757.1±191.98 | Sweetness |  |
| 275 | C26H34O11 | Isolariciresinol-9'-O-glucoside | Phenols | Lignans | 3 | 105.74±16.07 | 111.79±9.16 | 131.7±9.43 | 288.05±18.83 |  | 1 |
| 276 | C22H26O8 | Lirioresinol A | Phenols | Lignans | 1 | 5650.38±152.29 | 8428.04±126.47 | 5412.56±87.68 | 2982.45±46.84 |  | 6 |
| 277 | C20H20O4 | Licarin B | Phenols | Lignans | 2 | 6.41±1.41 | 13.74±1.34 | 21.28±6.1 | 32.7±3.51 |  |  |
| 278 | C26H34O11 | Lariciresinol-4'-O-glucoside | Phenols | Lignans | 3 | 0±0 | 0±0 | 161.54±44.14 | 374.67±176.15 |  |  |
| 279 | C26H34O10 | Icariside E4 | Phenols | Lignans | 1 | 50.23±6.76 | 117.26±14.5 | 204.66±26.29 | 422.18±6.32 |  | 2 |
| 280 | C11H14O5 | 3-Hydroxy-1-(4-hydroxy-3,5-dimethoxyphenyl)propan-1-one | Phenols | Other phenols | 1 | 90.01±1.88 | 108.41±8.46 | 304.04±11.95 | 486.58±46.6 |  | 2 |
| 281 | C8H8O2 | 4-Hydroxyacetophenone | Phenols | Other phenols | 3 | 169.91±16.56 | 262.67±37.44 | 254.3±1.8 | 536.6±12.43 |  | 1 |
| 282 | C9H10O2 | 2-Methoxy-4-ethenylphenol | Phenols | Other phenols | 3 | 153.86±6.38 | 269.97±23.88 | 366.56±19.68 | 559.3±16.68 |  | 2 |
| 283 | C8H10O3 | 3,4-Dimethoxyphenol | Phenols | Other phenols | 2 | 3059.82±104.46 | 3282.88±216.25 | 4584.57±684.36 | 5625.51±413.45 |  |  |
| 284 | C10H12O4 | 3-Hydroxy-1-(4-Hydroxy-3-Methoxyphenyl)Propan-1-One | Phenols | Other phenols | 1 | 101.77±7.51 | 215.03±9.02 | 328±26.19 | 611.84±27.86 |  | 2 |
| 285 | C10H12O4 | Acetosyringone | Phenols | Other phenols | 3 | 401.39±7.02 | 701.74±20.62 | 550.51±25.63 | 634.25±9.14 |  | 6 |
| 286 | C9H10O2 | 2'-Hydroxy-4'-Methylacetophenone | Phenols | Other phenols | 3 | 50.24±21.4 | 73.51±9.5 | 42.66±4.07 | 70.57±1.65 |  |  |
| 287 | C8H10O2 | 4-Hydroxyphenylethanol | Phenols | Other phenols | 2 | 231.09±10.54 | 560.14±6.48 | 422.06±19.98 | 773.18±20.41 |  | 2 |
| 288 | C13H18O7 | Gastrodin | Phenols | Other phenols | 2 | 255.03±114.9 | 372.21±106.45 | 1041.98±123.42 | 943.99±75.32 |  | 3 |
| 289 | C8H8O3 | 3,4-Dihydroxyacetophenone | Phenols | Other phenols | 3 | 0±0 | 2.6±0.63 | 7.08±1.84 | 11.98±0.57 |  | 2 |
| 290 | C10H12O5 | C-Veratroylglycol | Phenols | Other phenols | 1 | 413.01±12.92 | 894.17±25.28 | 924.31±60.92 | 1636.79±29.91 |  | 2 |
| 291 | C12H18O4 | 3-(3,4,5-Trimethoxyphenyl)propan-1-ol | Phenols | Other phenols | 2 | 365.56±7.9 | 503±157.54 | 881.28±78.82 | 1738.71±17.64 |  | 1 |
| 292 | C9H12O2 | 3-(4-Hydroxyphenyl)-1-propanol | Phenols | Other phenols | 3 | 16.9±1.86 | 16.13±5.36 | 12.99±4.66 | 17.5±5.65 |  |  |
| 293 | C9H10O2 | 4'-Hydroxypropiophenone | Phenols | Other phenols | 3 | 91.73±1.59 | 153.24±6.68 | 148.1±5.1 | 204.58±10.93 |  | 2 |
| 294 | C16H14O6 | Carpusin | Phenols | Other phenols | 2 | 356.75±58.64 | 657.96±319.27 | 1247.16±252.15 | 2287.97±58.49 |  | 2 |
| 295 | C15H10O7 | Bracteatin | Phenols | Other phenols | 2 | 467.43±222.09 | 511.13±61.12 | 1374.47±378.29 | 2579.78±99.85 |  | 2 |
| 296 | C18H16O5 | 6-hydroxy-2-[2-(3-hydroxy-4-methoxyphenyl)ethyl]chromone | Phenols | Other phenols | 2 | 392.01±16.21 | 726.37±11.61 | 1174.41±105.14 | 2582.22±72.86 |  | 1 |
| 297 | C9H10O4 | 2,4-Dihydroxy-6-methoxyacetophenone | Phenols | Other phenols | 2 | 532.25±26.14 | 835.44±20.03 | 1669.01±91.73 | 2613.87±59.33 |  | 2 |
| 298 | C9H12O4 | 3,4,5-Trimethoxyphenol | Phenols | Other phenols | 1 | 2063.69±29.24 | 2550.9±37.56 | 3485.13±218.99 | 3200.52±105.77 |  | 5 |
| 299 | C8H8O3 | 2',4'-Dihydroxyacetophenone | Phenols | Other phenols | 3 | 0±0 | 18.65±4.35 | 0±0 | 33.13±4.05 |  | 1 |
| 300 | C9H10O3 | 4'-Hydroxy-3'-methoxyacetophenone (Acetovanillone) | Phenols | Coumarins | 3 | 118.85±4.36 | 211.04±19.1 | 250.98±26.79 | 357.07±32.96 |  | 2 |
| 301 | C8H8O4 | 2',4',6'-Trihydroxyacetophenone | Phenols | Coumarins | 3 | 202.73±16.15 | 361.45±12.99 | 278.96±12.5 | 383.82±22.08 |  | 6 |
| 302 | C10H12O | 2',4'-Dimethylacetophenone | Phenols | Coumarins | 2 | 1088.72±62.72 | 1205.14±43.72 | 1162.76±87.64 | 40364.82±1083.24 |  | 1 |
| 303 | C13H16O8 | 1-(4-Hydroxybenzoyl)Glucose | Phenols | Coumarins | 3 | 0±0 | 55.18±53.11 | 274.02±37.72 | 420.94±43.73 |  | 2 |
| 304 | C20H24O5 | 3,4-Divanillyltetrahydrofuran | Phenols | Coumarins | 1 | 946.74±22.54 | 1678.28±47.14 | 2709.66±398.42 | 8381.01±147.33 |  | 1 |
| 305 | C14H22O | 2,4-Di-Tert-Butylphenol | Phenols | Coumarins | 1 | 1062.19±152.89 | 842.25±27.25 | 738.31±216.41 | 860.21±73.47 |  |  |
| 306 | C9H6O4 | Esculetin* | Phenols | Coumarins | 1 | 748.82±36.2 | 848.14±44.8 | 2072.75±220.65 | 4769.32±100.8 | Bitterness | 1 |
| 307 | C9H6O4 | Daphnetin | Phenols | Coumarins | 2 | 1069.36±83.46 | 1755.87±32.76 | 2381.79±322.89 | 4858.42±96.08 |  | 1 |
| 308 | C15H10O4 | 7,8-Dihydroxy-4-phenylcoumarin | Phenols | Coumarins | 2 | 1809.09±167.22 | 3892.72±82.36 | 3154.5±77.71 | 4966.63±192.71 |  | 2 |
| 309 | C11H6O3 | Angelicin | Phenols | Coumarins | 3 | 474±22.01 | 554.77±19.21 | 504.16±28.13 | 565.46±39.03 |  |  |
| 310 | C11H10O5 | Fraxidin | Phenols | Coumarins | 1 | 1527.14±117.17 | 3089.75±58.61 | 3951.59±561.98 | 6522.04±150.26 |  | 2 |
| 311 | C19H12O6 | Dicumarol | Phenols | Coumarins | 2 | 111.48±6.92 | 183.62±6.07 | 207.51±33.68 | 683.11±40.47 | Bitterness | 1 |
| 312 | C9H6O3 | Umbelliferone | Phenols | Coumarins | 2 | 15.95±10.42 | 18.52±6.18 | 28.74±4.29 | 68.81±2.17 | Bitterness |  |
| 313 | C10H8O4 | Isoscopoletin | Phenols | Coumarins | 1 | 2190.64±60.46 | 4559.66±63.14 | 4997.88±616.65 | 7088.16±123.83 |  | 2 |
| 314 | C16H18O9 | Scopolin | Phenols | Coumarins | 2 | 241.33±76.66 | 519.6±22.56 | 651.58±28.5 | 771.04±5.05 | Bitterness | 3 |
| 315 | C17H20O10 | Isofraxidin-7-O-glucoside | Phenols | Coumarins | 2 | 204.1±27.23 | 231.5±46.13 | 524.95±103.33 | 818.5±8.55 |  | 2 |
| 316 | C11H10O5 | isofraxidin | Phenols | Coumarins | 1 | 2681.66±89.07 | 4725.05±68.86 | 5931.3±778.99 | 8223.73±66.87 |  | 2 |
| 317 | C14H16O5 | (+)-Peusedanol | Phenols | Coumarins | 1 | 794.3±109.91 | 1418.67±78.68 | 1319.67±216.64 | 885.87±47.28 |  |  |
| 318 | C10H8O4 | 6,8-Dihydroxy-3-methylisocoumarin | Phenols | Coumarins | 2 | 824.78±22.29 | 1354.33±43.21 | 1209.54±53.38 | 968.3±53.99 |  | 6 |
| 319 | C9H6O2 | Coumarin | Phenols | Coumarins | 1 | 37.25±2.97 | 49.6±3.44 | 68.22±11.72 | 97.42±8.57 | Bitterness |  |
| 320 | C15H16O9 | Cichoriin | Phenols | Coumarins | 3 | 367.9±4.88 | 468.06±64.92 | 733±214.45 | 1487.44±149.75 |  | 1 |
| 321 | C9H8O2 | 3,4-Dihydrocoumarin | Phenols | Coumarins | 2 | 100.73±4.44 | 174.63±11.85 | 140.68±10.55 | 157.34±3.56 |  | 6 |
| 322 | C10H8O4 | Scopoletin | Phenols | Coumarins | 1 | 582.93±3.2 | 1135.31±15.26 | 1192.1±147.35 | 1630.69±34.18 | Bitterness | 3 |
| 323 | C21H20O13 | 11-O-Galloylbergenin | Phenols | Coumarins | 2 | 50.64±23.04 | 0±0 | 95.6±29.65 | 167.01±45.73 |  |  |
| 324 | C10H8O4 | 6-Hydroxy-7-methoxycoumarin | Phenols | Coumarins | 1 | 579.71±10.43 | 1120.32±28.11 | 1229.7±117.11 | 1672.07±15.95 |  | 3 |
| 325 | C12H12O5 | 5,6,7-Trimethoxycoumarin | Phenols | Coumarins | 3 | 926.17±51.25 | 2291.75±55.9 | 2242.2±435.11 | 1735.96±43.3 |  | 6 |
| 326 | C10H8O5 | Fraxetin | Phenols | Coumarins | 1 | 0±0 | 0±0 | 2185.45±134.92 | 1753.56±45.55 | Bitterness | 3 |
| 327 | C12H12O5 | Dimethylfraxetin | Phenols | Coumarins | 3 | 928.21±69.14 | 2306.94±12.6 | 2243.77±427.3 | 1788.46±22.45 |  | 6 |
| 328 | C10H8O4 | 6,7-Dihydroxy-4-methylcoumarin | Phenols | Coumarins | 3 | 501.43±41.97 | 463.64±13.84 | 589.87±48.97 | 1893.98±28.88 |  | 1 |
| 329 | C15H16O9 | Esculin | Phenols | Coumarins | 3 | 559.23±88.51 | 825.56±65.3 | 1279.07±316.98 | 2109.65±561.1 | Bitterness |  |
| 330 | C11H10O5 | leptodactylone | Phenols | Coumarins | 1 | 6732.88±411.96 | 12509.81±77.08 | 15332.9±2013.08 | 21397.04±50.52 |  | 2 |
| 331 | C10H8O2 | 6-Methylcoumarin | Phenols | Coumarins | 3 | 763.31±25.05 | 1168.68±57.32 | 1544.72±123.03 | 3086.62±83.6 | Bitterness | 1 |
| 332 | C11H8O3 | 7,8-Dihydrofurocoumarin | Phenols | Coumarins | 2 | 3183.64±30.42 | 5291±79.27 | 5141.85±670.28 | 3295.1±55.14 |  | 6 |
| 333 | C11H8O3 | 4',5'-Dihydropsoralen | Phenols | Coumarins | 3 | 3266.66±149.63 | 5126.9±242.01 | 5358.15±631.29 | 3341.52±138.46 |  | 6 |
| 334 | C10H12N5O6P | Cyclic 3',5'-Adenylic acid | Nucleotides and their derivates | | 3 | 9.96±0.47 | 9.68±2.78 | 6.73±4.01 | 0±0 |  |  |
| 335 | C10H13N5O5 | Crotonoside | Nucleotides and their derivates | | 3 | 189.43±31.77 | 145.57±7.32 | 360.55±59.72 | 433.59±26.88 |  | 3 |
| 336 | C9H14N2O11P2 | Deoxyuridine-5'-diphosphate | Nucleotides and their derivates | | 3 | 4287.08±296.47 | 2604.75±200.83 | 2671.04±259.77 | 4569.48±117.36 |  | 4 |
| 337 | C10H12N4O5 | Inosine | Nucleotides and their derivates | | 3 | 22.56±3.81 | 35.51±5.82 | 45.68±21.07 | 48.61±8.06 | Bitterness |  |
| 338 | C5H4N4O2 | Xanthine | Nucleotides and their derivates | | 3 | 86.16±13.27 | 280.68±25.24 | 70.02±6.73 | 49.47±9.3 | Bitterness | 6 |
| 339 | C12H17N5O5 | 2-(Dimethylamino)guanosine | Nucleotides and their derivates | | 3 | 30.4±4 | 46.57±1.33 | 48.27±3.37 | 77.09±17.65 |  |  |
| 340 | C9H13N2O9P | Uridine 5'-monophosphate | Nucleotides and their derivates | | 3 | 110.48±3.84 | 172.11±37.88 | 411.47±11.05 | 793.76±48.16 |  | 2 |
| 341 | C10H14N5O7P | Adenosine 5'-monophosphate | Nucleotides and their derivates | | 3 | 32.51±7.66 | 55.61±9.62 | 118.69±5.22 | 81.04±1 | Umaminess | 5 |
| 342 | C9H15N4O8P | AICA ribonucleotide | Nucleotides and their derivates | | 3 | 0±0 | 0±0 | 3200.31±529.44 | 8789.39±179.3 |  | 1 |
| 343 | C4H5N3O | Cytosine | Nucleotides and their derivates | | 3 | 65.95±8.13 | 64.41±6.94 | 119.3±16.21 | 93.56±6.28 |  |  |
| 344 | C6H6N4O2 | 7-Methylxanthine | Nucleotides and their derivates | | 2 | 30.57±3.31 | 51.67±3.71 | 103.73±11.69 | 102.27±5.95 |  | 3 |
| 345 | C4H4N2O2 | Uracil | Nucleotides and their derivates | | 3 | 17.51±5.32 | 39.8±1.78 | 151.58±18.46 | 108.42±9.23 |  | 5 |
| 346 | C11H15N5O4 | 2'-O-Methyladenosine | Nucleotides and their derivates | | 3 | 122.01±10.88 | 142.11±6.77 | 161.15±16.6 | 115±13.85 |  |  |
| 347 | C8H14N3O7P | 5-Aminoimidazole ribonucleotide | Nucleotides and their derivates | | 3 | 6.76±1.98 | 30.95±4.43 | 112.23±8.39 | 115.92±9.63 |  | 3 |
| 348 | C9H13N3O5 | Cytidine | Nucleotides and their derivates | | 3 | 75.07±7.35 | 97.28±4.62 | 178.26±26.03 | 134.55±7.5 |  | 5 |
| 349 | C5H5N5 | Adenine | Nucleotides and their derivates | | 3 | 0±0 | 616.77±88.12 | 942.78±69.01 | 174.82±5.14 |  | 5 |
| 350 | C5H11O7P | 2-Deoxyribofuranose 1-phosphate | Nucleotides and their derivates | | 3 | 32.94±8.86 | 53.92±1.69 | 132.83±19.62 | 177.09±9.21 |  | 3 |
| 351 | C5H5N5O | Guanine | Nucleotides and their derivates | | 2 | 222.76±44.03 | 173.55±29.49 | 280.6±25.04 | 182.32±9.15 |  |  |
| 352 | C14H26N4O11P2 | Citicoline | Nucleotides and their derivates | | 3 | 7.96±2.62 | 6.35±0.78 | 16.26±7.62 | 18.51±2.67 |  |  |
| 353 | C9H14N3O8P | Cytidine 5'-monophosphate | Nucleotides and their derivates | | 3 | 5.27±2.2 | 10.86±4.8 | 27.94±4.87 | 26.66±3.34 |  |  |
| 354 | C10H13N4O8P | Inosine 5'-monophosphate | Nucleotides and their derivates | | 3 | 0±0 | 0±0 | 19.18±9.31 | 30.5±3.43 | Umaminess |  |
| 355 | C11H14NO6+ | Nicotinate D-ribonucleoside | Nucleotides and their derivates | | 3 | 15.37±1.08 | 25.89±7.23 | 40.66±5.82 | 32.17±11.42 |  |  |
| 356 | C10H13N5O5 | Guanosine | Nucleotides and their derivates | | 3 | 134.58±11.67 | 107.79±14.52 | 252.61±34.95 | 323.59±20.7 |  | 2 |
| 357 | C10H13N5O4 | 9-Alpha-Ribofuranosyladenine | Nucleotides and their derivates | | 1 | 2345.37±114.48 | 1866.19±81.02 | 3687.02±306.39 | 3245.59±176.35 |  | 5 |
| 358 | C6H6N4O2 | 3-Methylxanthine | Nucleotides and their derivates | | 3 | 121.07±7.56 | 210.28±15.59 | 344.32±27.6 | 334.04±15.73 |  | 3 |
| 359 | C10H13N5O4 | Adenosine | Nucleotides and their derivates | | 1 | 2468.82±90.87 | 2017.15±190.68 | 4024.98±477.41 | 3414.85±64.91 | Bitterness | 5 |
| 360 | C10H14N5O6P | 2'-Deoxyadenosine-5'-monophosphate | Nucleotides and their derivates | | 3 | 9.21±0.95 | 21.14±6.53 | 49.98±6.07 | 35.84±3.51 |  | 5 |
| 361 | C5H6N2O2 | Thymine | Nucleotides and their derivates | | 3 | 177.44±5.44 | 379.39±39.11 | 301.23±27.71 | 403.78±12.03 |  | 6 |
| 362 | C10H14N5O7P | Adenosine 2'-Phosphate | Nucleotides and their derivates | | 3 | 13.47±1.74 | 21.14±2.76 | 36.63±6.36 | 41.02±5.64 |  |  |
| 363 | C9H12N2O6 | Uridine | Nucleotides and their derivates | | 3 | 23.26±1.76 | 56.61±3.49 | 48.43±2.94 | 42.5±5.57 |  | 6 |
| 364 | C11H8O3 | 2-Methoxy-1,4-naphthoquinone | Quinone | | 3 | 876.9±88.32 | 1306.63±36.82 | 1940.1±192.46 | 4477.91±67.71 |  | 1 |
| 365 | C11H8O3 | Plumbagin | Quinone | | 3 | 782.62±36.19 | 1270.8±10.14 | 1865.64±216.97 | 4523.89±83.64 |  | 1 |
| 366 | C16H12O5 | 3-hydroxy-1,2-dimethoxy-anthraquinone | Quinone | | 3 | 39.33±3.61 | 64.51±0.9 | 66.92±3.69 | 53.4±4.03 |  | 6 |
| 367 | C15H12O3 | Chrysophanol-9-anthrone | Quinone | | 3 | 1090.85±116.6 | 828.54±73.98 | 603.14±128.79 | 882.22±147.37 |  |  |
| 368 | C20H24O9 | Torachrysone-8-O-glucoside | Quinone | | 3 | 2636.85±37.16 | 3246.92±120.68 | 3664.04±226.34 | 8946.4±125.58 |  | 1 |
| 369 | C15H8O6 | Rheic Acid | Quinone | | 3 | 0±0 | 56.84±1.68 | 332.99±148.58 | 1008.76±20.23 |  | 1 |
| 370 | C15H10O5 | Emodin | Quinone | | 3 | 8.28±1.79 | 5.39±1.78 | 9.53±3.01 | 11.72±1.61 |  |  |
| 371 | C15H10O5 | Isoemodin | Quinone | | 3 | 5.09±1.01 | 7.81±2.35 | 9.86±2.81 | 12.74±0.81 |  |  |
| 372 | C15H10O4 | 3-Hydroxy-1-methoxy-9,10-anthraquinone | Quinone | | 2 | 0±0 | 527.02±78.68 | 529.3±63.41 | 1300.2±19.82 |  | 2 |
| 373 | C14H8O3 | 1-Hydroxyanthraquinone | Quinone | | 3 | 286.3±17.02 | 173.23±63.33 | 109.85±16.81 | 180.24±49.76 |  |  |
| 374 | C17H26O4 | Embelin | Quinone | | 1 | 4291.08±636.34 | 2672.73±427.95 | 2316.92±420.76 | 1991.44±497.88 |  |  |
| 375 | C16H12O6 | Xanthorin | Quinone | | 3 | 162.44±5.59 | 305.87±5.77 | 221.77±18.76 | 217.09±15.3 |  | 6 |
| 376 | C15H10O4 | 2-hydroxy-3-hydroxymethyl-anthraquinone | Quinone | | 3 | 622.06±52.48 | 1207.82±147.7 | 1055.29±25.56 | 2784.35±34.19 |  | 1 |
| 377 | C16H16O4 | Eulophiol | Quinone | | 3 | 10.99±1.81 | 12.25±2.63 | 23.22±4.02 | 29.47±5.44 |  |  |
| 378 | C10H10O3 | 3,4-Methylenedioxy cinnamyl alcohol | Others | | 1 | 4739.78±122.18 | 8313.41±101.54 | 8283.05±789.06 | 6664.72±383.55 |  | 6 |
| 379 | C9H16O4 | Eucommiol | Others | | 1 | 311.79±5.24 | 522.13±24.84 | 668.91±63.95 | 1158.31±50.11 |  | 2 |
| 380 | C7H4O6 | Chelidonic acid | Others | | 3 | 0±0 | 0±0 | 100.76±16.04 | 0±0 |  | 5 |
| 381 | C12H16O2 | Senkyunolide A | Others | | 2 | 1296.78±58.65 | 2894.91±294.85 | 3199.17±355.01 | 4558.16±65.98 |  | 2 |
| 382 | C17H18O3 | 2,4,7-Trimethoxy-9,10-dihydrophenanthrene | Others | | 3 | 61.12±28.13 | 79.87±10.67 | 137.61±77.94 | 468.1±16.39 |  | 1 |
| 383 | C6H6O3 | 5-Methoxyfurfural* | Others | | 1 | 3754.13±183.94 | 4489.23±354.57 | 4483.96±611.23 | 5243.33±227.69 |  |  |
| 384 | C8H8O2 | 4-Methoxybenzaldehyde | Others | | 2 | 104.59±2.25 | 191.1±7.29 | 340.95±48.52 | 578.63±39.03 | Bitterness | 2 |
| 385 | C22H43NO | Erucamide | Others | | 3 | 6051.92±553.43 | 4989.4±514.2 | 4516.14±1006.21 | 5791.33±780.08 |  |  |
| 386 | C8H4O3 | Phthalic anhydride | Others | | 1 | 3232.38±327.72 | 3024.94±259.63 | 6298.94±418.95 | 6051.58±703.18 |  | 5 |
| 387 | C12H12O3 | Senkyunolide B | Others | | 1 | 4342.07±1397.3 | 4767.85±1196.76 | 4328.5±416.17 | 6547.44±65.23 |  |  |
| 388 | C5H7NOS | Epigoitrin | Others | | 2 | 2457.66±234.24 | 10654.16±576.47 | 9911.92±3299.09 | 9523.32±682.51 |  |  |
| 389 | C6H8O6 | L-Ascorbic acid | Others | | 2 | 576.82±36.66 | 816.81±18.48 | 1234.43±169.63 | 1004.81±93.3 | Sourness | 5 |
| 390 | C12H16O4 | Senkyunolide I | Others | | 3 | 84.05±7.94 | 181±5.33 | 308.05±36.28 | 1067.04±109.69 |  | 1 |
| 391 | C11H18O | Dihydrojasmone | Others | | 3 | 84.44±17.55 | 211.2±27.84 | 258.5±51.68 | 1088.42±31.24 |  | 1 |
| 392 | C10H10O2 | 4-MethoxycinnaMaldehyde | Others | | 3 | 929.61±105.78 | 785.13±130.03 | 733.1±209.76 | 1113.4±137.44 |  |  |
| 393 | C8H6O3 | Piperonal | Others | | 3 | 170.28±1.12 | 342.08±9.05 | 469.93±57.18 | 1231.35±7.13 | Multitaste | 1 |
| 394 | C17H20N4O6 | Riboflavin | Others | | 3 | 1325.58±50.18 | 455.6±53.18 | 992.17±218.04 | 1278.45±38.44 | Bitterness | 4 |
| 395 | C9H10O3 | 2,6-Dimethoxybenzaldehyde | Others | | 3 | 99.81±4.44 | 145.62±4.71 | 69.11±7.16 | 136.08±5.01 |  | 4 |
| 396 | C6H9NOS | 4-Methyl-5-thiazoleethanol | Others | | 2 | 504±14.51 | 835.65±62.74 | 1055.3±190.44 | 1388.77±12.1 |  |  |
| 397 | C4H6N4O3 | Allantoin | Others | | 3 | 92.41±4.23 | 61.42±3.65 | 65.82±13.45 | 142.55±26.35 |  |  |
| 398 | C6H8O6 | Erythorbic Acid; Isoascorbic Acid | Others | | 3 | 40.32±5.5 | 204.46±17.19 | 204.98±31.44 | 153.91±8.93 |  | 6 |
| 399 | C9H17NO5 | D-Pantothenic Acid | Others | | 3 | 71.95±5.81 | 87.47±3.31 | 96.33±18.62 | 172.04±10.61 | Bitterness |  |
| 400 | C8H8O | 4-Methylbenzaldehyde | Others | | 3 | 313.8±16.73 | 362.77±9.35 | 83.39±1.51 | 182.21±8.12 |  | 4 |
| 401 | C12H16O4 | Methyl 3-(3,4-dimethoxyphenyl)propanoate | Others | | 3 | 347.82±148.95 | 367.6±23.59 | 776.16±374.66 | 1925.14±19.4 |  |  |
| 402 | C12H14O2 | Z-Ligustilide | Others | | 3 | 1209.04±134.99 | 1144.22±152.34 | 819.36±122.57 | 2019.18±596.34 |  |  |
| 403 | C20H28O2 | 4-Oxoretinol | Others | | 3 | 178.79±3.88 | 508.69±53.13 | 800.37±201.55 | 2361.65±218.69 |  | 1 |
| 404 | C11H16O2 | 5,6,7,7a-tetrahydro-4,4,7a-trimethyl-2(4H)-benzofuranone | Others | | 1 | 894.25±17.74 | 1121.98±84.87 | 1415.85±220.12 | 2518.78±34.17 |  | 1 |
| 405 | C10H10O3 | 3-Ethyl-7-hydroxyphthalide | Others | | 2 | 698.58±70.62 | 1217.28±39.26 | 2002.73±216.96 | 2543±146.16 |  | 3 |
| 406 | C15H22O9 | 2,4,6-Trimethoxyphenol glucoside | Others | | 3 | 75.21±38.69 | 78.66±22.35 | 128.14±7.64 | 262.25±53.54 |  |  |
| 407 | C16H22O4 | Butyl isobutyl phthalate | Others | | 1 | 1295.6±205.28 | 1249.41±229.17 | 2747.91±540.41 | 2824.37±209.04 |  |  |
| 408 | C12H14O2 | Butylphthalide | Others | | 2 | 250.82±34.3 | 266.75±10.67 | 253.8±80.93 | 351.15±87.18 |  |  |
| 409 | C8H8O | 3-Methylbenzaldehyde | Others | | 3 | 0±0 | 58.97±6.49 | 0±0 | 36.37±6.66 |  | 6 |
| 410 | C6H9NO3 | Methyl L-pyroglutamate | Alkaloids | | 3 | 153.45±16.09 | 341.81±8.47 | 197.04±51.61 | 438.3±36.63 |  | 1 |
| 411 | C9H7NO2 | Indole-3-carboxylic acid | Alkaloids | | 3 | 9.21±3.57 | 24.17±0.88 | 189.52±32.83 | 445.15±8.19 |  | 1 |
| 412 | C7H13NO2 | Stachydrine | Alkaloids | | 3 | 4135.21±154.42 | 2846.78±164.56 | 2767.08±180.06 | 4494.22±173.78 |  | 4 |
| 413 | C8H11NO | L-Tyramine | Alkaloids | | 3 | 47.25±1.04 | 41.21±7.2 | 55.95±4.12 | 50.66±7.49 |  |  |
| 414 | C4H7NO2 | 5-Hydroxy-2-pyrrolidinone | Alkaloids | | 3 | 25.14±1.99 | 102.82±5.91 | 204.42±22.53 | 539.08±4.72 |  | 1 |
| 415 | C5H5NO2 | Pyrrole-2-carboxylic acid | Alkaloids | | 3 | 13.99±1.97 | 24.75±2.19 | 23.2±3.83 | 55.28±6.93 |  | 1 |
| 416 | C11H18NO+ | Candicine | Alkaloids | | 2 | 14859.78±973.61 | 5100.88±2820.57 | 2745.24±1378.08 | 6081.77±1207.52 |  | 4 |
| 417 | C5H5NO | 3-Hydroxypyridine | Alkaloids | | 3 | 1589.38±100.72 | 3339.85±157.07 | 8382.16±1143.09 | 6832.46±198.36 |  | 3 |
| 418 | C16H12N2O2 | Perlolyrine | Alkaloids | | 2 | 381.76±26.19 | 745.39±14.2 | 1767.72±190.58 | 6908±350.67 |  | 1 |
| 419 | C5H5NO | 4-Hydroxypyridine | Alkaloids | | 3 | 2089.34±164.94 | 4370.36±248.02 | 10350.89±1482.2 | 7998.76±798.07 |  | 5 |
| 420 | C6H6N2O | Nicotinamide | Alkaloids | | 3 | 416.71±29.25 | 867.25±92.48 | 739.04±77.34 | 931.23±62.67 |  | 6 |
| 421 | C8H9NO4 | 4-Pyridoxic acid | Alkaloids | | 3 | 1310.54±59.43 | 2906.67±150.46 | 6923.97±563.84 | 10150.65±357.88 |  | 2 |
| 422 | C5H11N | Piperidine | Alkaloids | | 3 | 34.09±2.05 | 59.19±9.33 | 0±0 | 114.03±4.18 | Bitterness | 1 |
| 423 | C14H12N2O3 | 7-Hydroxy-β-carboline-1-propionic acid | Alkaloids | | 3 | 322.04±5.9 | 369.27±32.52 | 572.75±154.18 | 1292.63±28.94 |  | 1 |
| 424 | C9H7NO | Indole-3-carboxaldehyde | Alkaloids | | 3 | 314.02±8.58 | 676.28±9.2 | 699.81±82.45 | 1334.49±79.05 |  | 2 |
| 425 | C5H14NO+ | Choline | Alkaloids | | 3 | 6489.29±149.85 | 6048.62±98.94 | 9208.14±390.93 | 15093.22±504.23 |  | 1 |
| 426 | C10H7NO2 | Quinoline-4-carboxylic acid | Alkaloids | | 3 | 25.09±16 | 44.51±25.2 | 55.17±7.02 | 166.38±8.84 |  | 1 |
| 427 | C6H5NO3 | 6-Hydroxynicotinic acid | Alkaloids | | 2 | 427.27±35.96 | 853.37±135.04 | 2777±313.11 | 1703.34±35.8 |  | 5 |
| 428 | C9H17NO4 | O-Acetyl-L-carnitine | Alkaloids | | 2 | 47.4±4.92 | 82.03±17.29 | 198.29±35 | 179.81±8.86 |  | 3 |
| 429 | C7H7NO2 | Trigonelline | Alkaloids | | 3 | 1190.18±169.81 | 1223.74±23.09 | 1938.5±70.72 | 1840.05±130.61 |  | 5 |
| 430 | C7H8N2O | 6-Methylnicotinamide | Alkaloids | | 2 | 72.13±3.93 | 104.68±4.91 | 131.12±16.75 | 187.84±7.08 |  | 2 |
| 431 | C6H11NO3 | N-Hydroxypipecolic acid | Alkaloids | | 3 | 96.46±1 | 149.01±12.6 | 198.04±19.47 | 193.73±8.21 |  | 3 |
| 432 | C7H5NO4 | Quinolinic Acid | Alkaloids | | 3 | 61.79±3.19 | 75.49±1.98 | 158.12±17.5 | 195.58±4.33 |  | 3 |
| 433 | C10H9NO2 | Indole-3-acetic acid (IAA) | Alkaloids | | 3 | 267.8±10.48 | 397±21.85 | 292.05±35.19 | 224.66±20.95 |  | 6 |
| 434 | C5H11NO2 | Betaine | Alkaloids | | 2 | 319.78±250.93 | 458.08±156.88 | 2697.93±167.45 | 2295.39±169.38 |  | 3 |
| 435 | C7H8N4O2 | Theophylline | Alkaloids | | 3 | 116.31±39.69 | 87.15±8.1 | 185.87±25.05 | 232.34±20.64 | Bitterness |  |
| 436 | C14H12N2O2 | β-Carboline-1-propanoic acid | Alkaloids | | 3 | 71.07±29.66 | 85.84±31.48 | 150.44±37.34 | 232.7±17.79 |  |  |
| 437 | C6H5NO2 | Nicotinic acid (Vitamin B3) | Alkaloids | | 1 | 606.08±32.26 | 1196.22±91.57 | 2553.17±447.16 | 2338.71±7.8 |  | 3 |
| 438 | C6H5NO2 | Isonicotinic acid | Alkaloids | | 1 | 659.71±24.61 | 1370.81±150.16 | 2670±555.41 | 2658.47±80.1 |  | 3 |
| 439 | C10H9NO3 | 2-oxindole-3-acetic acid | Alkaloids | | 3 | 168.87±13.01 | 413.17±16.68 | 368.23±51.18 | 273.41±52.49 |  | 6 |
| 440 | C27H28N2O4 | Aurantiamide acetate | Alkaloids | | 3 | 9.31±1.99 | 38.37±6.77 | 131.26±23.14 | 286.08±12.98 |  | 2 |
| 441 | C7H16N2O | N-Acetylcadaverine | Alkaloids | | 3 | 55.08±10.36 | 76.44±13.26 | 95.75±15.97 | 286.67±10.62 |  | 1 |
| 442 | C8H11NO3 | Norepinephrine | Alkaloids | | 1 | 6.04±0.57 | 0±0 | 37.47±5.98 | 35.49±2.41 |  | 3 |
| 443 | C8H11NO3 | Pyridoxine | Alkaloids | | 1 | 643.92±51.92 | 912.32±171.6 | 4117.13±676.94 | 3583.26±96.17 |  | 3 |
| 444 | C10H26N4 | Spermine | Alkaloids | | 3 | 287.83±94.16 | 242.34±128.92 | 183.28±27.3 | 368.72±214.97 |  |  |
| 445 | C8H7N | Indole | Alkaloids | | 3 | 515.9±17.24 | 348.75±12.57 | 287.05±43.41 | 384.29±26.45 |  | 4 |
| 446 | C10H13NO4 | 4-[2-formyl-5-(hydroxymethyl)pyrrol-1-yl]butanoic acid | Alkaloids | | 3 | 433.38±48.6 | 1714.69±239.73 | 7816.27±1484.07 | 3908.84±96.7 |  | 5 |
| 447 | C20H23N7O7 | 10-Formyltetrahydrofolic Acid | Alkaloids | | 3 | 33.31±8.76 | 67.1±10.61 | 37.34±11.65 | 40.39±4.85 |  |  |
| 448 | C8H10N4O2 | Purine alkaloid (putatively theobromine or isomer) | Alkaloids | | 2 | 493.08±79.86 | 343.64±34.9 | 447.28±64.88 | 413.3±87.12 | Bitterness |  |
| 449 | C6H14O12P2 | D-Fructose-1,6-biphosphate | Saccharides and their derivates | | 3 | 0±0 | 0±0 | 66.5±40.09 | 0±0 |  |  |
| 450 | C3H7O7P | 3-Phospho-D-glyceric acid | Saccharides and their derivates | | 3 | 0±0 | 0±0 | 105.71±9.79 | 0±0 |  | 5 |
| 451 | C5H10O5 | D-Arabinose* | Saccharides and their derivates | | 1 | 118.93±20.01 | 1795.73±52.53 | 340.75±27.87 | 456.54±58.93 |  | 6 |
| 452 | C7H14O7 | Sedoheptulose* | Saccharides and their derivates | | 1 | 184.88±14.17 | 331.55±31.02 | 367.67±36.78 | 534.15±39.13 |  | 2 |
| 453 | C6H13O9P | D-Glucose-1-phosphate | Saccharides and their derivates | | 3 | 271.8±21.49 | 373.76±38.59 | 603.9±48.42 | 538.85±13.85 |  | 5 |
| 454 | C6H12O5 | L-Fucose | Saccharides and their derivates | | 3 | 182.02±23.17 | 345.29±21.43 | 265.6±31.59 | 552.66±37.59 |  | 1 |
| 455 | C6H10O8 | D-Galactaric acid | Saccharides and their derivates | | 2 | 264.49±14.3 | 344.12±11.48 | 566.12±51.9 | 591.87±29.76 |  | 3 |
| 456 | C6H14O5 | L-Fucitol | Saccharides and their derivates | | 3 | 170.04±2.05 | 317.23±11.47 | 500.92±43.3 | 593.62±30.95 |  | 3 |
| 457 | C3H7O6P | DL-Glyceraldehyde-3-phosphate | Saccharides and their derivates | | 3 | 7.68±2.84 | 19.74±6.11 | 178.1±10.55 | 64.23±8.59 |  | 5 |
| 458 | C6H14O6 | D-Mannitol | Saccharides and their derivates | | 3 | 151.52±24.92 | 64.76±4.74 | 22.02±3.44 | 67.1±5 | Sweetness | 4 |
| 459 | C5H12O5 | Ribitol* | Saccharides and their derivates | | 3 | 46.8±7.41 | 70.14±21.28 | 35.84±3.97 | 73.83±11.61 |  |  |
| 460 | C3H6O4 | DL-Glyceric Acid | Saccharides and their derivates | | 3 | 162.67±50.01 | 238.06±19.73 | 679.1±111.3 | 772.65±27.41 |  | 3 |
| 461 | C6H15O15P3 | d-Myo-inositol-1,4,5-triphosphate | Saccharides and their derivates | | 3 | 38.11±1.81 | 42.95±5.64 | 130.71±14.73 | 88.74±22.94 |  | 5 |
| 462 | C5H12O5 | D-Arabitol | Saccharides and their derivates | | 3 | 56.55±5.46 | 99.69±4.58 | 45.53±7.28 | 95.26±2.56 | Sweetness | 6 |
| 463 | C5H12O5 | L-Arabitol | Saccharides and their derivates | | 3 | 49.62±6.15 | 79.91±4.78 | 32.92±5.62 | 101.71±4.79 | Sweetness | 1 |
| 464 | C5H12O5 | Xylitol | Saccharides and their derivates | | 3 | 56.58±10.54 | 88.08±7.13 | 41.91±5.95 | 112.29±20.32 | Sweetness |  |
| 465 | C6H10O7 | D-Galacturonic acid | Saccharides and their derivates | | 3 | 246.2±19.56 | 505.96±5.45 | 1138.81±58.69 | 1176.45±9.88 |  | 3 |
| 466 | C7H15O10P | D-Sedoheptuiose 7-phosphate | Saccharides and their derivates | | 3 | 37.9±7.32 | 30.76±5.03 | 70.81±10.76 | 120.36±7.25 |  | 1 |
| 467 | C8H15NO6 | N-Acetyl-D-galactosamine | Saccharides and their derivates | | 3 | 27.41±7.02 | 57.2±8.51 | 126.85±8.46 | 124.6±15.64 |  | 3 |
| 468 | C5H10O5 | D-Ribose | Saccharides and their derivates | | 3 | 32.49±1.62 | 225.44±6.56 | 116.85±1.05 | 130.32±53.1 | Sweetness | 6 |
| 469 | C4H8O5 | D-Threonic Acid | Saccharides and their derivates | | 1 | 265.96±6.74 | 451.86±19.21 | 1097.16±172.03 | 1353.39±99.99 |  | 3 |
| 470 | C7H14O6 | D-Pinitol | Saccharides and their derivates | | 2 | 324.1±8.96 | 634.62±17.61 | 1355.44±64.68 | 1471.26±34.66 |  | 3 |
| 471 | C6H13O9P | D-Fructose 6-Phosphate | Saccharides and their derivates | | 3 | 702.75±9.99 | 891.5±59.48 | 1662.74±179.3 | 1512.99±92.43 |  | 5 |
| 472 | C6H12O7 | Gluconic acid | Saccharides and their derivates | | 1 | 958.59±11.93 | 1073.93±84.93 | 1424.93±93.6 | 1771.4±106.72 |  | 3 |
| 473 | C6H15O9P | Sorbitol-6-phosphate | Saccharides and their derivates | | 2 | 42.83±2.06 | 94.39±3.33 | 101.01±6.79 | 184.42±11.36 |  | 2 |
| 474 | C3H6O3 | 2,3-Dihydroxypropanal | Saccharides and their derivates | | 3 | 381.99±96.99 | 483.29±1.07 | 222.35±66.47 | 189.04±7.07 |  |  |
| 475 | C6H8O6 | D-Glucurono-6,3-lactone | Saccharides and their derivates | | 3 | 82.53±4.59 | 152.03±12.95 | 210.44±13.42 | 202.2±12.7 |  | 3 |
| 476 | C6H14O6 | Dulcitol | Saccharides and their derivates | | 3 | 484.83±39.33 | 222.9±22.52 | 72.12±4.91 | 223.8±9.73 | Sweetness | 4 |
| 477 | C6H14O6 | D-Sorbitol | Saccharides and their derivates | | 3 | 615.4±106.73 | 260.33±15.16 | 98.31±6.78 | 249.05±20.53 | Sweetness | 4 |
| 478 | C4H6O5 | 3-Dehydro-L-Threonic Acid | Saccharides and their derivates | | 1 | 463.54±69.75 | 2063.45±267 | 2481.06±219.76 | 2594.35±82.18 |  | 3 |
| 479 | C9H19O11P | 1-(sn-Glycero-3-phospho)-1D-myo-inositol | Saccharides and their derivates | | 3 | 193.81±20.59 | 195.16±9.85 | 171.08±34.53 | 267.78±8.29 |  |  |
| 480 | C5H8O5 | D-Arabinono-1,4-lactone | Saccharides and their derivates | | 2 | 258.87±17.76 | 383.99±61.94 | 416.75±41.19 | 276.32±23.2 |  |  |
| 481 | C6H12O5 | 1,5-Anhydro-D-glucitol | Saccharides and their derivates | | 3 | 24.38±12.14 | 82.37±20.9 | 645.86±57.94 | 286.05±63.03 | Sweetness | 5 |
| 482 | C6H10O5 | 1,6-anhydro-β-D-glucose | Saccharides and their derivates | | 1 | 74.66±11.71 | 133.53±16.54 | 261.34±34.97 | 291.24±27.91 |  | 3 |
| 483 | C5H10O6 | D-Xylonic acid | Saccharides and their derivates | | 3 | 933.62±41.04 | 1745.94±73.29 | 2775.25±305.95 | 3247.82±228.62 |  | 3 |
| 484 | C6H12O6 | Inositol | Saccharides and their derivates | | 1 | 1121.17±75.34 | 2204.44±222.57 | 3120.95±236.96 | 3423.48±330.78 |  | 3 |
| 485 | C6H12O6 | Sorbose* | Saccharides and their derivates | | 1 | 1068±54.13 | 1952.01±61.79 | 3157.42±499.04 | 3484.66±327.7 | Sweetness | 3 |
| 486 | C6H12O6 | D-Fructose* | Saccharides and their derivates | | 1 | 1148.3±73.24 | 2065.09±103.81 | 3201.45±281.05 | 3522.37±303.35 | Sweetness | 3 |
| 487 | C6H12O6 | D-Mannose* | Saccharides and their derivates | | 1 | 1117.21±5.36 | 2186.53±162.55 | 3406.57±293.59 | 3703.79±182.26 | Sweetness | 3 |
| 488 | C6H12O6 | D-Glucose* | Saccharides and their derivates | | 1 | 1126.03±57.09 | 2246.07±80.58 | 3176±159.02 | 3744.24±218.02 | Sweetness | 3 |
| 489 | C6H10O6 | D-Glucono-1,5-lactone | Saccharides and their derivates | | 2 | 116.76±35.49 | 0±0 | 161.07±163.3 | 374.77±25.78 |  |  |
| 490 | C6H12O6 | D-Galactose | Saccharides and their derivates | | 1 | 1350.04±41.15 | 2521.91±273.74 | 3699.37±317.19 | 4210.65±157.41 | Sweetness | 3 |
| 491 | C12H22O11 | D-Sucrose | Saccharides and their derivates | | 3 | 559.71±129.64 | 838.49±283.31 | 5799.55±1054.4 | 605.79±102.31 | Sweetness | 5 |
| 492 | C30H52O26 | Maltopentaose | Saccharides and their derivates | | 3 | 44±4.9 | 60.74±9.15 | 63.38±5.07 | 62.01±13.95 |  |  |
| 493 | C18H32O16 | Maltotriose | Saccharides and their derivates | | 2 | 353.41±119.57 | 394.09±30.29 | 703.87±87.07 | 624.68±90.02 | Sweetness |  |
| 494 | C12H22O11 | Melibiose | Saccharides and their derivates | | 3 | 382±35.69 | 648.41±25.82 | 3371.71±561.58 | 687.76±78.25 |  | 5 |
| 495 | C12H24O11 | Maltitol | Saccharides and their derivates | | 1 | 51.51±7.31 | 72.88±6.49 | 259.75±53.94 | 69.1±6.91 | Sweetness | 5 |
| 496 | C12H22O11 | D-Trehalose | Saccharides and their derivates | | 3 | 676.39±51 | 1057.99±77.81 | 5133.28±1307.94 | 802.28±57.24 | Sweetness | 5 |
| 497 | C12H22O11 | D-Lactose | Saccharides and their derivates | | 2 | 6.78±1.45 | 12.23±3.18 | 98.79±24.01 | 9.3±3.03 | Sweetness | 5 |
| 498 | C12H22O11 | D-Cellobiose | Saccharides and their derivates | | 2 | 46.1±5.15 | 67.03±8.5 | 210.27±33.14 | 97.39±5.02 | Sweetness | 5 |
| 499 | C18H32O16 | Manninotriose | Saccharides and their derivates | | 2 | 54.37±4.29 | 75.54±4.28 | 102.53±16.71 | 109.13±27.9 |  |  |
| 500 | C12H22O11 | Galactinol | Saccharides and their derivates | | 3 | 962.58±249.3 | 1541.81±466.85 | 10663.28±1522.54 | 1096.93±162.02 |  | 5 |
| 501 | C11H20O10 | 6-O-alpha-L-arabinopyranosyl-D-glucopyranose | Saccharides and their derivates | | 3 | 58.06±5.53 | 108.92±32.02 | 145.06±3.65 | 120.08±9.96 |  |  |
| 502 | C24H42O21 | Cellotetraose | Saccharides and their derivates | | 3 | 94.34±5.5 | 120.85±4.89 | 222.05±21.64 | 133.46±3.53 |  | 5 |
| 503 | C12H22O11 | D-Maltose | Saccharides and their derivates | | 2 | 987.55±339.61 | 1551.9±472.28 | 10302.24±1437.43 | 1366.22±217.3 | Sweetness | 5 |
| 504 | C24H42O21 | Stachyose | Saccharides and their derivates | | 3 | 104.8±2.88 | 148.54±4.78 | 233.84±7.86 | 139.93±2.84 | Sweetness | 5 |
| 505 | C24H42O21 | D-Maltotetraose | Saccharides and their derivates | | 3 | 130.26±7.36 | 155.52±8.63 | 179.2±9.45 | 146.08±9.35 |  | 5 |
| 506 | C24H42O21 | Nystose | Saccharides and their derivates | | 3 | 176.52±5.75 | 239.9±31.46 | 315.5±9.12 | 198.69±15.9 |  | 5 |
| 507 | C18H32O16 | D-Melezitose | Saccharides and their derivates | | 3 | 155.76±5.42 | 236.52±20.38 | 402.14±23.12 | 264.73±12.05 |  | 5 |
| 508 | C18H32O16 | Raffinose | Saccharides and their derivates | | 1 | 193.31±21.93 | 257.32±39.62 | 338.2±35.39 | 270.23±39.21 | Sweetness |  |
| 509 | C30H52O26 | Verbascose | Saccharides and their derivates | | 3 | 30.6±3.1 | 30.01±2.07 | 37.12±3.83 | 33.76±3.91 |  |  |
| 510 | C15H20O4 | Abscisic acid | Terpenes | Sesquiterpenes | 3 | 25.62±4.13 | 42.31±4.41 | 37.33±6.14 | 34.61±3.82 |  |  |
| 511 | C15H24O9 | Ajugol | Terpenes | Monoterpenes | 3 | 30.9±22.03 | 0±0 | 28.73±3.26 | 69.87±36.68 |  |  |
| 512 | C11H16O5 | Loganetin | Terpenes | Monoterpenes | 3 | 39.9±3.02 | 75.62±2.41 | 111.64±14 | 200.11±33.46 |  | 2 |
| 513 | C15H22O8 | Bartsioside | Terpenes | Monoterpenes | 2 | 151.65±20.75 | 206.58±19.41 | 242.22±11.02 | 375.98±6.74 |  | 2 |
| 514 | C16H22O9 | Sweroside | Terpenes | Monoterpenes | 2 | 0±0 | 67.62±16.78 | 192.49±90.41 | 412.37±9.72 |  | 2 |
| 515 | C16H22O10 | Geniposidic acid | Terpenes | Diterpenes | 3 | 259.95±22.07 | 354.65±27.4 | 542.03±86.79 | 413.07±112 |  |  |
| 516 | C25H32O14 | 10-Hydroxyoleuropein | Terpenes | Diterpenes | 3 | 0±0 | 0±0 | 1191.14±73.64 | 430.51±307.25 |  | 5 |
| 517 | C19H22O6 | Gibberellic acid | Terpenes | Diterpenes | 3 | 0±0 | 683.3±17.25 | 971.65±69.2 | 859.7±46.58 |  | 3 |
| 518 | C20H32O3 | Cupressic acid | Terpenes | Diterpenes | 2 | 165.25±2.88 | 390.91±33.32 | 678.05±89.86 | 1462.89±36.68 |  | 1 |
| 519 | C20H30O3 | 12-Hydroxyabietic Acid | Terpenes | Diterpenes | 3 | 0±0 | 692.73±82.21 | 836.44±253.95 | 2003.13±98.75 |  | 2 |
| 520 | C22H34O5 | Vitexilactone | Terpenes | Diterpenes | 2 | 344.94±44.49 | 309.76±8.03 | 233.27±19.48 | 353.77±170.16 |  |  |
| 521 | C20H34O2 | Kaurane-16,17-diol | Terpenes | Diterpenes | 3 | 635.65±32.43 | 1788.6±61.63 | 2896.48±666.26 | 4137.84±96.69 |  | 2 |
| 522 | C30H46O4 | Camaldulenic acid | Terpenes | Triterpenes | 2 | 2381.37±108.42 | 7049.05±281.55 | 5645.35±438.62 | 4423.84±75.93 |  | 6 |
| 523 | C30H46O5 | 2,19-Dihydroxy-3-oxours-12-en-28-oic acid | Terpenes | Triterpenes | 2 | 1402.03±35.19 | 3012.94±103.85 | 3154.28±275.65 | 5950.66±69.12 |  | 2 |
| 524 | C30H48O6 | 2,3,19,23-Tetrahydroxyolean-12-en-28-oic acid | Terpenes | Triterpenes | 3 | 27.55±3.19 | 65.09±39.54 | 44.23±1.68 | 61.37±2.07 |  |  |
| 525 | C37H52O10 | 23-O-Galloylarjungenin | Terpenes | Triterpenes | 3 | 41.72±13.19 | 39±16.26 | 74.41±17.29 | 65.6±19.28 | Sourness |  |
| 526 | C30H48O4 | Pomolic acid | Terpenes | Triterpenes | 1 | 2441.22±103.44 | 4566.51±168.88 | 5048.79±464.08 | 7521.45±186.49 |  | 2 |
| 527 | C30H46O4 | Virgatic acid | Terpenes | Triterpenes | 2 | 2701.57±94.09 | 7781.15±38.5 | 8289.07±1440.35 | 7524.48±210.85 |  | 6 |
| 528 | C30H48O | Aborenone | Terpenes | Triterpenes | 3 | 12.6±3.92 | 22.62±1.02 | 40.43±4.95 | 93.71±10.99 |  | 1 |
| 529 | C30H46O | Glochidone | Terpenes | Triterpenes | 3 | 97.35±13.18 | 115.45±11.89 | 114.48±11.28 | 109.87±15.9 |  |  |
| 530 | C30H48O2 | 3-Hydroxylup-20(29)-en-28-al | Terpenes | Triterpenes | 3 | 3311.47±89.68 | 6574.67±254.55 | 7847.66±605.1 | 12807.38±660.86 |  | 2 |
| 531 | C30H48O2 | ursolic aldehyde | Terpenes | Triterpenes | 3 | 29.44±2.77 | 60.96±4.29 | 74.08±10.44 | 130.02±1.13 |  | 2 |
| 532 | C30H44O4 | Semialactone | Terpenes | Triterpenes | 2 | 778.83±42.68 | 1259.75±144.35 | 1364.12±273.7 | 1346.01±209.1 |  |  |
| 533 | C30H48O4 | 2-Hydroxyoleanolic acid | Terpenes | Triterpenes | 1 | 5631.89±370.79 | 10577.12±478.85 | 10872.98±798.79 | 15477.67±268.31 |  | 2 |
| 534 | C30H46O4 | Pomonic acid | Terpenes | Triterpenes | 1 | 68.9±2.52 | 153.92±15.77 | 188.05±20.52 | 159.47±31.47 |  |  |
| 535 | C30H48O6 | 3β,6β,19α,24-Tetrahydroxyurs-12-en-28-oic acid | Terpenes | Triterpenes | 2 | 343.98±19.94 | 275.69±8.86 | 278.24±86.21 | 172.66±11.21 |  |  |
| 536 | C30H48O4 | Maslinic Acid | Terpenes | Triterpenes | 3 | 7029.2±478.27 | 12785.86±366.81 | 13529.68±1276.54 | 18472.31±219.71 |  | 3 |
| 537 | C30H48O4 | 3-epimaslinic acid | Terpenes | Triterpenes | 2 | 752.09±20.41 | 803.94±67.45 | 1230.11±156.72 | 2046.61±746.38 |  |  |
| 538 | C30H46O7 | Barrinic acid | Terpenes | Triterpenes | 3 | 748.06±13.47 | 1262.77±36.76 | 1239.85±17.76 | 2205.15±37.93 | Bitterness | 2 |
| 539 | C30H46O7 | Bartogenic acid | Terpenes | Triterpenes | 3 | 768.83±29.76 | 1281.36±23.27 | 1268.46±61.29 | 2210.53±5.57 | Bitterness | 2 |
| 540 | C30H48O6 | Arjugenin | Terpenes | Triterpenes | 2 | 668.77±25.66 | 576.05±3.57 | 456.83±14.44 | 284.45±13.17 | Sweetness | 4 |
| 541 | C30H48O5 | Tormentic acid | Terpenes | Triterpenes | 1 | 396.11±31.46 | 539.52±30.53 | 453.39±8.74 | 307.39±10.39 |  | 6 |
| 542 | C30H48O3 | Betulinic acid | Terpenes | Triterpenes | 3 | 2119.57±75.76 | 4987.16±189.54 | 3944.16±929.19 | 3276.27±239.69 |  |  |
| 543 | C30H48O3 | Ursolic acid | Terpenes | Triterpenes | 3 | 2130.66±86.25 | 4770.63±366.69 | 3743.67±1034.91 | 3353.36±178.35 |  |  |
| 544 | C30H48O6 | 1,2,3,19-Tetrahydroxyurs-12-en-28-oic acid | Terpenes | Triterpenes | 2 | 692.76±4.25 | 607.3±13.27 | 495.88±11.04 | 347.81±8.93 |  | 4 |
| 545 | C30H48O3 | Oleanolic acid | Terpenes | Triterpenes | 3 | 2096.16±76 | 4861.28±281.73 | 3954.62±877.48 | 3525.75±295.19 |  |  |
| 546 | C30H48O5 | Rutundic acid | Terpenes | Triterpenes | 1 | 557.3±29.26 | 714.61±30.87 | 577.49±25.2 | 360.76±11.59 |  | 6 |
| 547 | C6H6O6 | Aconitic acid | Organic acids and their derivatives | | 3 | 26.54±10.13 | 0±0 | 0±0 | 0±0 |  |  |
| 548 | C3H5O6P | Phosphoenolpyruvate | Organic acids and their derivatives | | 3 | 0±0 | 25.8±3.73 | 57.95±11.98 | 0±0 |  | 5 |
| 549 | C5H8O5 | L-Citramalic acid | Organic acids and their derivatives | | 3 | 0±0 | 19.05±9.92 | 61.3±7.85 | 43.98±4.92 |  | 3 |
| 550 | C6H10O4 | 2-Methylglutaric acid | Organic acids and their derivatives | | 3 | 161.53±8.81 | 265.25±18.44 | 543.18±53.32 | 458.69±14.89 |  | 5 |
| 551 | C6H10O3 | 4-Methyl-2-oxovalerate | Organic acids and their derivatives | | 3 | 31.47±4.19 | 26.38±5.92 | 21.81±1.76 | 46.61±1.4 |  |  |
| 552 | C5H6O4 | Itaconic acid | Organic acids and their derivatives | | 2 | 118.96±1.73 | 225.85±29.93 | 261.5±35.42 | 472.11±6.56 |  | 2 |
| 553 | C7H12O4 | 2-Propylsuccinic acid | Organic acids and their derivatives | | 3 | 8.85±2.25 | 13.48±3.7 | 18.04±5.34 | 47.59±0.77 |  | 1 |
| 554 | C9H8O3 | Phenylpyruvic acid | Organic acids and their derivatives | | 3 | 355.38±118.16 | 1490.58±7.09 | 2636.78±63.22 | 4762.69±224.39 |  | 2 |
| 555 | C6H10O4 | Adipic acid | Organic acids and their derivatives | | 3 | 175.75±9.34 | 283.93±7.2 | 584.17±57.88 | 480.27±16.9 | Sourness | 5 |
| 556 | C6H11NO3 | 4-Acetamidobutyric acid | Organic acids and their derivatives | | 3 | 176.69±5.08 | 504.7±74.71 | 425.04±37.73 | 490.5±43.87 |  | 6 |
| 557 | C7H12O6 | Quinic acid* | Organic acids and their derivatives | | 1 | 2357.29±40.48 | 1431.24±191.63 | 6411.25±656.78 | 5043.77±413.99 | Bitterness | 5 |
| 558 | C11H12O4 | 2-Benzylsuccinic Acid | Organic acids and their derivatives | | 2 | 374.85±10.42 | 557.55±26.7 | 372.87±102.36 | 506.99±21.46 |  |  |
| 559 | C7H10O7 | Homocitrate | Organic acids and their derivatives | | 2 | 317.09±10.04 | 398.59±17.72 | 595.97±54.94 | 517.68±45.51 |  | 5 |
| 560 | C5H6O5 | α-Ketoglutaric acid | Organic acids and their derivatives | | 3 | 106.06±5.52 | 248.85±26.53 | 738.8±69.02 | 527.15±38.85 |  | 5 |
| 561 | C7H7NO2 | 4-Aminobenzoic acid | Organic acids and their derivatives | | 3 | 0±0 | 69.91±3.24 | 111.95±10.96 | 532.65±32.84 |  | 1 |
| 562 | C5H8O3 | 3-Methyl-2-Oxobutanoic acid | Organic acids and their derivatives | | 3 | 2732.28±102.88 | 1983.16±462.64 | 3709.16±1069.71 | 5865.29±388.85 |  |  |
| 563 | C4H6O4 | Acetoxyacetic acid | Organic acids and their derivatives | | 3 | 66.69±12.89 | 182.85±31.65 | 75.14±25.45 | 63.67±7.02 |  |  |
| 564 | C5H10O4 | 2,3-Dihydroxy-3-Methylbutanoic Acid* | Organic acids and their derivatives | | 1 | 456.03±11.89 | 1664.04±108.15 | 1395.66±83.4 | 696.77±36.79 |  | 6 |
| 565 | C6H8O7 | Isocitric Acid* | Organic acids and their derivatives | | 1 | 2182.06±211.73 | 3225.05±1116.32 | 2910.98±146.98 | 768.2±114.88 |  |  |
| 566 | C7H6O2 | Benzoic acid | Organic acids and their derivatives | | 3 | 276.45±52.61 | 307.63±21.83 | 414.82±56.66 | 773.1±91.36 | Sourness | 1 |
| 567 | C5H8O3 | 4-Oxopentanoic Acid | Organic acids and their derivatives | | 3 | 408.03±13.59 | 272.49±64.92 | 499.42±153.79 | 779.43±39.45 |  |  |
| 568 | C9H16O3 | 9-Oxononanoic acid | Organic acids and their derivatives | | 3 | 22.12±4.74 | 22.86±2.53 | 39.63±13.38 | 78.87±3.35 |  |  |
| 569 | C6H18O24P6 | Phytic acid | Organic acids and their derivatives | | 3 | 4.72±0.31 | 4.8±0.08 | 4.8±0.05 | 8.06±0.16 |  | 1 |
| 570 | C6H10O3 | 3-Methyl-2-oxopentanoic acid | Organic acids and their derivatives | | 3 | 19.73±2.84 | 49.88±14.7 | 58.72±3.8 | 83.95±2.52 |  |  |
| 571 | C6H8O7 | Citric acid | Organic acids and their derivatives | | 2 | 184.1±16.78 | 270.79±7.35 | 1376.96±141.04 | 912.37±81.08 | Sourness | 5 |
| 572 | C6H10O4 | 2,2-Dimethylsuccinic acid | Organic acids and their derivatives | | 3 | 4.32±1.61 | 6.58±0.91 | 6.14±0.64 | 9.14±0.92 |  |  |
| 573 | C6H12O4 | Mevalonic acid | Organic acids and their derivatives | | 2 | 1037.61±129.69 | 3268.09±42.07 | 2151.94±82.38 | 1091.22±58.22 |  | 6 |
| 574 | C10H18O4 | Sebacic acid | Organic acids and their derivatives | | 2 | 4.08±0.47 | 6.43±0.9 | 8.63±1.35 | 11.04±0.28 |  |  |
| 575 | C3H4O4 | Tartronate semialdehyde | Organic acids and their derivatives | | 3 | 800.96±126.88 | 704.23±36.75 | 1348.54±409.28 | 1118.41±7.78 |  |  |
| 576 | C9H16O4 | Azelaic acid* | Organic acids and their derivatives | | 1 | 2938.09±34.22 | 4844.89±355.02 | 6723.71±550.52 | 11371.48±235.2 |  | 2 |
| 577 | C4H8O3 | 3-Hydroxybutyric acid | Organic acids and their derivatives | | 3 | 3320.47±506.02 | 3079.07±299.81 | 3837.28±2144.46 | 1197.29±112.12 |  |  |
| 578 | C6H11NO2 | L-Pipecolic Acid | Organic acids and their derivatives | | 2 | 2707.31±66.97 | 6940.14±210.2 | 9204.62±734.28 | 13176.92±314.2 |  | 2 |
| 579 | C4H4O5 | Oxalacetic acid | Organic acids and their derivatives | | 3 | 20.98±1.21 | 49.93±6.26 | 67.36±5.72 | 139.61±6.04 |  | 2 |
| 580 | C8H8O2 | 4-Methylbenzoic acid | Organic acids and their derivatives | | 2 | 3879.11±140.53 | 5737.83±160.97 | 9283.98±1141.39 | 14663.67±356.54 |  | 2 |
| 581 | C7H12O5 | 3-Isopropylmalic Acid* | Organic acids and their derivatives | | 1 | 1513.36±26.39 | 2081.33±19.84 | 1182.97±52.38 | 1598.32±37.03 |  | 4 |
| 582 | C6H12O3 | 2-Hydroxyisocaproic acid* | Organic acids and their derivatives | | 1 | 22168.12±2449.37 | 15287.39±1468.94 | 9153.4±1451.76 | 18083.85±629.92 |  | 4 |
| 583 | C8H6O4 | Piperonylic acid | Organic acids and their derivatives | | 2 | 109.05±7.92 | 190.51±11.21 | 263.43±12.22 | 185.54±13.45 | Bitterness | 5 |
| 584 | C5H8O4 | Glutaric acid | Organic acids and their derivatives | | 3 | 2134.7±101.46 | 2753.58±127.95 | 2852.75±190.04 | 1922.89±18.02 |  | 6 |
| 585 | C3H6O3 | L-Lactic acid | Organic acids and their derivatives | | 3 | 362.46±127.76 | 559.06±34.63 | 244.04±52.23 | 194.52±5.72 | Sourness |  |
| 586 | C2H2O4 | Oxalic acid | Organic acids and their derivatives | | 3 | 2812.85±42.93 | 4604.82±1356.32 | 2214.07±725.97 | 1952.21±54.28 | Sourness |  |
| 587 | C7H7NO2 | Anthranilic acid | Organic acids and their derivatives | | 3 | 78.41±18.24 | 96±10.4 | 189.41±21.66 | 205.86±14.56 | Sweetness | 3 |
| 588 | C4H9NO2 | 2-Aminoisobutyric acid | Organic acids and their derivatives | | 2 | 960.96±136.88 | 978.91±25.42 | 1604.16±80 | 2244.05±208.14 |  | 2 |
| 589 | C5H4O3 | 2-Furoic acid* | Organic acids and their derivatives | | 1 | 10637.76±399.27 | 13334.9±839.03 | 16545.36±2701.82 | 23395.42±412.71 |  |  |
| 590 | C10H7NO4 | 4,8-Dihydroxyquinoline-2-carboxylic acid | Organic acids and their derivatives | | 3 | 79.72±9.07 | 97±6.83 | 133.01±9.85 | 233.99±6.87 |  | 1 |
| 591 | C8H8O2 | Phenylacetic acid | Organic acids and their derivatives | | 3 | 101.86±2.22 | 105.15±6.88 | 137.09±22.2 | 241.7±16.34 |  | 1 |
| 592 | C4H6O5 | Malic acid | Organic acids and their derivatives | | 1 | 280.59±23.82 | 847±75.84 | 2189.75±304.42 | 2527.39±58.52 | Sourness | 3 |
| 593 | C5H8O5 | 3-Hydroxyglutaric acid | Organic acids and their derivatives | | 2 | 234.15±27.02 | 370.95±56.44 | 403.6±46.56 | 261.13±30.74 |  |  |
| 594 | C5H8O3 | 2-Oxovaleric acid | Organic acids and their derivatives | | 3 | 712.53±17.74 | 2006.26±26.06 | 1743.92±219.82 | 2740.52±50.13 |  | 2 |
| 595 | C8H14O4 | Suberic Acid | Organic acids and their derivatives | | 3 | 76.17±7.82 | 116.42±7.19 | 164.68±12.98 | 314.05±6.87 |  | 1 |
| 596 | C4H6O4 | Succinic acid* | Organic acids and their derivatives | | 1 | 1572.8±58.13 | 2611.59±152.97 | 1889.99±359.2 | 3302.31±84.11 | Sourness |  |
| 597 | C6H5NO2 | 2-Picolinic acid | Organic acids and their derivatives | | 3 | 60.8±2.65 | 126.7±16.39 | 604.3±34.61 | 330.44±23.56 |  | 5 |
| 598 | C5H10O3 | β-Hydroxyisovaleric acid | Organic acids and their derivatives | | 3 | 149.75±6.61 | 80±5.86 | 208.42±42.75 | 339.27±11.87 |  | 1 |
| 599 | C5H9NO3 | 5-Aminolevulinic Acid | Organic acids and their derivatives | | 3 | 469.05±11.19 | 570.99±142.49 | 636.55±140.62 | 352.6±115.85 |  |  |
| 600 | C4H4O3 | Succinic anhydride | Organic acids and their derivatives | | 3 | 198.72±3.98 | 209.64±34.15 | 227.95±13.3 | 362.83±0.83 |  |  |
| 601 | C4H8O3 | 2-Hydroxyisobutyric acid* | Organic acids and their derivatives | | 1 | 2368.75±128.94 | 1594.65±199.45 | 203.28±68.35 | 370.77±2.87 |  | 4 |
| 602 | C18H30O2 | Elaidolinolenic acid | Organic acids and their derivatives | | 2 | 1468.77±466.67 | 1668.16±106.12 | 4173.92±2128.23 | 4986.23±50.62 |  |  |
| 603 | C21H36O4 | 2-α-Linolenoyl-glycerol | Lipids | Steroids | 1 | 74.6±3.33 | 138.57±1.16 | 157.49±26.9 | 139.85±20.68 |  |  |
| 604 | C35H69O8P | 1,2-Dipalmitoyl-sn-glycerol 3-phosphate | Lipids | Steroids | 3 | 1.81±0.34 | 4.04±2.23 | 5.32±2.15 | 16.91±0.33 |  | 1 |
| 605 | C26H52NO7P | LysoPC 18:1 | Lipids | Steroids | 1 | 132.08±6.39 | 586.55±24.71 | 508.71±117.37 | 449.14±17.83 |  | 6 |
| 606 | C24H50NO7P | LysoPC 16:0 | Lipids | Steroids | 1 | 224.77±60.41 | 524.79±130.93 | 1124.98±278.09 | 473.61±24.64 |  |  |
| 607 | C8H20NO6P | Choline Alfoscerate | Lipids | Steroids | 3 | 151.59±15.56 | 289.48±37.31 | 464.76±65.02 | 655.88±53.33 |  | 2 |
| 608 | C21H44NO7P | LysoPE 16:0 | Lipids | Steroids | 3 | 42.95±2.4 | 90.21±0.5 | 75.94±19.08 | 82.75±3.09 |  |  |
| 609 | C26H50NO7P | LysoPC 18:2 | Lipids | Steroids | 1 | 857.32±40.41 | 2714.6±200.28 | 2351.95±550.08 | 1351.31±30.1 |  | 6 |
| 610 | C26H54NO7P | LysoPC 18:0 | Lipids | Steroids | 3 | 10.75±5.07 | 13.3±5.63 | 18.1±4.61 | 17.85±4.45 |  |  |
| 611 | C26H48NO7P | LysoPC 18:3 | Lipids | Steroids | 3 | 133.12±9.49 | 232.09±14.39 | 188.39±44.39 | 230.62±60.19 |  |  |
| 612 | C24H48NO7P | LysoPC 16:1 | Lipids | Steroids | 3 | 64.41±14.73 | 153.88±5.66 | 141.05±22.55 | 250.14±11.84 |  | 2 |
| 613 | C22H46NO7P | LysoPC 14:0 | Lipids | Steroids | 3 | 23.56±11.3 | 27.65±1.81 | 36.47±10.46 | 32.54±2.89 |  |  |
| 614 | C18H32O3 | 9(10)-EpOME;(9R,10S)-(12Z)-9,10-Epoxyoctadecenoic acid | Lipids | Fatty acids and their derivatives | 1 | 14.89±1.39 | 14.23±1.78 | 13.15±1.9 | 0±0 |  | 4 |
| 615 | C17H34O2 | Heptadecanoic acid | Lipids | Fatty acids and their derivatives | 3 | 0±0 | 0±0 | 36047.47±1145.94 | 43644.71±2461.15 |  | 3 |
| 616 | C7H12O4 | Pimelic acid* | Lipids | Fatty acids and their derivatives | 3 | 9.9±0.37 | 12.62±2.03 | 19.95±2.33 | 43.77±1.22 |  | 1 |
| 617 | C18H30O3 | 17-Hydroxylinolenic acid | Lipids | Fatty acids and their derivatives | 3 | 1569.31±105.45 | 3392.9±50.88 | 3782.72±384.48 | 4412.37±123.83 |  | 3 |
| 618 | C18H36O2 | Stearic Acid | Lipids | Fatty acids and their derivatives | 2 | 10827.23±6025.29 | 5846.35±376.79 | 6560.49±1462.36 | 4539.54±428.36 |  |  |
| 619 | C20H38O2 | Eicosenoic acid | Lipids | Fatty acids and their derivatives | 2 | 518.98±33.06 | 256.59±17.47 | 271.63±54.92 | 457.39±62.33 |  |  |
| 620 | C18H39NO2 | Dihydrosphingosine | Lipids | Fatty acids and their derivatives | 3 | 0±0 | 24.45±2.62 | 57.75±18.54 | 45.77±3.9 |  |  |
| 621 | C18H38O | 1-Octadecanol | Lipids | Fatty acids and their derivatives | 3 | 8.9±0.7 | 7.43±0.63 | 3.5±0.63 | 4.88±0.67 |  | 4 |
| 622 | C18H36O3 | 12-Hydroxyoctadecanoic acid | Lipids | Fatty acids and their derivatives | 2 | 182.26±12.97 | 212.65±19.37 | 190.88±5.18 | 495.18±30.73 |  | 1 |
| 623 | C18H32O5 | 9,12,13-Trihydroxy-10,15-octadecadienoic acid | Lipids | Fatty acids and their derivatives | 3 | 22.95±2.37 | 38.41±2 | 42.05±2.55 | 49.86±3.26 |  | 3 |
| 624 | C18H34O5 | 6,9,10-Trihydroxyoctadec-7-enoic acid | Lipids | Fatty acids and their derivatives | 3 | 2265.37±40.78 | 3529.35±33.74 | 4433.36±311.45 | 5303.79±31.04 |  | 3 |
| 625 | C14H26O4 | 1,14-Tetradecanedioic Acid | Lipids | Fatty acids and their derivatives | 3 | 38.79±0.57 | 46.83±2.57 | 41.99±2.52 | 53.58±1.86 |  |  |
| 626 | C16H32O2 | Palmitic acid | Lipids | Fatty acids and their derivatives | 3 | 39.22±0.47 | 35.63±4.09 | 41.36±7.88 | 53.71±3.96 |  |  |
| 627 | C20H30O4 | 20-Carboxyarachidonic acid | Lipids | Fatty acids and their derivatives | 3 | 637±39.58 | 1161.91±36.59 | 1159.47±270.26 | 5451.28±115.55 |  | 1 |
| 628 | C18H34O2 | Octadec-11-enoic acid | Lipids | Fatty acids and their derivatives | 3 | 158.41±31.28 | 304.11±8.32 | 295.02±74.09 | 569.58±53.07 |  |  |
| 629 | C20H38O2 | Paullinic acid | Lipids | Fatty acids and their derivatives | 3 | 119.97±93.94 | 135.92±48.91 | 346.6±46.7 | 575.18±44.89 |  |  |
| 630 | C18H34O5 | 9,12,13-TriHOME; 9(S),12(S),13(S)-Trihydroxy-10(E)-octadecenoic acid | Lipids | Fatty acids and their derivatives | 2 | 2591.43±133 | 3843.63±23.89 | 4638.32±140.47 | 5819.3±53.22 | Bitterness | 3 |
| 631 | C18H30O2 | α-Linolenic acid | Lipids | Fatty acids and their derivatives | 1 | 10336.25±1190.2 | 10699.53±420.78 | 6262.09±1361.5 | 6346.64±362.2 | Bitterness | 4 |
| 632 | C17H34O3 | 3-Hydroxy-palmitic acid methyl ester | Lipids | Fatty acids and their derivatives | 1 | 18.69±1.79 | 32.4±2.51 | 45.78±11.09 | 65.34±2.25 |  |  |
| 633 | C8H16O2 | Octanoic acid | Lipids | Fatty acids and their derivatives | 3 | 598.28±26.1 | 769.84±26.99 | 548.89±142.5 | 653.48±38.36 |  |  |
| 634 | C18H36O5 | 9,10,18-Trihydroxystearic acid | Lipids | Fatty acids and their derivatives | 3 | 130.31±6.04 | 224.82±8.48 | 294.86±12.18 | 668.2±5.53 |  | 1 |
| 635 | C18H39NO3 | Phytosphingosine | Lipids | Fatty acids and their derivatives | 3 | 110.15±0.48 | 161.73±10.37 | 535.41±96.87 | 714.16±16.32 |  | 3 |
| 636 | C18H34O3 | Ricinoleic acid | Lipids | Fatty acids and their derivatives | 1 | 208.02±17.91 | 320.91±15.56 | 894.01±82.01 | 739.17±9.73 |  | 3 |
| 637 | C18H32O4 | 7,8-dihydroxyoleic acid | Lipids | Fatty acids and their derivatives | 1 | 112.61±6.69 | 246.49±9.76 | 417.7±68.03 | 752.34±2.17 |  | 2 |
| 638 | C18H34O2 | Oleic acid | Lipids | Fatty acids and their derivatives | 1 | 5166.53±1441.39 | 4916.38±394.98 | 5717.18±2367.48 | 7657.18±1653.08 | Miscellaneous |  |
| 639 | C20H42O | 1-Eicosanol | Lipids | Fatty acids and their derivatives | 3 | 218.25±13.27 | 342.17±9.69 | 976.58±93.79 | 791.94±34.36 |  | 5 |
| 640 | C18H32O3 | 9S-Hydroxy-10E,12Z-octadecadienoic acid | Lipids | Fatty acids and their derivatives | 1 | 422.5±5.7 | 511.6±5.33 | 764.45±147.58 | 805.17±6.37 |  |  |
| 641 | C18H32O3 | alpha-Hydroxylinoleic acid | Lipids | Fatty acids and their derivatives | 1 | 434.81±5.74 | 538.02±9.64 | 813.62±181.87 | 833.54±5.87 |  |  |
| 642 | C14H28O3 | 3-Hydroxytetradecanoic acid | Lipids | Fatty acids and their derivatives | 3 | 148.17±8.91 | 196.02±24.56 | 108.69±6.21 | 83.41±1.72 |  | 4 |
| 643 | C18H35NO | Octadec-2-enamide | Lipids | Fatty acids and their derivatives | 1 | 6336.74±535.81 | 9326.46±2428.71 | 9197.07±700.65 | 8685.58±3183.61 |  |  |
| 644 | C18H30O4 | 13(s)-hydroperoxy-(9z,11e,15z)-octadecatrienoic acid | Lipids | Fatty acids and their derivatives | 1 | 5750.87±702.28 | 3917.52±263.79 | 1154.38±272.43 | 878.53±51.4 |  | 4 |
| 645 | C16H32O3 | 2-Hydroxyhexadecanoic acid | Lipids | Fatty acids and their derivatives | 1 | 2163.8±56.4 | 5398.51±320.11 | 5923.04±1254.94 | 9035.97±223.99 |  | 2 |
| 646 | C13H26O2 | Tridecanoic Acid | Lipids | Fatty acids and their derivatives | 3 | 9.56±0.69 | 10.34±2.22 | 7.14±0.57 | 9.85±0.29 |  |  |
| 647 | C18H28O3 | 12-Oxo-phytodienoic acid | Lipids | Fatty acids and their derivatives | 3 | 13.02±2.19 | 13.59±0.65 | 15.15±2.21 | 10.95±0.42 |  |  |
| 648 | C20H32O3 | 12-Hydroxyicosa-5,8,10,14-tetraenoic acid | Lipids | Fatty acids and their derivatives | 3 | 1168.84±68.33 | 3013.81±74.44 | 4524.22±888.99 | 11085.64±132.37 |  | 1 |
| 649 | C16H32O4 | 9,16-Dihydroxypalmitic acid | Lipids | Fatty acids and their derivatives | 2 | 0±0 | 40.24±3.84 | 104.42±22.09 | 113.18±5.6 |  | 3 |
| 650 | C10H20O3 | 2,6-Dimethyl-7-octene-2,3,6-triol | Lipids | Fatty acids and their derivatives | 3 | 317.5±9.68 | 511.26±24.62 | 667.79±79.85 | 1151.31±10.14 |  | 2 |
| 651 | C18H32O4 | 9-Hydroxy-12-oxo-15(Z)-octadecenoic acid | Lipids | Fatty acids and their derivatives | 1 | 94.68±3.21 | 167.08±4.07 | 221.13±6.21 | 129.13±3.43 |  | 5 |
| 652 | C18H28O | 9,11,13,15-Octadecatetraenal | Lipids | Fatty acids and their derivatives | 3 | 96.23±8.5 | 193.44±8.42 | 303.69±54.48 | 1296.8±52.12 |  | 1 |
| 653 | C18H35NO | Oleamide (9-Octadecenamide) | Lipids | Fatty acids and their derivatives | 1 | 1167.84±103.09 | 1668.66±412.64 | 1530.47±191.93 | 1309.61±507.99 |  |  |
| 654 | C20H30O3 | 5-Oxoicosa-6,8,11,14-tetraenoic acid | Lipids | Fatty acids and their derivatives | 3 | 101.1±5.54 | 190.48±4.11 | 465.33±116.85 | 1391.61±47.81 |  | 1 |
| 655 | C12H24O2 | Lauric acid* | Lipids | Fatty acids and their derivatives | 1 | 13387.29±445.91 | 16522.5±314.19 | 12321.18±1136.67 | 14242.7±143.59 |  | 6 |
| 656 | C18H37NO | Stearamide | Lipids | Fatty acids and their derivatives | 1 | 679.97±112.03 | 971.9±161.26 | 953.06±79.84 | 1459.66±400.14 |  |  |
| 657 | C18H33NO | Octadecadienamide | Lipids | Fatty acids and their derivatives | 1 | 1388.65±225.43 | 1991.03±513.15 | 1849.39±230.95 | 1513.49±530.81 |  |  |
| 658 | C18H32O4 | 9S-Hydroperoxy-10E,12Z-octadecadienoic acid | Lipids | Fatty acids and their derivatives | 3 | 35.63±2.78 | 97.37±5.08 | 165.48±37.69 | 164.92±19.03 |  |  |
| 659 | C16H32O | Palmitaldehyde | Lipids | Fatty acids and their derivatives | 3 | 5596.82±76.77 | 3824.33±390.15 | 2141.2±187.76 | 1755.2±85.71 |  | 4 |
| 660 | C18H34O5 | 9,10-Dihydroxy-12,13-epoxyoctadecanoic acid | Lipids | Fatty acids and their derivatives | 2 | 582.67±6.07 | 1063.7±28.77 | 1499.91±105.44 | 1761.05±5.18 |  | 3 |
| 661 | C11H20O2 | 10-Undecenoic acid | Lipids | Fatty acids and their derivatives | 3 | 36.44±2.16 | 18.61±5.56 | 11.09±0.1 | 18.31±0.74 |  | 4 |
| 662 | C16H30O2 | Hexadec-9-enoic acid | Lipids | Fatty acids and their derivatives | 3 | 137.84±6.58 | 227.81±44.03 | 201.59±32.94 | 195.33±27.7 |  |  |
| 663 | C19H32O2 | Methyl linolenate | Lipids | Fatty acids and their derivatives | 3 | 454.7±27.91 | 954.87±34.38 | 1488.06±163.99 | 2298.96±56.43 |  | 2 |
| 664 | C18H34O4 | Hydroxy ricinoleic acid | Lipids | Fatty acids and their derivatives | 1 | 1142.07±110.81 | 2191.07±30.44 | 2514.07±248.96 | 2308.41±36.25 |  | 5 |
| 665 | C16H32O3 | 3-Hydroxyhexadecanoic acid | Lipids | Fatty acids and their derivatives | 3 | 365.63±15.18 | 696.82±78.62 | 300.09±8.74 | 241.02±6.96 |  | 6 |
| 666 | C11H22O2 | Undecylic Acid | Lipids | Fatty acids and their derivatives | 3 | 2746.57±152.9 | 2125.41±110.87 | 1386.5±47.38 | 2563.13±128.84 |  | 4 |
| 667 | C18H34O2 | 11-Octadecanoic acid(Vaccenic acid) | Lipids | Fatty acids and their derivatives | 1 | 1893.71±672.56 | 1753.81±143.88 | 1879.71±851.17 | 2661.21±613.55 |  |  |
| 668 | C16H32O3 | 16-Hydroxyhexadecanoic acid | Lipids | Fatty acids and their derivatives | 3 | 10.97±1.14 | 9.73±0.81 | 18.17±3.74 | 27.95±1.06 |  | 2 |
| 669 | C15H30O2 | 13-methylmyristic acid | Lipids | Fatty acids and their derivatives | 3 | 423.72±47.22 | 430.8±230.93 | 204.96±49.21 | 281.8±18.27 |  |  |
| 670 | C18H30O2 | 6,10,14-Trimethylpentadeca-5,9-Diene-2,13-Dione | Lipids | Fatty acids and their derivatives | 1 | 221.4±11.75 | 218.36±5.37 | 154.81±91.68 | 289.87±47.28 |  |  |
| 671 | C20H39NO2 | N-Oleoylethanolamine | Lipids | Fatty acids and their derivatives | 1 | 50.35±1.8 | 131.27±5.31 | 232.71±56.3 | 301.57±11.17 |  | 3 |
| 672 | C18H30O2 | Punicic acid (9Z,11E,13Z-octadecatrienoic acid) | Lipids | Fatty acids and their derivatives | 3 | 1098.44±408.8 | 1206.63±3.02 | 2510.5±707.33 | 3110.01±41.85 |  |  |
| 673 | C18H30O3 | 9-Oxo-10,12-Octadecadienoic Acid | Lipids | Fatty acids and their derivatives | 2 | 578.27±25.36 | 1348.83±31.29 | 2153.74±346.88 | 3334.57±49.79 |  | 2 |
| 674 | C15H30O2 | Pentadecanoic Acid | Lipids | Fatty acids and their derivatives | 3 | 727.61±131.37 | 517.98±133.32 | 263.85±58.32 | 342.93±9.05 |  |  |
| 675 | C16H33NO | Hexadecanamide | Lipids | Fatty acids and their derivatives | 2 | 2423.44±656.21 | 4006.36±901.05 | 2864.2±458.32 | 3579.63±886.07 |  |  |
| 676 | C18H30O4 | 9-Hydroperoxy-10E,12,15Z-octadecatrienoic acid | Lipids | Fatty acids and their derivatives | 3 | 278.82±48.49 | 222.19±36.77 | 148.46±46.25 | 362.33±30.28 |  |  |
| 677 | C18H34O5 | 9,10,11-Trihydroxy-12-octadecenoic acid | Lipids | Fatty acids and their derivatives | 2 | 163.77±2.59 | 240.09±4.46 | 291.53±12.31 | 376.53±6 |  | 3 |
| 678 | C12H22O4 | Dodecanedioic aicd | Lipids | Fatty acids and their derivatives | 3 | 4.17±0.35 | 5.28±0.65 | 4.96±0.17 | 3.81±0.8 |  |  |
| 679 | C18H34O4 | 9,10-DHOME; (12Z)-9,10-Dihydroxyoctadec-12-enoic acid | Lipids | Fatty acids and their derivatives | 1 | 79.09±2.47 | 116.94±4.41 | 166.64±32.28 | 397.69±17.38 |  | 1 |
| 680 | C20H37NO2 | linoleoyl ethanolamine | Lipids | Fatty acids and their derivatives | 2 | 69.66±2.85 | 249.96±14.48 | 378.71±77.36 | 403.62±7.12 |  | 3 |
| 681 | C18H30O3 | 9-Oxo-10E,12Z-octadecadienoic acid | Lipids | Fatty acids and their derivatives | 3 | 198.11±6.87 | 429.08±6.34 | 460.2±61.16 | 406.66±15.42 |  | 6 |
| 682 | C6H8O4 | 3-Hexenedioic acid | Lipids | Fatty acids and their derivatives | 3 | 1035.94±24.26 | 2915.22±33.47 | 2614.26±390.35 | 4119.53±165.88 |  | 2 |

Note: The values reported in Supplementary Table S1 are relative peak area ratios (metabolite peak area/internal standard peak area) expressed as arbitrary units, not absolute concentrations in µg/L. To ensure transparency, we have classified all identified metabolites into three confidence levels (added to Supplementary Table S1): Level 1 (MS/MS and RT match score ≥ 0.7; 28% confirmed with authentic standards, marked with ‘*’), Level 2 (MS/MS and RT match score 0.5–0.7), and Level 3 (Q1, Q3, RT, DP, CE match without full MS/MS). Across the 682 identified metabolites, the distribution is: Level 1: 136 (20%), Level 2: 138 (20%), Level 3: 408 (60%).


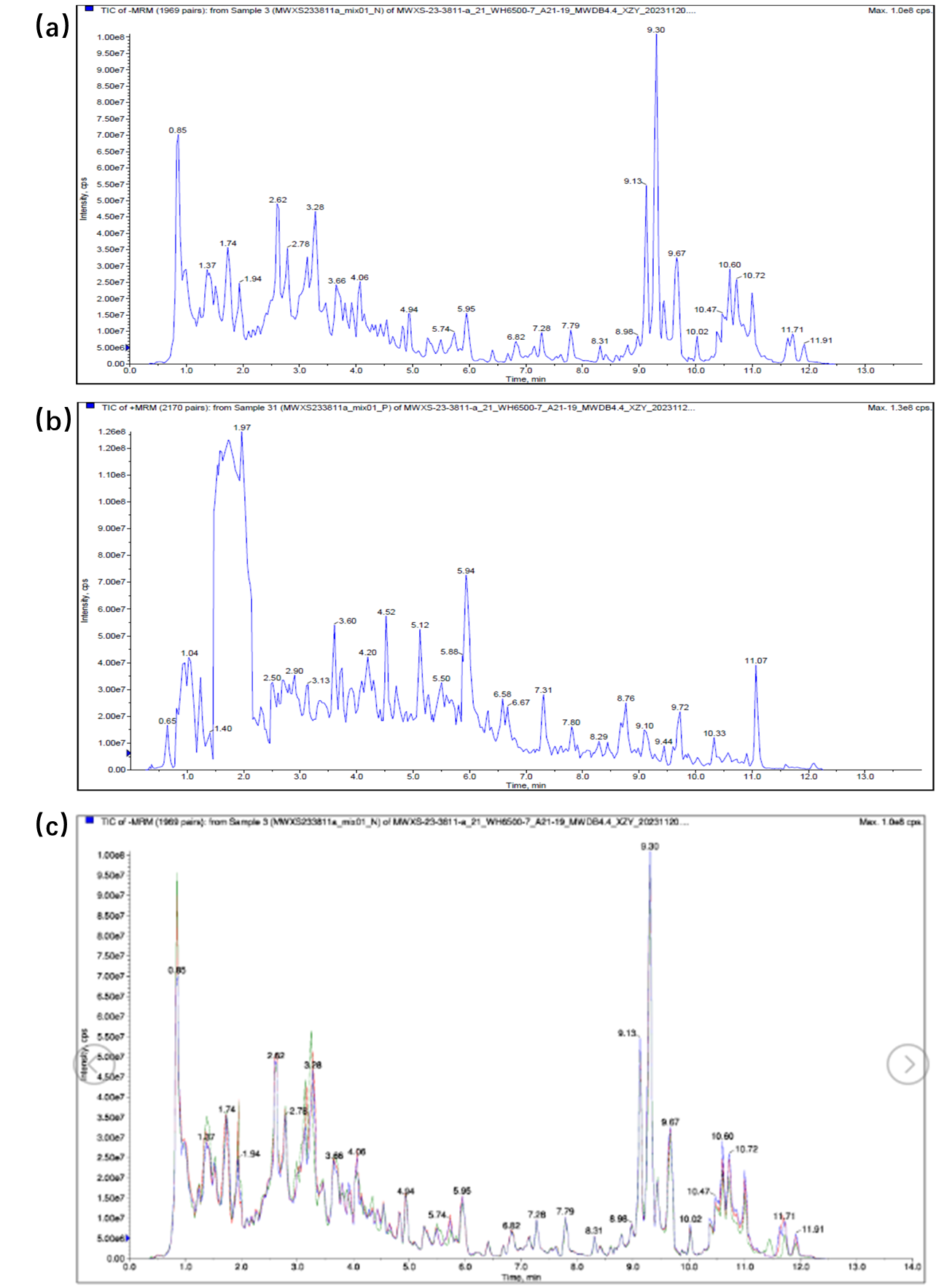


**Figure S1** Representative chromatograms of quality control (QC) samples. (a) Chromatogram peak of QC sample in negative ion mode (ESI–); (b) Chromatogram peak of QC sample in positive ion mode (ESI+–); (c) Overlaid chromatogram peak of QC samples injected at the beginning, middle, and end of the analytical sequence in negative ion mode (ESI–), demonstrating retention time consistency and signal stability throughout the LC–MS/MS run.


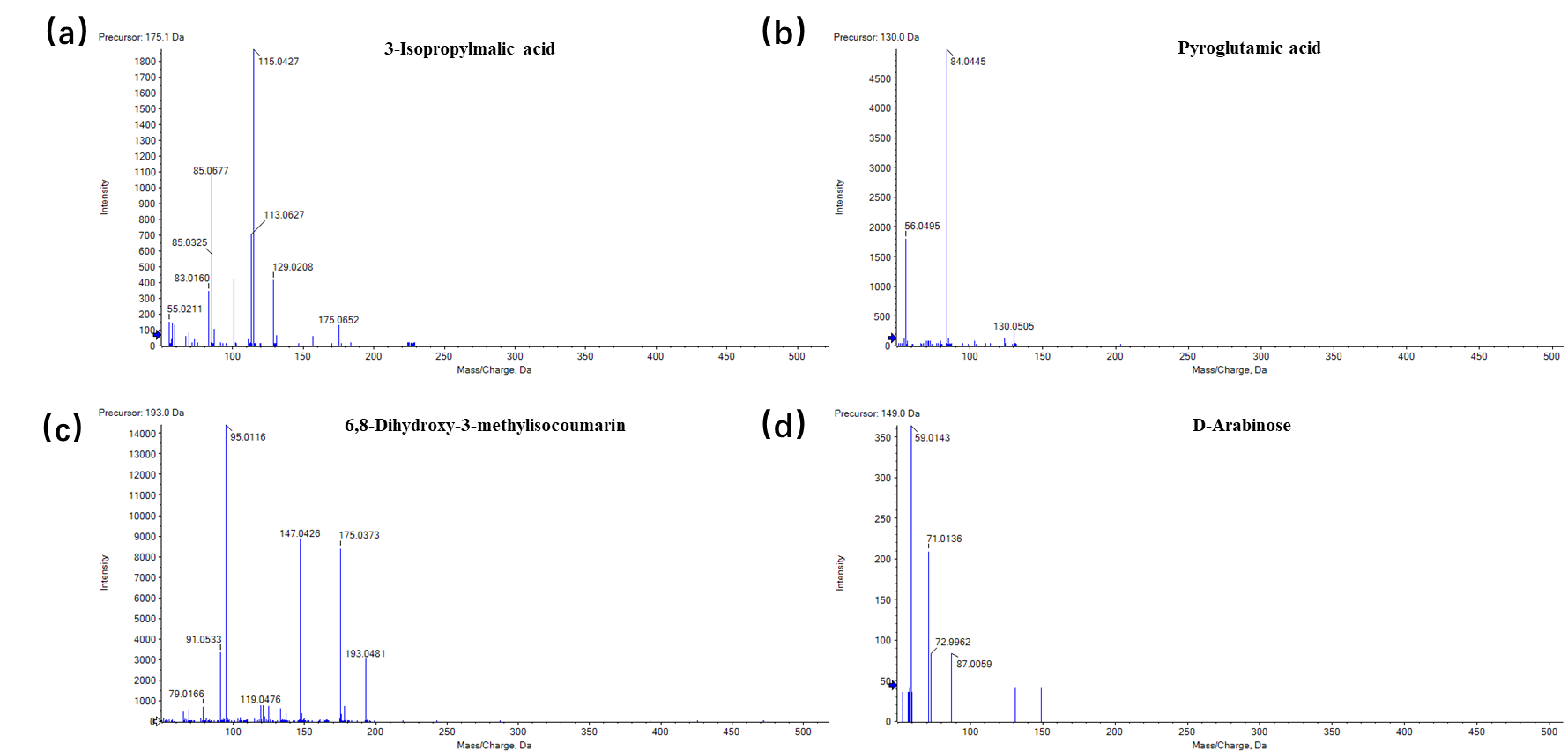


**Supplementary Figure S2** Representative mass spectra of key brandy metabolites. (a) Representative MS/MS spectrum of 3-isopropylmalic acid (Level 1 identification, organic acids and their derivatives) with annotated fragment ions. (b) Representative MS/MS spectrum of pyroglutamic acid (Level 1 identification, amino acids and their derivatives) with annotated fragment ions. (c) Representative MS/MS spectrum of 6,8-dihydroxy-3-methylisocoumarin (Level 2 identification, phenols) with annotated fragment ions. (d) Representative MS/MS spectrum of a representative D-arabinose (Level 1 identification, saccharides and their derivates) with annotated fragment ions.
